# Effects and mechanisms of engineered exosomes pretreated with Scutellaria baicalensis Georgi on osteoporosis

**DOI:** 10.1371/journal.pone.0333897

**Published:** 2025-10-27

**Authors:** Chengbin Long, Xinhang Li, Yueshen Fan, Yingkun Hu, Wenge He, Ning Hu, Fang He

**Affiliations:** 1 Department of Orthopedics, Bishan Hospital of Chongqing Medical University, Chongqing, P.R. China; 2 Department of Orthopedics, The First Affiliated Hospital of Chongqing Medical University, Chongqing, P.R. China; 3 Orthopedic Laboratory, Chongqing Medical University, Chongqing, China; 4 Chongqing Medical University, Chongqing, P.R. China; 5 Department of Nephrology, The First Affiliated Hospital of Chongqing Medical University, Chongqing, P.R. China; Università degli Studi della Campania, ITALY

## Abstract

Osteoporosis (OP) is a common systemic bone disease characterized by reduced bone mineral density and the degeneration of the microstructure of bone tissue. Current therapeutic strategies for OP confront limitations including suboptimal efficacy and substantial adverse effects. This study investigates the therapeutic potential of exosomes derived from bone marrow mesenchymal stem cells (BMSCs) preconditioned with Scutellaria baicalensis Georgi (SBG) for osteoporosis management. Our findings demonstrate that SBG modulates the composition of BMSC-derived exosomes to potentiate osteogenesis. Furthermore, exosomes isolated from SBG-pretreated BMSCs (designated S-EXOs@BMSC) enhance osteogenic differentiation in MC3T3-E1 pre-osteoblasts, a process mediated by upregulating Semaphorin 3A (Sema3A). The efficacy of S-EXOs@BMSC in promoting bone regeneration was corroborated in a murine model of osteoporosis induced by ovariectomy (OVX). These results improve our understanding of how traditional Chinese medicine contributes to OP treatment and emphasize the potential of exosomes from BMSCs grown in SBG conditions as a novel therapeutic strategy for OP.

## Introduction

Osteoporosis (OP) represents a widespread metabolic condition marked by bone fragility, heightened fracture vulnerability, and deterioration of the bone’s microarchitecture [1,2]. This imbalance in bone metabolism predominantly affects postmenopausal women and older individuals [3]. As China’s population rapidly ages, the incidence of OP and its associated risks are escalating. Exercise, along with vitamin and mineral supplements and pharmacological treatments, are established methods for OP management [4]. Nevertheless, the drugs currently used in clinical settings to treat OP, including estrogen, calcitonin, bisphosphonates, and teriparatide, have limited effectiveness and can cause significant side effects [5]. Recent findings indicate that traditional Chinese medicine and other natural products have garnered substantial scientific interest due to their favorable therapeutic outcomes on OP [6,7].

Traditional Chinese medicine has been employed for millennia in China for the prevention and treatment of numerous diseases [8–10]. Presently, a substantial proportion of the population continues to rely on herbal nutritional supplements for primary healthcare. Over half of the drugs currently utilized in clinical practice originate from natural sources [11]. First mentioned in the ‘Shennong Bencao Jing’ around 200 AD, Scutellaria baicalensis Georgi (SBG) has a medicinal history spanning more than 2000 years and demonstrates various pharmacological properties [12–14]. This plant’s dried roots are part of traditional Chinese medicine and are officially noted in the 2020 ‘Chinese Pharmacopoeia’ [15]. Research demonstrates that SBG extract exhibits the capacity to promote alkaline phosphatase production, stimulate osteogenic differentiation of cells, reduce the quantity of RANKL-induced osteoclasts, and diminish inflammatory factor levels [16]. Furthermore, SBG administration for 12 weeks has been reported to alleviate OP in ovariectomized (OVX) murine models [17,18].

Exosomes, measuring between 30 and 150 nm, are extracellular vesicles that can be identified in biological fluids like plasma, urine, and saliva. Virtually all cell types secrete exosomes, whose membranes consist primarily of lipids and proteins, and whose lumina contain abundant bioactive compounds, encompassing proteins, metabolites, lipids, mRNA, microRNA, and other non-coding RNAs [19]. Recent discoveries show that exosomes from bone marrow mesenchymal stem cells (BMSCs) offer therapeutic advantages for various pathological conditions, such as cancer, anti-inflammatory, antioxidative, and anti-diabetic effects [20–22]. After injecting exosomes secreted by normal BMSCs into osteoporotic mice, the OP symptoms of the mice were significantly improved [23]. It has previously been demonstrated that preconditioned exosomes may alter their activity, thereby possessing enhanced efficacy [24].

**Fig 1:**
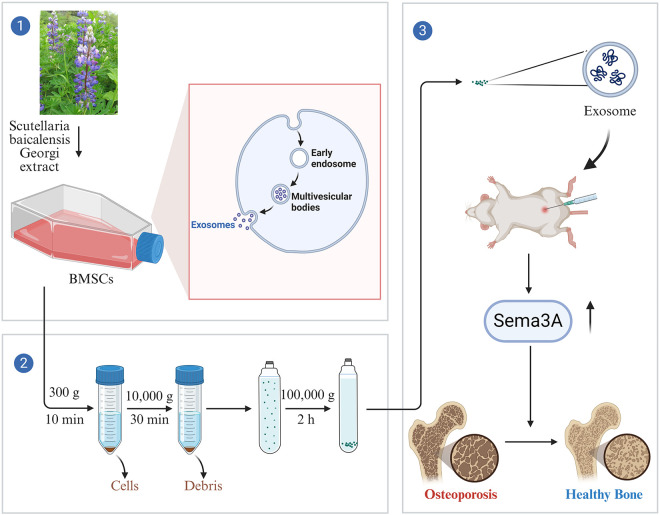
Schematic diagram of S-EXOs@BMSC treating osteoporosis.

Previous research indicates that diverse pretreatment methods for BMSCs, including pharmacological agents, cytokines, and physical factors, effectively enhance their biological activity and functionality in tissue regeneration [25]. Recent studies demonstrate that pretreated BMSCs exhibit augmented paracrine effects. For instance, exosomes derived from hypoxia-preconditioned adipose-derived BMSCs possess superior pro-angiogenic capacity and enhance graft survival compared to those from untreated BMSCs [26]. Similarly, exosomes from BMSCs pretreated with dimethyloxalylglycine (DMOG) significantly promote bone regeneration via enhanced angiogenesis [27]. Furthermore, exosomes isolated from BMSCs preconditioned with baicalin, a flavonoid extracted from Scutellaria baicalensis, ameliorate the disproportion between Th17 and Treg cells and liver damage caused by ischemia-reperfusion injury [28]. Nevertheless, to date, the therapeutic potential of exosomes from BMSCs pre-treated with SBG (S-EXOs@BMSC) for OP remains unexplored. Consequently, this study investigates the effectiveness of the treatment and underlying mechanisms of S-EXOs@BMSC in OP (Fig 1), aiming to provide novel therapeutic strategies and theoretical foundations for OP management. By synergizing the pharmacological properties of SBG with the inherent advantages of BMSC-derived exosomes, we endeavor to establish a safe, efficient, and low-toxicity therapeutic approach for the substantial OP patient population.

## Materials and methods

### Chemical reagents and pharmaceuticals

SBG extract was procured from Shanghai U-Sea Biotech Co., Ltd. (Shanghai, China). The Alkaline Phosphatase (ALP) staining kit, Alizarin Red Staining (ARS) solution, phalloidin and DAPI solution were provided from Beyotime (Shanghai, China). Antibodies targeting RUNX2 and Sema3A were obtained by Proteintech (Wuhan, China). All other reagents were of analytical grade and obtained from standard commercial suppliers unless otherwise specified.

### Isolation of mouse BMSCs

BMSCs was harvested from the femurs and tibias of 3-week-old male C57BL/6 mice (n = 5). Under aseptic conditions, adherent tissues were removed, and the marrow cavities were flushed with α-MEM (Gibco, USA) containing 10% FBS and 1% penicillin-streptomycin. The cell suspension was filtered through a 70-μm strainer (BD Biosciences, USA) and cultured in T-75 flasks at 37°C with 5% CO₂. Non-adherent cells were discarded after 48 h, and the medium was renewed every 3 days until 80–90% confluence. Cells were passaged with 0.25% trypsin-EDTA (Gibco, USA), and passages 3–5 were used for subsequent experiments.

### Exosome extraction

BMSCs were pretreated with 100 μg/mL SBG extract in serum-free α-MEM for 48 h at 37°C with 5% CO₂ to generate S-EXOs@BMSC. The conditioned medium was sequentially centrifuged at 300 × g (10 min), 2,000 × g (20 min), and 10,000 × g (30 min) at 4°C to remove cells and debris. The supernatant was ultracentrifuged at 100,000 × g for 70 min at 4°C (Optima XPN-100, Beckman Coulter, USA), and the pellet was washed once with PBS (pH 7.4) by repeating the ultracentrifugation. The final exosome suspension was quantified by BCA assay (Thermo Fisher Scientific, USA) to 1–2 μg/μL protein. EXOs@BMSC were isolated similarly without SBG pretreatment. All procedures were conducted at 4°C.

### Exosome identification

Exosomes were characterized by transmission electron microscopy (TEM) and nanoparticle tracking analysis (NTA). For TEM, samples (~10^9 particles/mL) were adsorbed on formvar-carbon copper grids (200 mesh; Electron Microscopy Sciences, USA), negatively stained with 2% uranyl acetate, and imaged at 80 kV using a HT7700 TEM (Hitachi, Japan). For NTA, diluted samples were analyzed with a NanoSight NS300 (Malvern Panalytical, UK) and NTA software, recording three 60-s videos per sample.

### Membrane disruption detection of exosomes using Triton X-100

Exosome integrity was evaluated by incubating samples with 0–1% (v/v) Triton X-100 (Sigma-Aldrich, USA) for 20 min at room temperature, followed by NTA analysis. Purity was determined as the proportion of intact particles at 0.1% Triton X-100 relative to controls.

### Exosome detection by flow cytometry

The extracted exosomes were characterized via flow cytometry. Initially, exosomes were immunolabeled with specific antibodies targeting CD63 and CD81. The labeled exosome specimens were subsequently introduced into a flow cytometer for detection utilizing both light scattering and fluorescence signal parameters.

### Biocompatibility testing of exosomes

The CCK-8 assay, based on WST-8, measures cell viability by detecting proliferation and cytotoxicity. It assesses the impact of exosomes on cell growth and survival to evaluate biocompatibility. MC3T3-E1 cells were seeded in 96-well plates at an appropriate density (100 μL cell suspension per well) and cultured for 24 h at 37°C with 5% CO₂ for adhesion. Then, 10 μL exosome solution was added per well, and cells were cultured for 1, 3, or 5 days under the same conditions. The medium was removed, replaced with serum-free medium containing 10% CCK-8 solution, and incubated for 1 h. Absorbance was measured at 450 nm using a microplate reader.

### Immunofluorescence staining

MC3T3-E1 cells were seeded on coverslips (1 × 10^4 cells/well, 24-well plates) and treated with exosomes (50 μg/mL) in osteogenic medium for 5 days. Cells were fixed in 4% PFA-PBS (20 min, room temperature), permeabilized (0.1% Triton X-100, 15 min), and blocked (5% BSA, 30 min). Anti-RUNX2 (1:200; Proteintech) was applied overnight at 4°C, followed by Alexa Fluor 594-secondary antibody (1:200; Invitrogen, USA; 1 h, dark). F-actin (phalloidin, 1:100; Beyotime; 30 min) and nuclei (DAPI, 1:1000; 5 min) were stained. Images were acquired on a Zeiss LSM 880 confocal microscope (Carl Zeiss, Germany) and quantified for intensity using ImageJ.

### Western blotting

Western blotting was used to detect specific protein expression levels. Cells were scraped from culture dishes, lysed with an appropriate amount of lysis buffer, sonicated to disrupt them, and centrifuged at 4°C for 15 min. The supernatant was collected and mixed with loading buffer containing SDS and β-mercaptoethanol, then denatured by heating to 100°C for 10 min. Samples (10 μL protein per well) and a prestained protein marker were loaded. Electrophoresis was performed at 90 V until the front reached the resolving gel, then at 120 V until bromophenol blue reached the gel bottom. The gel was separated, and the target band position was identified using the marker; the corresponding region was excised. Proteins were transferred to PVDF membrane sandwiched with filter paper and sponge (no bubbles) at constant voltage for 60 min. The membrane was blocked in blocking solution with shaking at room temperature for 60 min, incubated with diluted primary antibody overnight at 4°C, and washed three times with TBST (Tris buffer with 0.1% Tween-20) for 5 min each. After incubation with diluted secondary antibody for 1 h at room temperature, the membrane was washed three times with TBST for 10 min each. The membrane was developed with chemiluminescent substrate (ECL) and imaged using a chemiluminescent system.

### Quantitative real-time PCR

Total RNA was isolated from MC3T3-E1 cells using TRIzol (Invitrogen, USA), with purity confirmed by NanoDrop 2000 (Thermo Fisher Scientific, USA). cDNA was reverse-transcribed from 1 μg RNA using PrimeScript RT Kit (Takara, Japan; 37°C, 15 min; 85°C, 5 s). qRT-PCR employed SYBR Green Premix Ex Taq II (Takara) on a QuantStudio 5 (Applied Biosystems, USA): 95°C 30 s, 40 cycles (95°C 5 s, 60°C 30 s). β-actin served as reference; expression was calculated via 2^ − ΔΔCt. Primers are in Table 1.

**Table 1:** Primers sequences utilized for qRT-PCR.

Gene	Forward primer (5’-3’)	Reverse primer (5’-3’)
*β-actin*	GGCTGTATTCCCCTCCATCG	CCAGTTGGTAACAATGCCATGT
*RUNX2*	GCCGGGAATGATGAGAACTA	GGACCGTCCACTGTCACTTT
*ALP*	AACCCAGACACAAGCATTCC	GCCTTTGAGGTTTTTGGTCA
*OCN*	TTGGTGCACACCTAGCAGAC	ACCTTATTGCCCTCCTGCTT
*OPN*	TGCACCCAGATCCTATAGCC	CTCCATCGTCATCATCATCG

### Proteomic analysis of exosomes

Exosomal proteins were extracted using RIPA lysis buffer supplemented with protease inhibitors, and their concentrations were determined with a BCA protein assay (Thermo Fisher Scientific, USA). Equal amounts of protein were reduced and alkylated, and the resulting peptides were analyzed by liquid chromatography–tandem mass spectrometry (LC-MS/MS). Protein identification was performed against the UniProt mouse reference proteome. Label-free quantification (LFQ) was applied to compare the relative protein expression levels between EXOs@BMSC and S-EXOs@BMSC.

### Animal experiments

All animal experiments were approved by the Institutional Animal Care and Use Committee of Chongqing Medical University (Approval No. IACUC-CQMU-2024–0029). Female C57BL/6 mice (8 weeks old, 20–25 g) underwent bilateral ovariectomy (OVX) under isoflurane anesthesia or sham surgery as controls. OVX mice were randomly assigned to three groups receiving intraperitoneal injections of PBS, EXOs@BMSC, or S-EXOs@BMSC every 3 days. After 8 weeks, bone tissues were collected for analysis.When collecting bone tissue, the mice were euthanized by injecting an overdose of sodium pentobarbital.

### Micro-CT analysis

Samples of the femur that had been fixed were placed inside a Micro-CT scanner. The scanning parameters were set, the scanning program was initiated, and high-resolution imaging was performed. The images were reconstructed using the software that came with the Micro-CT scanner to generate a three-dimensional model of the femur. Parameters such as bone volume to total volume ratio (BV/TV), bone mineral density (BMD), and trabecular number (Tb.N) were assessed using analysis software.

### Histological section staining

Decalcified bone tissues were sectioned at 4–5 μm thickness using a microtome, mounted on glass slides, and dried. For H&E and Masson’s trichrome staining (Servicebio, China), sections were processed per standard protocols, dehydrated in 95% and 100% ethanol (15 min each), cleared in xylene (15 min, twice), mounted, and imaged.

For immunofluorescence, sections (4–5 μm) underwent antigen retrieval in citrate buffer (boiling, 10 min), blocking with immunoblocking solution (30 min, room temperature), and subsequent steps as for cells: primary antibody incubation overnight at 4°C, secondary antibody incubation, phalloidin staining, and DAPI staining. Images were acquired using a confocal microscope.

### Data analysis

All results are presented as the mean ± standard error of the mean (SEM), derived from three independent replicate measurements. Inter-group differences were assessed using one-way analysis of variance (ANOVA). Comparisons yielding *p*-values less than 0.05 were considered statistically significant.

## Results

### Exosome characterization and biocompatibility

**Fig 2:**
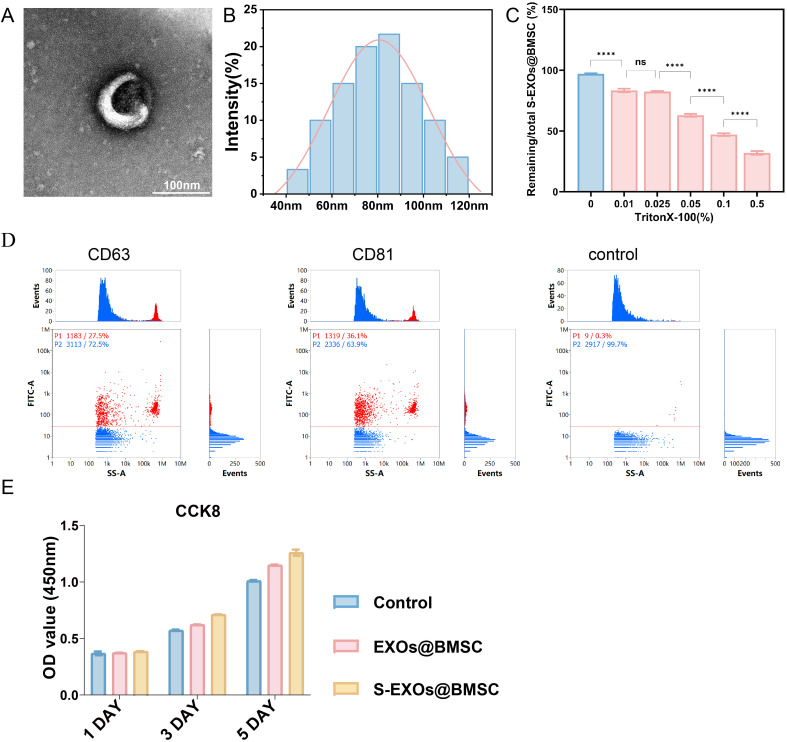
Characterization and biocompatibility assessment of exosomes. (A) Observation of exosomes by TEM. (B) Size distribution profile of the exosomes. (C) The lysis test of exosomes. (D) Flow cytometric analysis of exosome surface markers. (E) Cell viability assessment via CCK-8 assay.

In this research, exosomes were effectively extracted from the culture supernatant of BMSCs that had been pre-treated with the traditional Chinese medicine SBG through differential centrifugation. Differential centrifugation is a widely used technique for exosome isolation, which works by performing multiple centrifugations at different centrifugal forces to gradually remove cells, cell debris, and larger vesicles, ultimately obtaining relatively pure exosomes. This method can effectively recover structurally intact exosomes, laying a solid foundation for subsequent analysis and functional studies. TEM observation clearly revealed that the purified exosomes had a characteristic spherical or cup-like shape with a bilayer membrane structure (Fig 2A). These morphological features are highly consistent with the classic description of exosomes, further confirming that the isolated vesicles are indeed exosomes. The bilayer membrane structure of exosomes is one of their important biological characteristics, not only protecting the bioactive molecules (proteins, nucleic acids, lipids) they carry from degradation by the external environment but also participating in the recognition and fusion process between exosomes and target cells. Therefore, the typical morphological features observed by TEM are one of the important indicators for judging whether exosome isolation is successful. To validate the physical characteristics of the isolated exosomes, NTA was utilized in this study to assess their size distribution. According to NTA results, the exosomes from S-EXOs@BMSC were primarily sized between 40 and 120 nanometers, with a peak at approximately 80 nanometers (Fig 2B). This size range is highly consistent with the typical size of exosomes (usually considered to be between 30 and 150 nanometers), further confirming the successful isolation of exosomes.

In addition, this study used a Triton X-100 disruption experiment to assess the purity of the exosomes. Triton X-100 is a nonionic surfactant that can disrupt the lipid bilayer structure, causing exosomes to rupture and release their internal components. By comparing the changes in specific exosome markers (such as protein concentration or the content of specific proteins) in the samples before and after Triton X-100 treatment, the purity of the exosomes can be indirectly reflected. The experimental results showed that the purity of the isolated SBG-pre-treated exosomes was about 68% (Fig 2C). Although 100% purity is difficult to achieve in biological samples, a purity of 68% indicates that the isolation method can effectively enrich exosomes and minimize contamination from other impurities, providing reliable samples for subsequent functional studies. Exosomes are rich in a variety of specific protein markers on their surface, and the detection of these markers is a key step in identifying exosomes. This study used flow cytometry to detect surface markers on the isolated exosomes. The results showed that S-EXOs@BMSC highly expressed CD63 and CD81, two classic exosome marker proteins (Fig 2D).Belonging to the tetraspanin superfamily, CD63 and CD81 are vital for the creation of exosomes, sorting of cargo, and interactions between cells. The positive expression of these markers further confirmed that the isolated vesicles are exosomes with typical molecular characteristics. In addition to CD63 and CD81, other commonly used exosome markers include TSG101 and Alix, which are usually related to the formation and release processes of exosomes. Accurate and comprehensive identification of exosomes is possible through the combined detection of several markers, which helps eliminate interference from other extracellular vesicles, including microvesicles and apoptotic bodies.

After successfully isolating and identifying exosomes derived from S-EXOs@BMSC, this study further explored their effect on the proliferation of mouse pre-osteoblast MC3T3-E1 cells. For research on osteoblast differentiation, proliferation, and bone metabolism, MC3T3-E1 cells are frequently employed as a cell model. Cell proliferation levels can be quantitatively measured using the CCK-8 assay. According to the experimental results, S-EXOs@BMSC significantly boosted the growth of MC3T3-E1 cells in comparison to exosomes that were not treated (Fig 2E). This finding suggests that after pre-treatment with SBG, the exosomes secreted by BMSCs may carry certain bioactive molecules that can promote osteoblast proliferation. These molecules may act on MC3T3-E1 cells through various pathways, such as activating intracellular pro-proliferative signaling pathways, upregulating the expression of cell cycle-related proteins, or inhibiting apoptosis. Untreated exosomes derived from BMSCs may also have some pro-proliferative activity, which is consistent with other studies reporting that exosomes derived from MSCs have the ability to promote tissue repair and regeneration. However, pre-treatment with SBG further amplifies this pro-proliferative effect, indicating that the active components in SBG may regulate the physiological state of BMSCs, thereby changing the composition and function of the exosomes they secrete. This change may be reflected in the types and quantities of bioactive molecules such as proteins, miRNAs, and lncRNAs carried by exosomes. For example, the active ingredients in Scutellaria baicalensis (such as baicalin and wogonin) may be taken up by BMSCs, thereby affecting the activity of intracellular signaling pathways, regulating gene expression related to the formation of exosomes and the loading of their cargo, ultimately causing an increase in factors that encourage proliferation or a decrease in factors that prevent proliferation in S-EXOs@BMSC. Therefore, the advantage of S-EXOs@BMSC over untreated exosomes in promoting the proliferation of MC3T3-E1 cells provides direct evidence for understanding the potential of traditional Chinese medicine pre-treatment in enhancing the therapeutic efficacy of stem cell exosomes and opens up new ideas for optimizing exosome-based therapeutic strategies.

### The anti-OP efficacy of S-EXOs@BMSC

**Fig 3:**
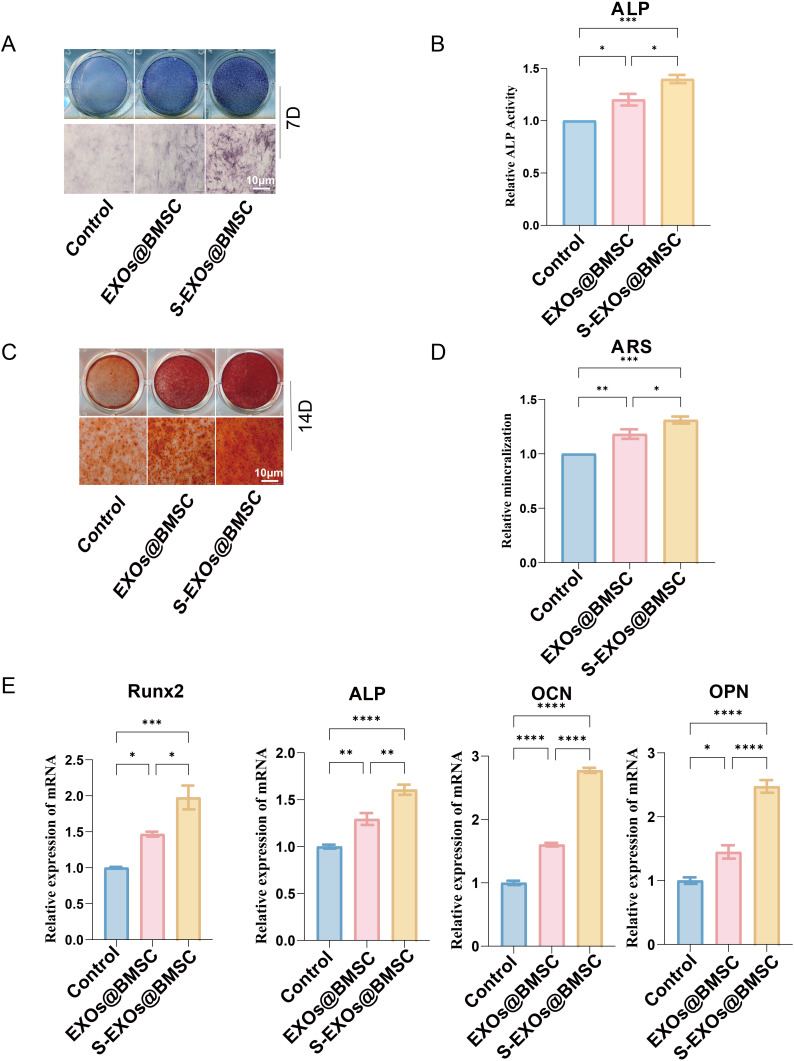
The impact of exosomes on osteogenic differentiation. (A) The ALP staining results at 7 days. (B) Quantitative assessment of ALP staining. (C) The Alizarin Red staining results at 14 days. (D) Quantitative evaluation of Alizarin Red staining outcomes. (E) Markers of osteogenic differentiation were analyzed through quantitative PCR. The following symbols indicate significance levels: * for *p* < 0.05, ** for *p* < 0.01, *** for *p* < 0.001, and **** for *p* < 0.0001.

ALP serves as a crucial enzyme marker for the initial differentiation of osteoblasts, and its activity directly reflects the cells’ ability to differentiate into osteoblasts. After 7 days of osteogenic induction culture, ALP staining and quantitative analysis were performed on MC3T3-E1 cells. The results showed that compared with the control group and the group treated with ordinary BMSC-derived exosomes (EXOs@BMSC), the group treated with S-EXOs@BMSC exhibited a significantly higher percentage of ALP-positive staining area in MC3T3-E1 cells (Fig 3A and 3B). This suggests that S-EXOs@BMSC can significantly boost the activity of MC3T3-E1 cells during the initial phase of osteogenic differentiation, setting the stage for later maturation and mineralization of the extracellular matrix. A rise in ALP activity is often associated with an increased phosphate concentration in the extracellular matrix, which is crucial for forming and mineralizing hydroxyapatite crystals. ARS staining is a technique used to identify calcium nodules that form during the later stages of osteoblast differentiation and is commonly employed to assess the mineralization capability of cells. The cells were subjected to ARS staining after being cultured for 14 days under osteogenic induction. The findings indicated that the S-EXOs@BMSC-treated group developed a notably higher number of calcium nodules in MC3T3-E1 cells (Fig 3C and 3D). Quantitative analysis of ARS staining further confirmed that S-EXOs@BMSC significantly enhanced the mineralization ability of osteoblasts. The formation of calcium nodules is a sign of osteoblast maturation and functional perfection, representing the effective deposition of calcium salts in the extracellular matrix. This result is consistent with the ALP activity test results, jointly proving the promoting effect of S-EXOs@BMSC in the entire osteogenic differentiation process, not only accelerating early differentiation but also significantly improving the level of late mineralization.

To confirm the impact of S-EXOs@BMSC on osteogenic differentiation at the molecular level, qPCR technology was used to measure the mRNA expression levels of several important genes related to osteogenesis. These genes included the core transcription factor Runx2, as well as markers of osteoblast differentiation at different stages, namely ALP, osteocalcin (OCN), and osteopontin (OPN). The data demonstrated that at multiple time points, the mRNA levels of Runx2, ALP, OCN, and OPN in MC3T3-E1 cells treated with S-EXOs@BMSC were considerably greater than in the control group and the EXOs@BMSC-treated group (Fig 3E). Runx2 is the key controller of bone cell differentiation, and its upregulated expression further activates the transcription of a series of downstream osteogenesis-related genes. ALP, OCN, and OPN are key proteins for early, middle, and late stages of osteoblast differentiation and mineralization, respectively. The significant upregulation of these gene expression levels clearly shows that S-EXOs@BMSC can comprehensively boost the osteogenic differentiation pathway of MC3T3-E1 cells at the transcriptional level.

### The mechanism of S-EXOs@BMSC promotes osteogenic differentiation

**Fig 4:**
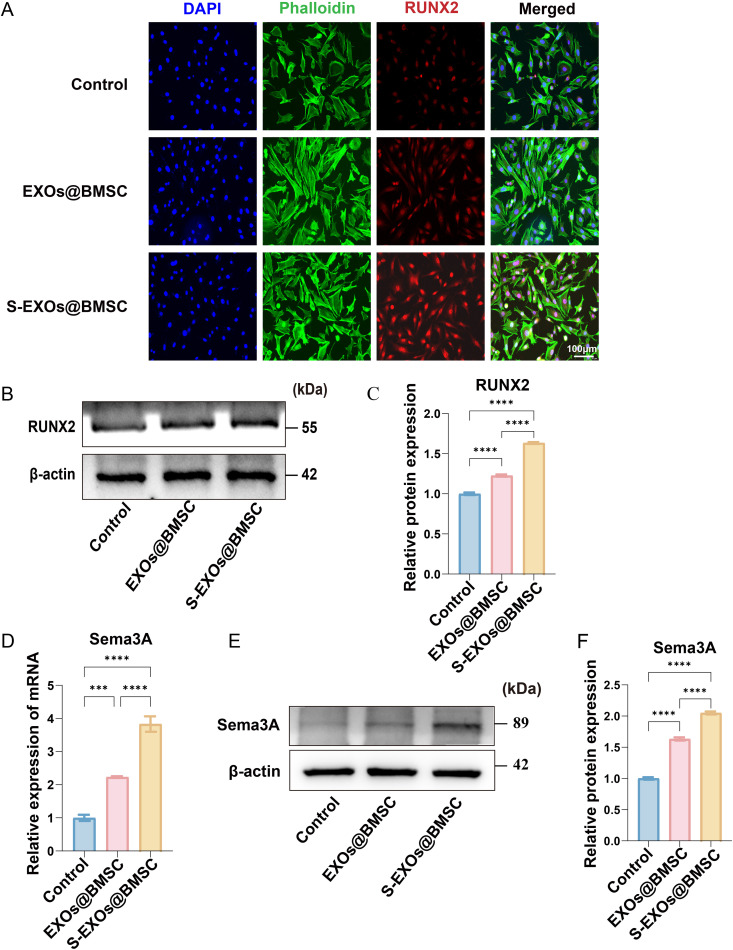
The mechanism by which S-EXOs@BMSC promote the osteogenic differentiation of osteoblasts. (A) RUNX2 immunofluorescence staining. (B) Western blotting of RUNX2. (C) Quantitative results of RUNX2 western blotting. (D) The transcriptional expression level of Sema3A. (E) Western blotting of Sema3A. (F) Quantitative results of Sema3A western blotting. Significance levels are indicated as follows: * for *p* < 0.05, ** for p < 0.01, *** for *p* < 0.001, and **** for *p* < 0.0001.

Runx2 serves as a fundamental regulatory factor in the differentiation of osteoblasts and bone formation, and its expression level directly mirrors the cells’ ability to differentiate in the osteogenic direction. To intuitively observe the effect of S-EXOs@BMSC on Runx2 protein expression in MC3T3-E1 cells, we first performed immunofluorescence staining for Runx2. The experimental findings indicated that MC3T3-E1 cells treated with S-EXOs@BMSC displayed a notably stronger fluorescence signal intensity of Runx2 protein compared to the control group (Fig 4A). This indicates that S-EXOs@BMSC can effectively promote the accumulation of Runx2 protein within MC3T3-E1 cells. Typically, enhanced fluorescence intensity suggests that the target protein is expressed at a higher level or is more densely distributed within the cell. In this experiment, the higher fluorescence intensity directly reflected the enrichment of Runx2 protein in the cell nucleus, which is a key step for Runx2 to exert its transcriptional regulatory activity.

To further quantitatively verify the effect of S-EXOs@BMSC on Runx2 protein expression in MC3T3-E1 cells, we conducted Western blotting experiments. The findings aligned closely with the immunofluorescence staining results, both showing that S-EXOs@BMSC can markedly enhance the expression of Runx2 protein in MC3T3-E1 cells (Fig 4B and 4C). Western blotting detects target proteins using specific antibodies and amplifies and quantitatively analyzes the signals through chemiluminescence methods, yielding results with high accuracy and reliability. By statistically analyzing the band density values, we can precisely compare the differences in Runx2 protein expression among different treatment groups. In this study, the band density value of Runx2 protein in the S-EXOs@BMSC-treated group was significantly higher than that in the control group, which fully demonstrated the upregulation of Runx2 expression by S-EXOs@BMSC at the protein level. This finding aligns with the observed increase in Runx2 mRNA expression at the transcriptional level, reinforcing that S-EXOs@BMSC enhances the osteogenic differentiation of MC3T3-E1 cells through the regulation of Runx2 expression.

Previous literature has reported that SBG can continuously increase the levels of Sema3A in cultured cells [29]. Based on the excellent osteogenic effects of S-EXOs@BMSC and Sema3A’s crucial involvement in osteogenic differentiation documented in the literature, we hypothesized that S-EXOs@BMSC may exert its osteogenic function through the regulation of Sema3A expression in MC3T3-E1 cells. As a key signaling molecule, Sema3A has been demonstrated to facilitate the osteogenic differentiation of BMSCs. To verify this hypothesis, we looked into the influence of S-EXOs@BMSC on Sema3A expression in MC3T3-E1 cells, focusing on transcription and protein levels. To determine if S-EXOs@BMSC facilitate osteogenesis through changes in Sema3A gene expression, we utilized qPCR to assess Sema3A mRNA expression in MC3T3-E1 cells. qPCR is a highly sensitive and specific nucleic acid quantification technique that can accurately reflect changes in gene transcriptional activity. The experimental findings indicated that MC3T3-E1 cells exposed to S-EXOs@BMSC had notably elevated mRNA expression levels of the Sema3A gene compared to the control group (Fig 4D). This indicates that S-EXOs@BMSC can promote the transcription of the Sema3A gene, thereby increasing the synthesis of Sema3A at the mRNA level. The increase in Sema3A mRNA levels is usually a prerequisite for increased protein expression and indirectly suggests that the Sema3A signaling pathway may be activated. The finding is in agreement with our original hypothesis suggesting that S-EXOs@BMSC could aid in the osteogenic differentiation of MC3T3-E1 cells by enhancing Sema3A expression.

After confirming that S-EXOs@BMSC can promote the transcription of the Sema3A gene in MC3T3-E1 cells, we further verified the changes in Sema3A protein expression at the protein level. Using Western blotting, we detected the expression levels of Sema3A protein in MC3T3-E1 cells from different treatment groups. The experimental findings aligned closely with the qPCR results, indicating that the Sema3A protein expression in MC3T3-E1 cells treated with S-EXOs@BMSC was notably elevated compared to the control group (Fig 4E and 4F). Proteins are the ultimate executors of gene function, so the increased expression level of Sema3A protein directly proves that S-EXOs@BMSC can effectively promote the synthesis and accumulation of Sema3A protein in MC3T3-E1 cells. This finding further supports our hypothesis that S-EXOs@BMSC may activate downstream osteogenic differentiation signaling pathways by upregulating the expression of Sema3A. As a secretory protein, the increased expression of Sema3A within the cell may imply a corresponding increase in its release into the extracellular environment, thereby acting on MC3T3-E1 cells themselves or neighboring cells in an autocrine or paracrine manner, thus promoting osteogenic differentiation.

**Fig 5:**
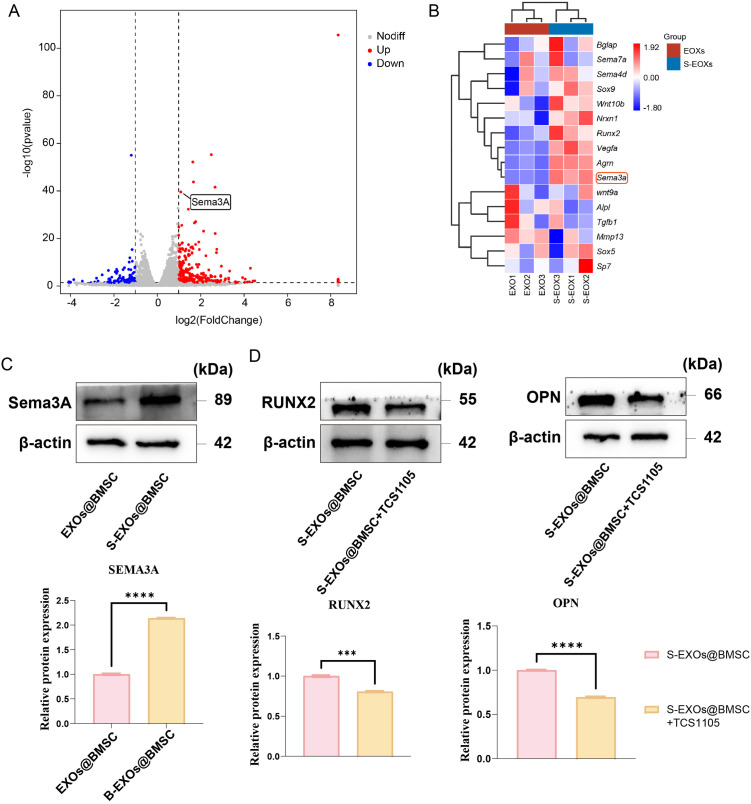
S-EXOs@BMSCs enhance osteogenic differentiation by delivering Sema3A. (A) Volcano plot showing differentially expressed genes. (B) Heatmap illustrating expression patterns of Sema3A-related genes. (C) WB analysis confirming the elevated Sema3A levels in S-EXOs@BMSCs compared with EXOs@BMSCs. (D) Representative images and quantitative analysis demonstrating that inhibition of Sema3A attenuates the osteogenic effects of S-EXOs@BMSCs. Significance levels are indicated as follows: * for p < 0.05, ** for p < 0.01, *** for p < 0.001, and **** for p < 0.0001.

To further verify how S-EXOs@BMSC influence the osteogenic differentiation of MC3T3-E1 cells, we performed proteomic sequencing on S-EXOs@BMSC and EXOs@BMSC. The results showed that, compared to EXOs@BMSC, Sema3A expression was significantly higher in S-EXOs@BMSC (Fig 5A and 5B). We hypothesized that after SBG treatment, S-EXOs@BMSC carry more Sema3A to MC3T3-E1 cells, thereby promoting their osteogenic differentiation. To validate this hypothesis, we further used Western blotting to detect Sema3A content in EXOs@BMSC and S-EXOs@BMSC. The results demonstrated that S-EXOs@BMSC indeed possessed a higher Sema3A concentration (Fig 5C). To further confirm whether the changes in osteogenic effects were due to alterations in Sema3A, we added the Sema3A inhibitor TCS1105 in addition to S-EXOs@BMSC treatment. The results showed that the addition of TCS1105 significantly inhibited the pro-osteogenic effects of S-EXOs@BMSC (Fig 5D). This further validated our hypothesis that S-EXOs@BMSC, by carrying higher concentrations of Sema3A into MC3T3-E1 cells, activate the expression of downstream osteogenic genes.

### In vivo validation of the osteogenic effect of S-EXOs@BMSC in OVX mice

**Fig 6:**
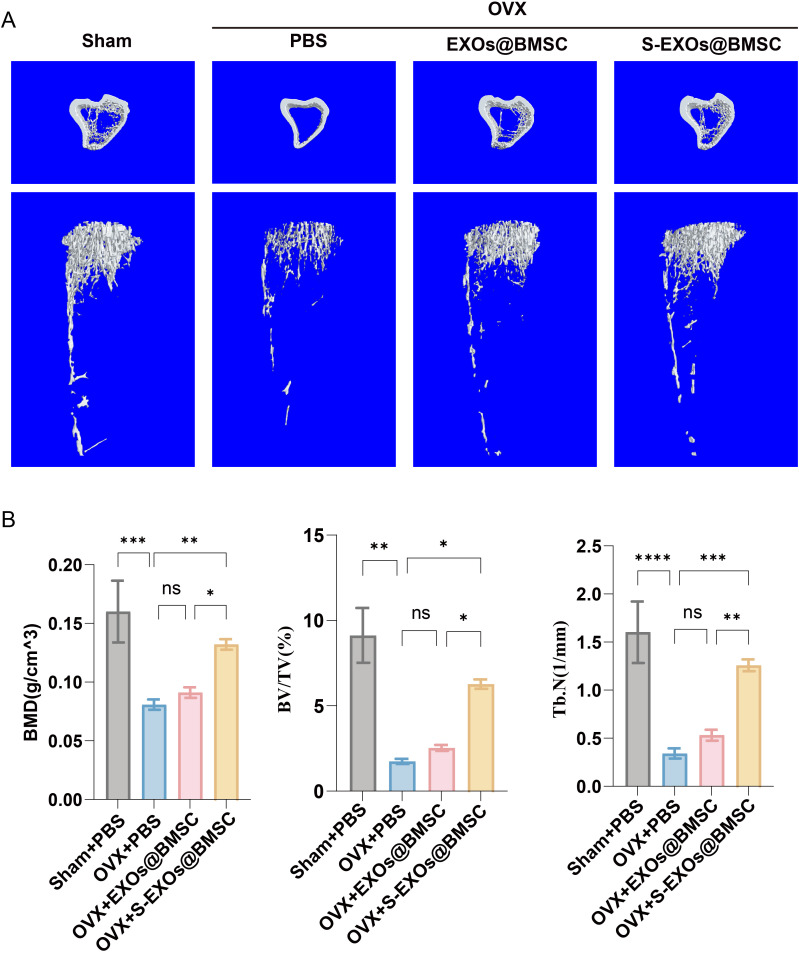
Imaging outcomes of S-EXOs@BMSC therapy in osteoporotic mice. (A) Micro-CT reconstruction results of mouse femurs. (B) Quantitative imaging analysis of mouse femurs. The significance levels are represented as follows: * for *p* < 0.05, ** for *p* < 0.01, *** for *p* < 0.001, and **** for *p* < 0.0001.

The study utilized the OVX mouse model to extensively examine how S-EXOs@BMSC affects BMD. This model, which involves surgically removing the ovaries of female mice, effectively simulates estrogen deficiency and induces the typical phenotypes of postmenopausal OP. The OVX model is widely used in bone formation and repair research because it accurately replicates the pathophysiological changes seen in postmenopausal women because of a significant decrease in estrogen levels, involving an imbalance in bone metabolism, with bone resorption exceeding formation and resulting in rapid bone loss. The femurs of mice, analyzed through micro-CT scanning, demonstrated a notable decrease in BMD in the PBS-treated group relative to the Sham group (Fig 6A and 6B). This result confirmed the successful establishment of the OVX model. The group treated with EXOs@BMSC exhibited only a minor rise in BMD compared to the PBS group, indicating that unmodified BMSC-derived exosomes (EXOs@BMSC) can promote bone formation to some extent, but with limited efficacy. In contrast, the S-EXOs@BMSC-treated group exhibited a much more pronounced bone-forming capacity, significantly increasing the bone volume density (BV/TV), BMD, and trabecular number (Tb.N) in OVX mice. These improvements suggest that S-EXOs@BMSC have superior potential in reversing OVX-induced bone loss, increasing bone mass, and improving bone microstructure. An increase in BV/TV reflects a larger percentage of bone tissue within the overall volume, the elevation in BMD is a direct reflection of increased bone mineral content and better bone density, and the higher Tb.N signifies the restoration and strengthening of trabecular structures. Together, these changes point to the significant potential of S-EXOs@BMSC in treating postmenopausal osteoporosis.

**Fig 7:**
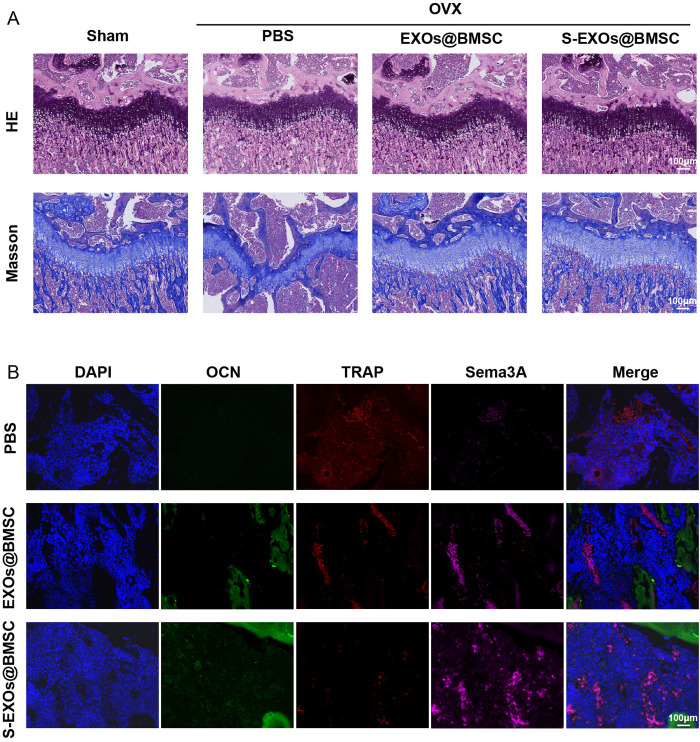
Histological analysis of osteoporotic mice treated with S-EXOs@BMSC. (A) Mouse femurs were analyzed using H&E and Masson’s trichrome staining techniques. (B) The fluorescence staining results of OCN, TRAP, and Sema3A triple co-staining in mouse femurs.

To further evaluate the specific impact of S-EXOs@BMSC on bone tissue repair, this study performed H&E staining and Masson’s trichrome staining on mouse femoral sections to observe the repair of bone defect areas and the formation rate and arrangement of collagen fibers in bone tissue. Differences in trabecular bone structure among the groups were clearly demonstrated by the H&E staining results (Fig 7A). The Sham group mice had well-preserved trabecular bone structure, with tightly packed and orderly arranged trabeculae, presenting a normal bone tissue structure.Conversely, the mice in the PBS-treated group showed a sparse, fragmented, and disorganized trabecular bone structure, which is characteristic of osteoporotic changes induced by OVX, marked by reduced bone mass and damage to bone microstructure. In comparison, the S-EXOs@BMSC-treated group mice showed significant improvement in trabecular bone structure, with increased trabecular number and more orderly arrangement, indicating that S-EXOs@BMSC can effectively promote the process of regenerating and repairing bone tissue, restoring the damaged bone microstructure. The Masson’s trichrome staining results further revealed the changes in collagen fibers within the bone tissue. The PBS-treated group mice had a significant reduction in collagen fiber content in the bone tissue, with loose arrangement, which is consistent with the characteristics of insufficient bone matrix synthesis and increased collagen degradation in osteoporosis. In the EXOs@BMSC-treated group, there was an increase in collagen fiber content and a more compact and orderly arrangement, showing a certain degree of bone repair effect. However, the S-EXOs@BMSC-treated group had the most significant improvement, exhibiting a notably greater collagen fiber content compared to the PBS and EXOs@BMSC groups, and a more tightly packed and regular arrangement. The bone matrix relies heavily on collagen fibers, whose content and organization directly influence the bone’s mechanical strength and overall integrity. The ability of S-EXOs@BMSC to significantly promote the synthesis and orderly arrangement of collagen fibers is of great significance for enhancing bone strength and improving bone quality. In summary, compared with the PBS and EXOs@BMSC groups, S-EXOs@BMSC treatment significantly boosted bone formation speed in OVX mice, promoted the repair of bone defects, and improved the quality of the bone matrix.

To further explore the molecular mechanisms underlying the osteogenic effects of S-EXOs@BMSC, this study conducted immunofluorescence analysis to detect the expression of key osteogenic protein OCN, signaling protein Sema3A, and key osteoclastic protein TRAP in femoral tissues. Compared with the PBS and EXOs@BMSC groups, the fluorescence intensity of OCN and Sema3A in the femurs of S-EXOs@BMSC-treated mice was significantly enhanced, while the fluorescence intensity of TRAP was reduced (Fig 7B). These results are consistent with the increased bone mass and improved bone microstructure observed by Micro-CT and histological staining, further confirming the osteogenic effects of S-EXOs@BMSC. More importantly, the upregulation of Sema3A expression suggests that S-EXOs@BMSC may exert their osteogenic effects by modulating the Sema3A signaling pathway. Sema3A has been shown to enhance the growth, specialization, and mineralization of osteoblasts, while also suppressing the differentiation and function of osteoclasts, thereby playing a crucial role in preserving bone balance. As a result, S-EXOs@BMSC enhance bone formation in OVX mice more efficiently by increasing Sema3A expression.

## Discussion

OP, a systemic degenerative bone disease characterized by diminished bone mass and the deterioration of bone microstructure, poses a significant threat to human health, particularly among the elderly population [30]. The primary treatment medications consist of inhibitors of bone resorption, promoters of bone formation, and traditional Chinese medicine [31]. While estrogen replacement therapy effectively prevents bone loss, its prolonged use is associated with substantial side effects, such as increased risks of cardiovascular events and thromboembolism [32]. In this context, plant-derived compounds like Scutellaria baicalensis Georgi (SBG) have emerged as promising alternatives for OP management, demonstrating anti-inflammatory, antioxidant, and osteogenic properties in preclinical models [33–35]. However, the clinical translation of these natural products remains limited by challenges in bioavailability and targeted delivery.

Exosomes, nanoscale extracellular vesicles (40−150 nm in diameter) secreted by eukaryotic cells, have revolutionized regenerative medicine by serving as natural carriers for intercellular communication [36]. Exosome-derived miRNAs and mRNAs profoundly modulate BMSC osteogenic differentiation, cell-cell interactions, and bone metabolic homeostasis [37]. Notably, BMSC-derived exosomes exhibit excellent biocompatibility, low immunogenicity, and precise targeting capabilities, enabling efficient delivery of bioactive cargos (e.g., proteins, lipids, and non-coding RNAs) to recipient cells, thereby regulating cellular functions with minimal off-target effects [38]. In OP therapy, engineered exosomes have shown remarkable potential. For instance, bone-targeted exosomes delivering siRNA against RANKL have restored bone homeostasis in OVX models by suppressing osteoclastogenesis [2]. Similarly, exosomes derived from Pueraria lobata or oyster mantle have alleviated OP by enhancing autophagy, ferroptosis inhibition, and osteoblast proliferation through pathways like AKT/mTOR and Caveolin-1 interactions [3,5]. Exercise-induced exosomes enriched with FNDC5/irisin further promote osteoblast survival and inhibit ferroptosis, underscoring the therapeutic versatility of exosome-based interventions [4]. These studies highlight how exosomes outperform traditional drugs by mimicking physiological signaling and avoiding systemic toxicity, aligning with our observation that S-EXOs@BMSC exert more direct and potent osteogenic effects on MC3T3-E1 cells. An innovative aspect of our study lies in preconditioning S-EXOs@BMSC, integrating TCM principles with modern exosome technology. Preconditioning strategies, including pharmacological agents like TCM extracts, have been shown to optimize stem cell paracrine effects and exosome cargo composition, thereby amplifying regenerative outcomes [39].

Sema3A is crucial for osteogenesis and bone metabolism [40]. The primary function is to manage osteoblast and osteocyte activity through binding with receptors such as Neuropilin-1 (Nrp-1) [41–43]. Investigations reveal that Sema3A enhances the differentiation of osteoblasts and bone formation by triggering the Wnt/β-catenin signaling pathway [42,43]. In addition, under mechanical loading conditions, Sema3A can also maintain mitochondrial homeostasis within cells through the ROCK2 signaling pathway, thereby promoting bone formation [44]. Furthermore, Sema3A bridges neuro-osteo coupling by guiding axonal innervation to bone cells, influencing sensory nerve-mediated bone metabolism. Dysregulated Sema3A, often downregulated in OP, contributes to imbalanced bone turnover [45]. It primarily regulates the interactions between neurons and bone cells, thereby influencing bone metabolism and the function of the nervous system. Sema3A is a signaling molecule widely present in the nervous system and is involved in the guidance of neuronal axon direction. Further investigation has shown that S-EXOs@BMSC can increase the expression of Sema3A in osteoblasts, supporting bone regeneration. The use of S-EXOs@BMSC might represent an innovative treatment strategy for OP. This research found that S-EXOs@BMSC treatment notably elevated Sema3A expression in the femurs of OVX mice through immunofluorescence analysis. The upregulation of Sema3A expression following S-EXOs@BMSC treatment was highly consistent with the observed increases in bone density, improvements in bone microstructure, and upregulation of the osteogenic marker OCN. The evidence strongly points to the activation of the Sema3A signaling pathway as a partial mediator of the osteogenic effects of S-EXOs@BMSC. Our subsequent studies further demonstrated that S-EXOs@BMSCs facilitate osteogenic differentiation through the transfer of Sema3A into osteoblasts. The elevated levels of Sema3A boost osteoblast differentiation and function and reduce osteoclast activity, ultimately increasing bone mass. Elucidation of this mechanism not only reveals a new pathway for the superior osteogenic effects of S-EXOs@BMSC but also offers a foundational theory for developing therapies targeting the Sema3A signaling pathway for managing osteoporosis and other conditions affecting bones.

This study offers a fresh approach by merging SBG pre-treatment with exosomes from BMSCs to address OP. Leveraging SBG’s pharmacological activities to precondition BMSCs is anticipated to enhance exosome therapy, establishing groundwork for innovative therapeutic strategies. Furthermore, a comprehensive study into the therapeutic potential and mechanisms of exosomes at cellular and molecular levels establishes a robust theoretical foundation for their clinical application in treating OP. More importantly, it provides a new perspective and method for the modernization research of traditional Chinese medicine, combining traditional Chinese medicine with modern biotechnology to explore new pathways for the action of traditional Chinese medicine, and provides a beneficial reference for the innovative application of traditional Chinese medicine.

However, this study also has some limitations. Although it is speculated that the active components in SBG may exert their effects by altering the cargo of exosomes, the specific components involved, the signaling pathways through which they act, how they regulate exosome biogenesis and cargo sorting, and how the modified exosomes are taken up by MC3T3-E1 cells to exert pro-proliferative effects— these key scientific questions still require further in-depth research.

## Conclusion

This study isolated and identified exosomes derived from S-EXOs@BMSC and verified their significant effects in the treatment of osteoporosis. The research discovered that S-EXOs@BMSC significantly enhanced the growth, bone-forming differentiation, and mineralization ability of MC3T3-E1 pre-osteoblasts, which is strongly linked to the increased expression of Sema3A. In in vivo experiments, S-EXOs@BMSC significantly increased bone mass and bone microstructure parameters in ovariectomized mouse models, improving osteoporosis symptoms. These findings introduce a new possible method for treating osteoporosis and offer new research ideas for the combination of traditional Chinese medicine and modern biotechnology. Nonetheless, more detailed studies are required to understand the specific active components and mechanisms of action in S-EXOs@BMSC.

## Supporting information

S1 File The Raw data of the manuscript. (ZIP)
