# Supplementary material for: Effects and mechanisms of engineered exosomes pretreated with Scutellaria baicalensis Georgi on osteoporosis
Source: PLoS One. 2025 Oct 27;20(10):e0333897. doi: 10.1371/journal.pone.0333897 (PMC12558540; doi:10.1371/journal.pone.0333897)
Supplement: S1 File — (ZIP) [file pone.0333897.s001.zip › Raw data/Figure 2/NTA/CD81.pdf]

Concentration Report

2

Data File	20240412 2 17.nfa	Population	Total

SN: FNAN30E20071514
Software: V2.0
Sample Pressure: 1.0Kpa

Laser: 5/40 mW 488
SS Decay: 10%
Threshold/sub: 74.5 9.2 1.1 1/0 0 0 0
Min Width: 0.3 ms

Total Concentration Infomation

	Particle Number	Dilution Factor
STD	4638	100
Blank	100	——
Sample	3655	1

STD Con.	2.22E+10	Particles/mL
Sample Flow Rate	20.89	nL/min
Sample Con.	1.70E+8	Particles/mL

Corrected Ratio: 3555/3555 100.0%

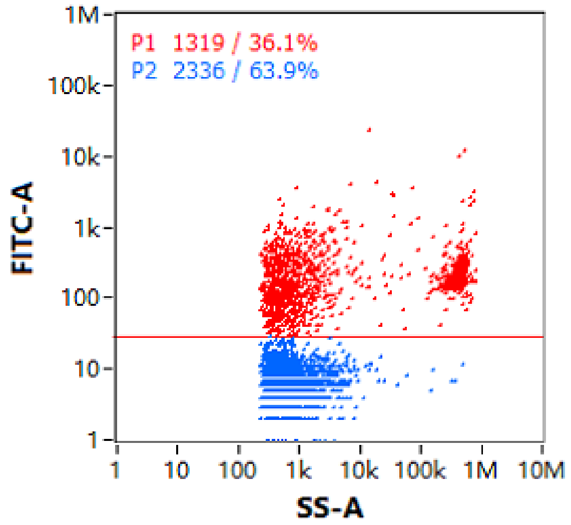

Blank

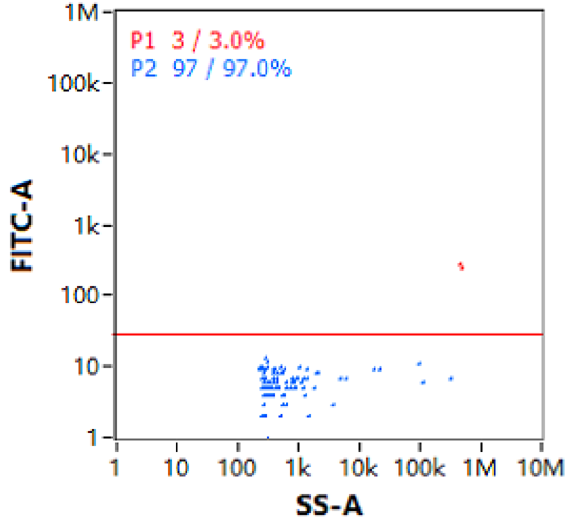

STD

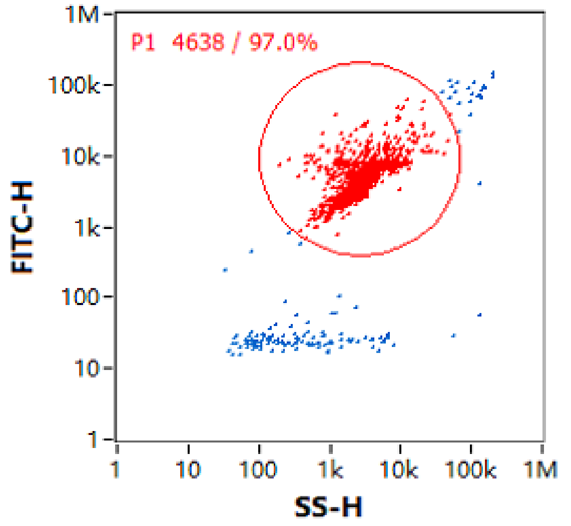

Report By :

2024/4/12 14:34
