# Supplementary figures and images for: Effects and mechanisms of engineered exosomes pretreated with Scutellaria baicalensis Georgi on osteoporosis
Source: PLoS One. 2025 Oct 27;20(10):e0333897. doi: 10.1371/journal.pone.0333897 (PMC12558540; doi:10.1371/journal.pone.0333897)

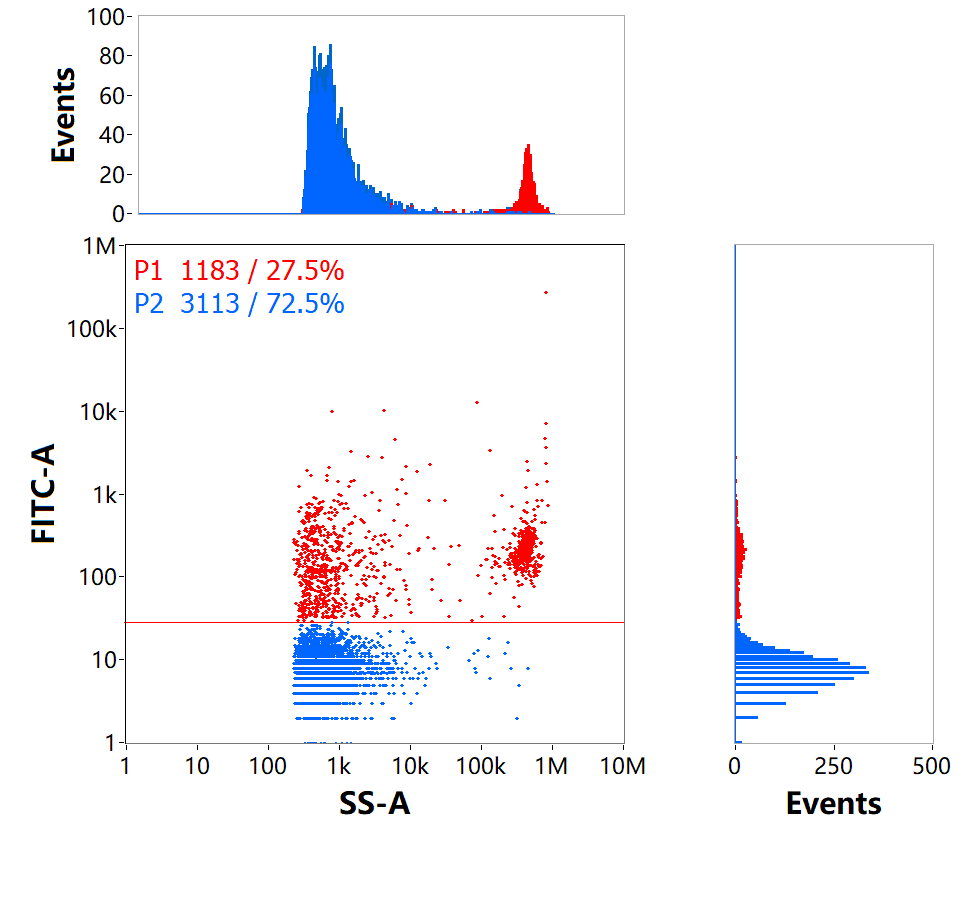

Supplement: S1 File — (ZIP) [file pone.0333897.s001.zip › Raw data/Figure 2/NTA/CD63.bmp]

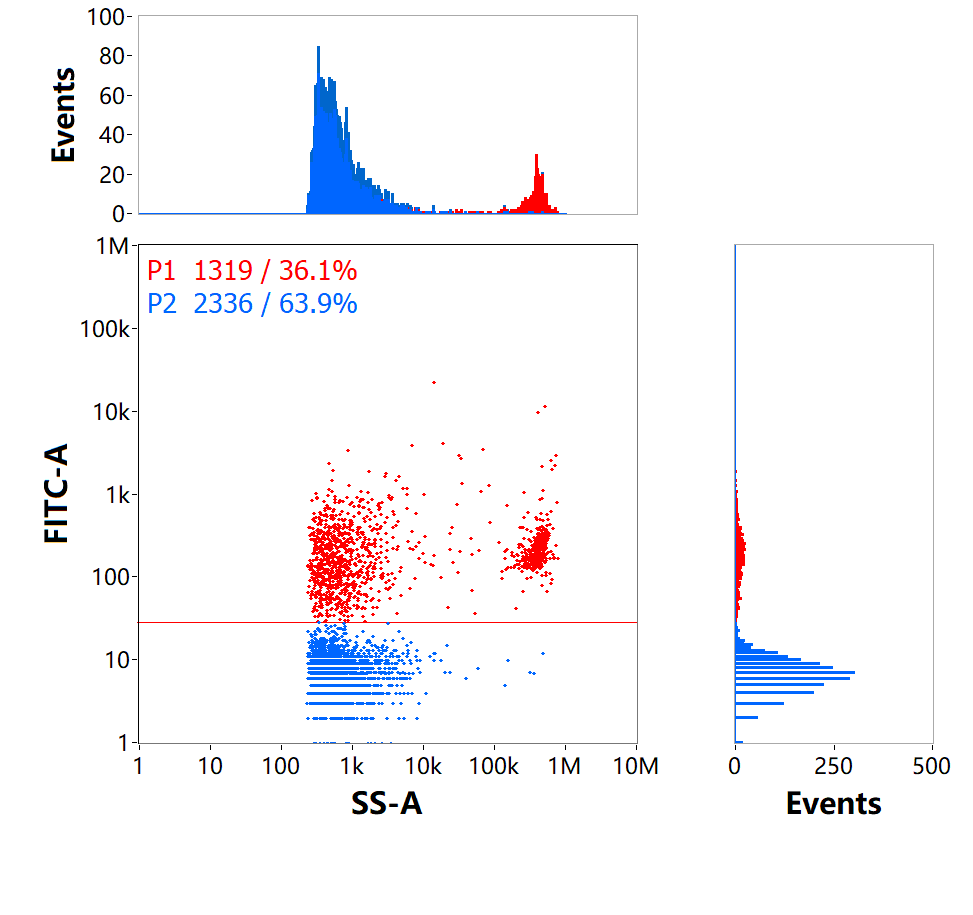

Supplement: S1 File — (ZIP) [file pone.0333897.s001.zip › Raw data/Figure 2/NTA/CD81.bmp]

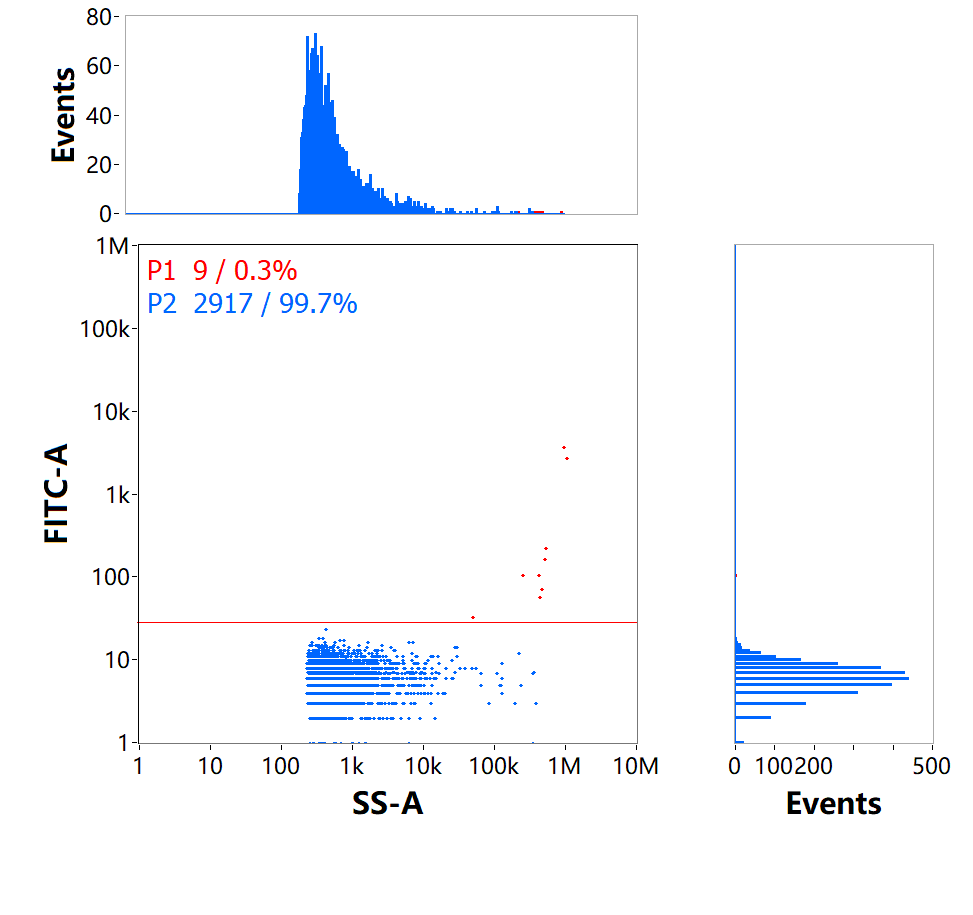

Supplement: S1 File — (ZIP) [file pone.0333897.s001.zip › Raw data/Figure 2/NTA/control.bmp]

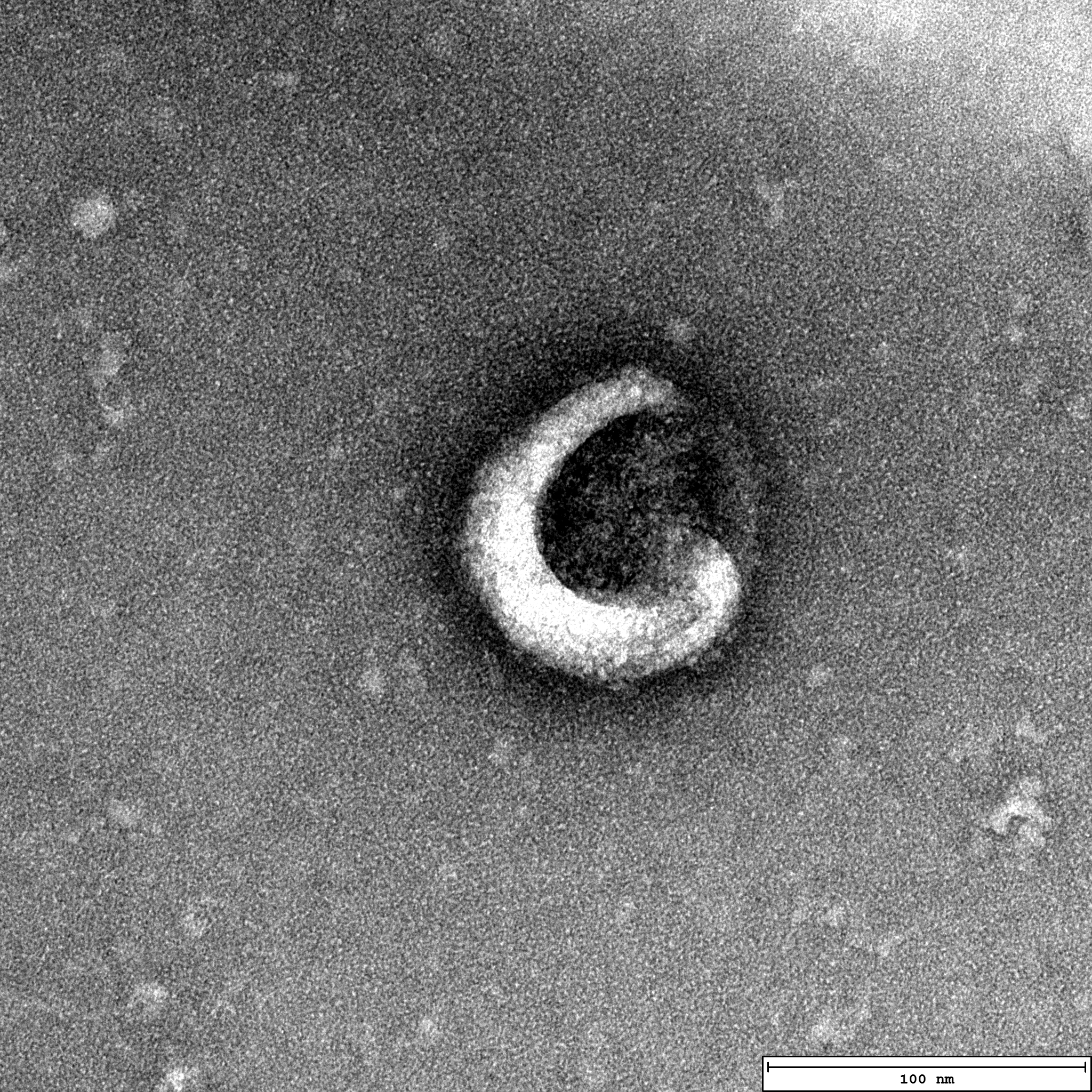

Supplement: S1 File — (ZIP) [file pone.0333897.s001.zip › Raw data/Figure 2/TEM/ZKBC_15935.tif]

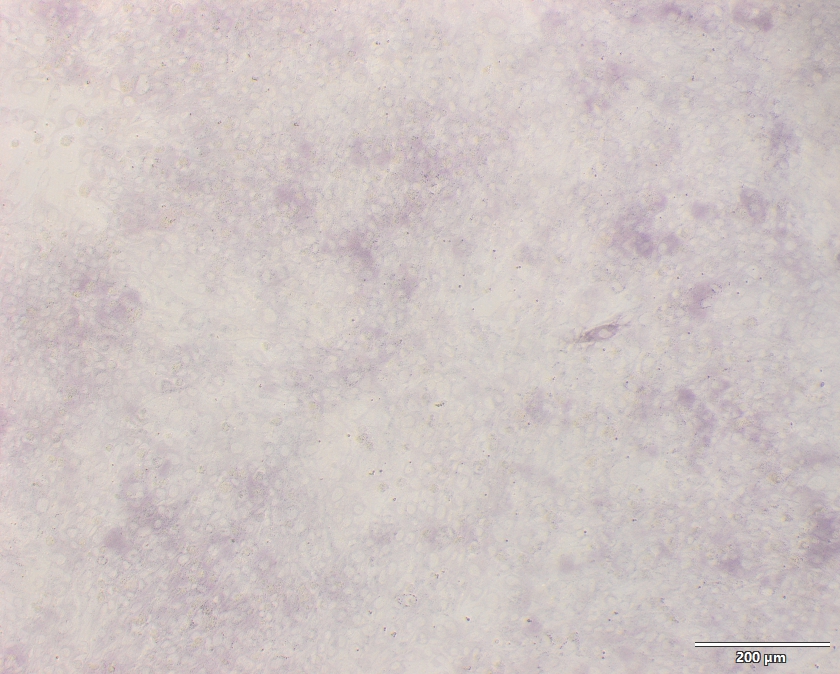

Supplement: S1 File — (ZIP) [file pone.0333897.s001.zip › Raw data/Figure 3/ALP/Control.jpg]

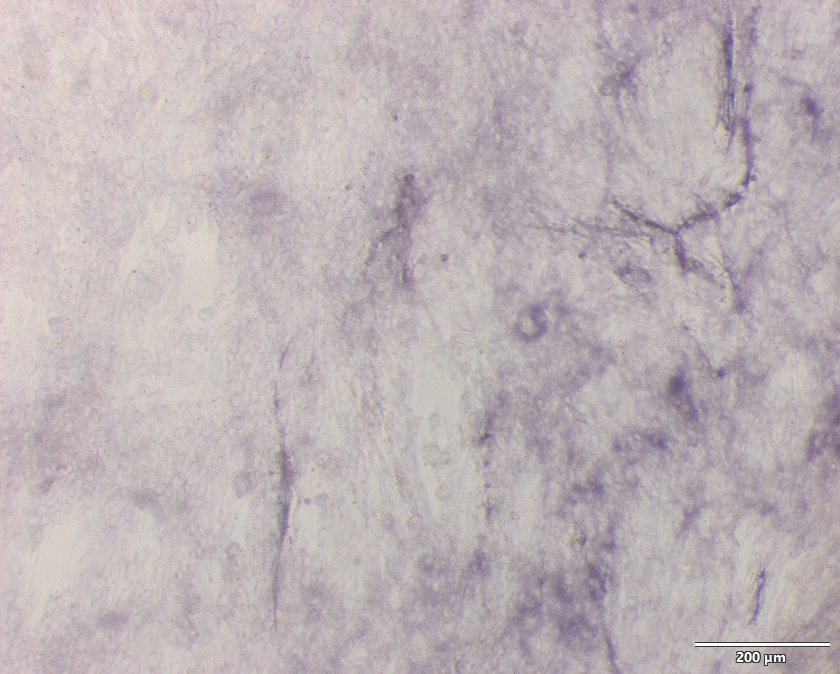

Supplement: S1 File — (ZIP) [file pone.0333897.s001.zip › Raw data/Figure 3/ALP/EXOs.jpg]

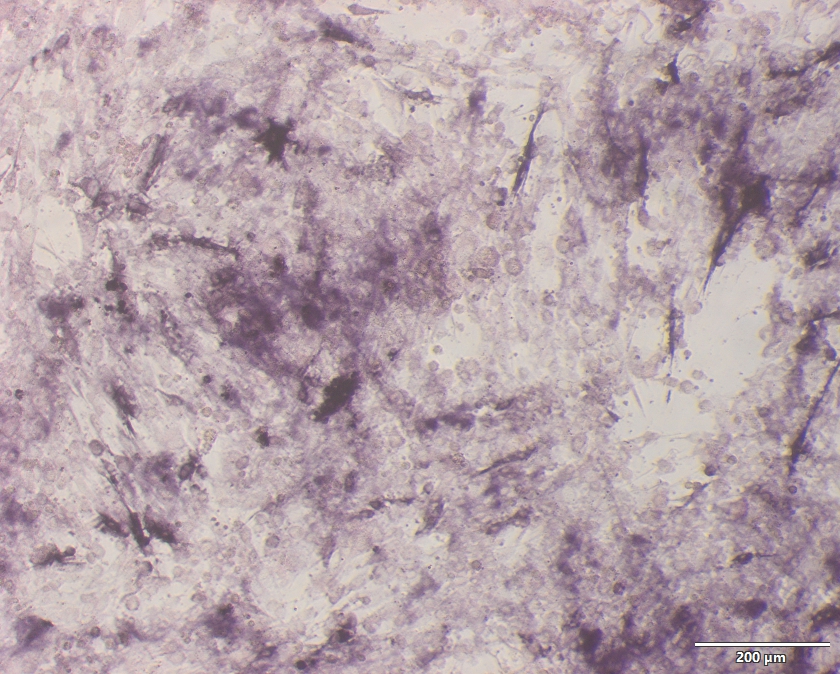

Supplement: S1 File — (ZIP) [file pone.0333897.s001.zip › Raw data/Figure 3/ALP/S-EXOs.jpg]

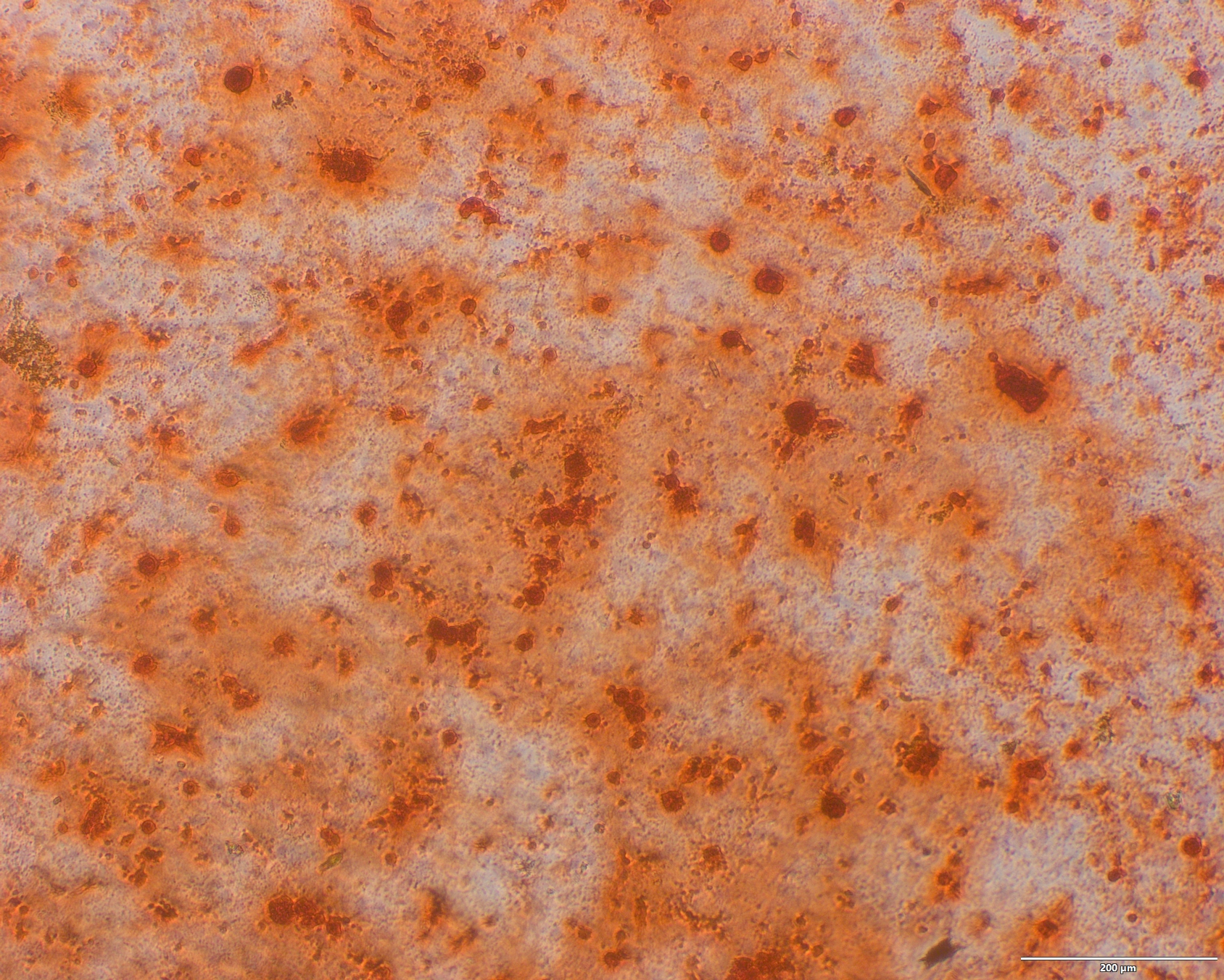

Supplement: S1 File — (ZIP) [file pone.0333897.s001.zip › Raw data/Figure 3/ARS/Control.jpg]

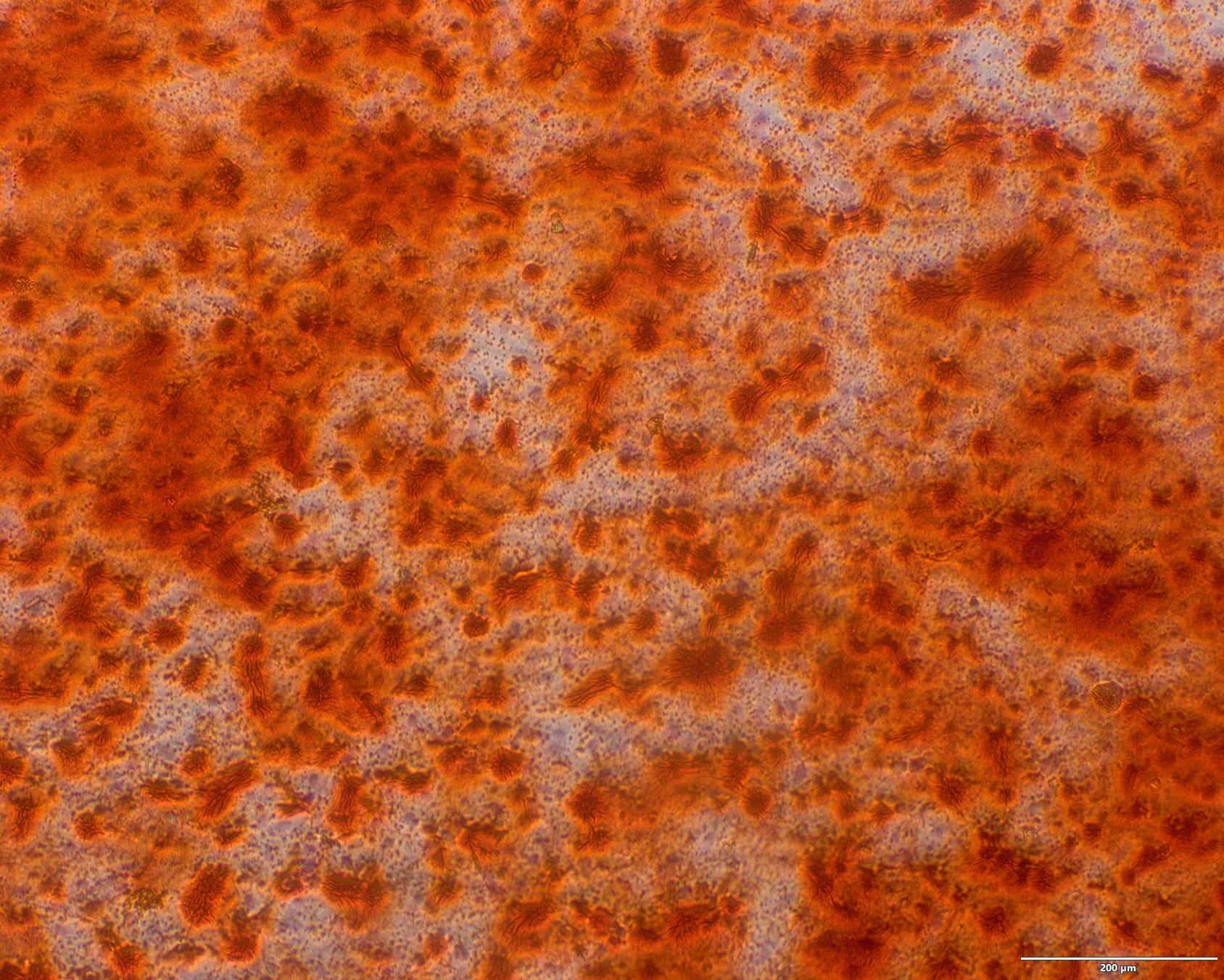

Supplement: S1 File — (ZIP) [file pone.0333897.s001.zip › Raw data/Figure 3/ARS/EXOs.jpg]

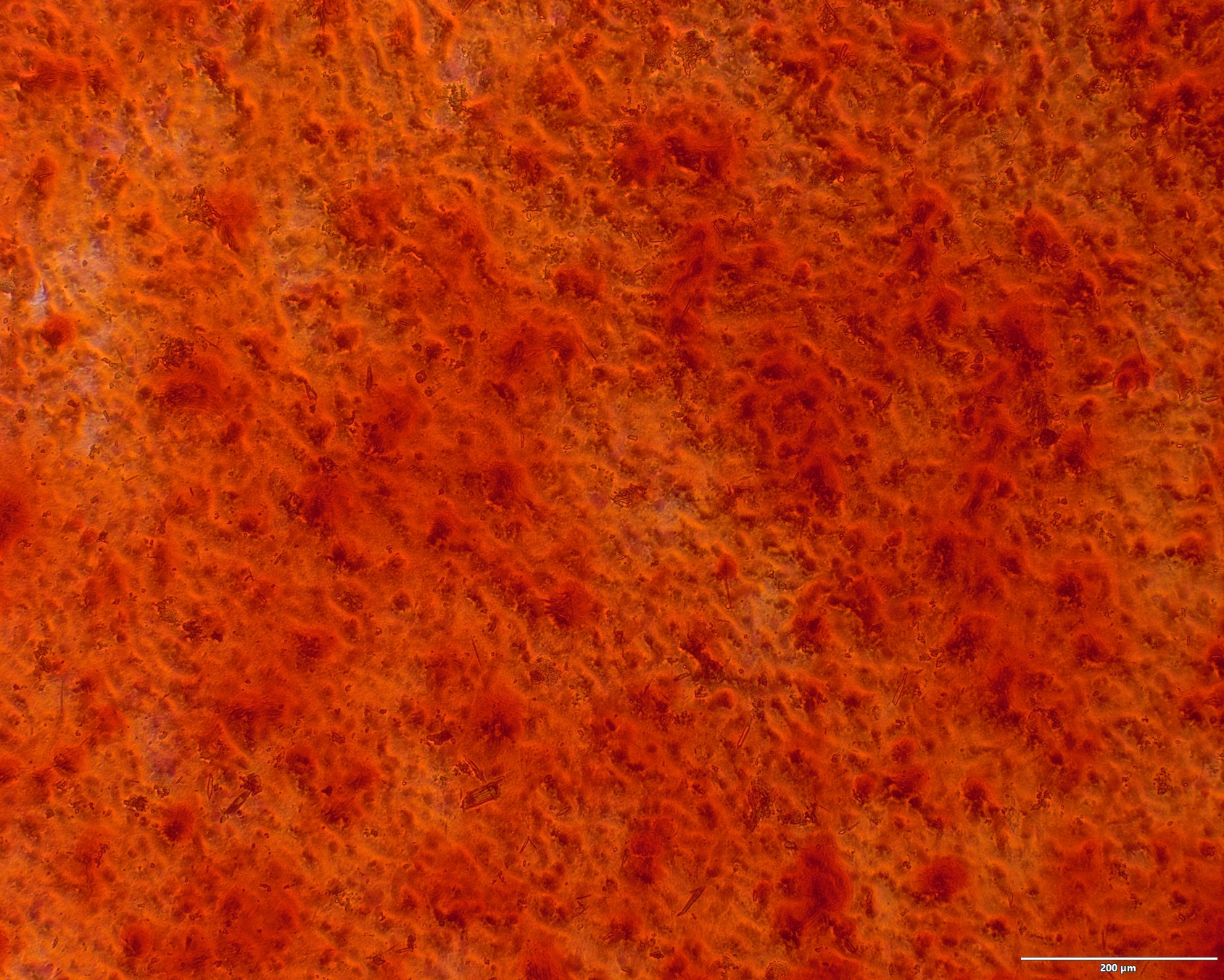

Supplement: S1 File — (ZIP) [file pone.0333897.s001.zip › Raw data/Figure 3/ARS/S-EXOs.jpg]

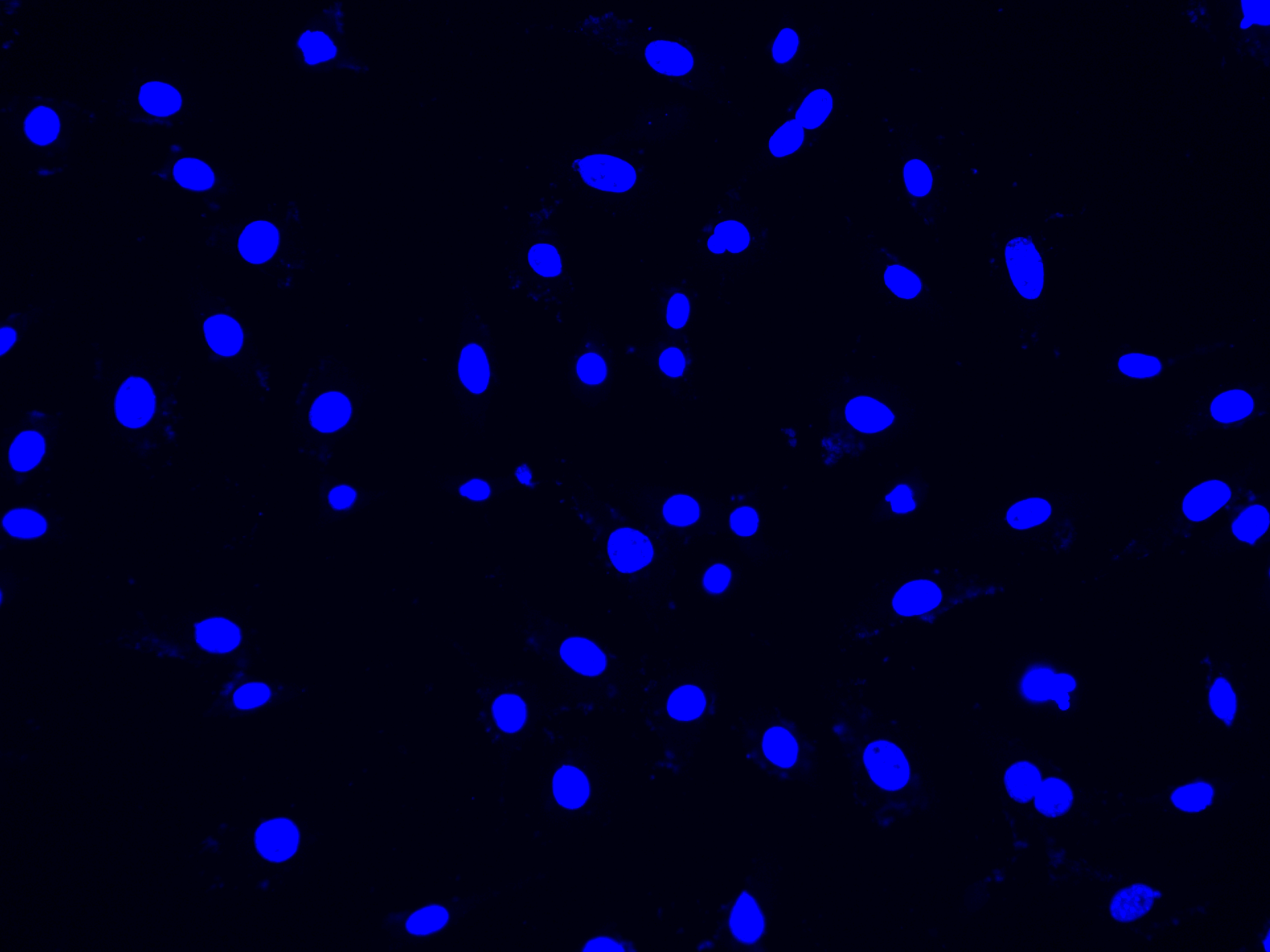

Supplement: S1 File — (ZIP) [file pone.0333897.s001.zip › Raw data/Figure 4/IF/Control/LCB RUNX2_C2_ch00_SV.tif]

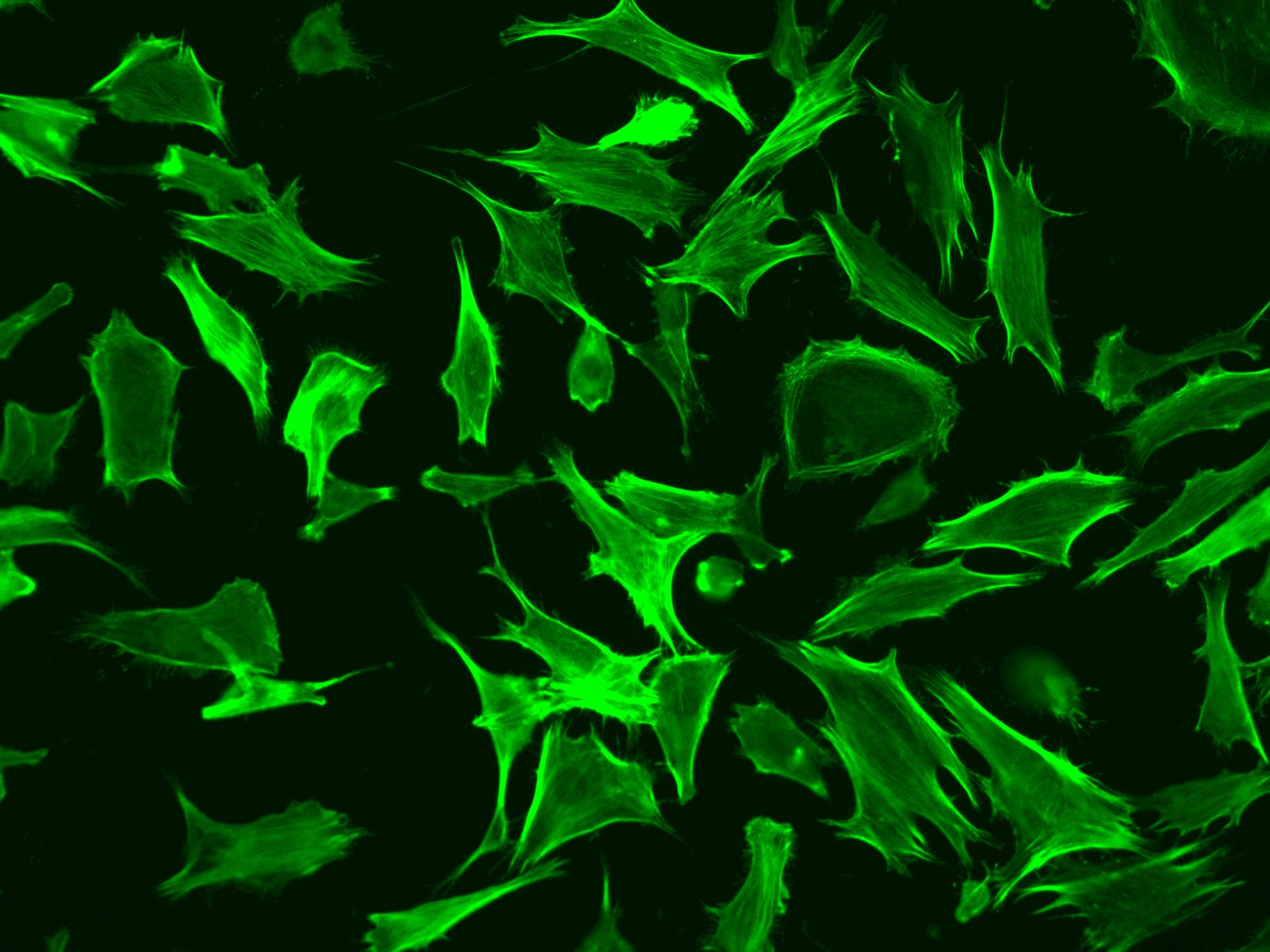

Supplement: S1 File — (ZIP) [file pone.0333897.s001.zip › Raw data/Figure 4/IF/Control/LCB RUNX2_C2_ch01_SV.tif]

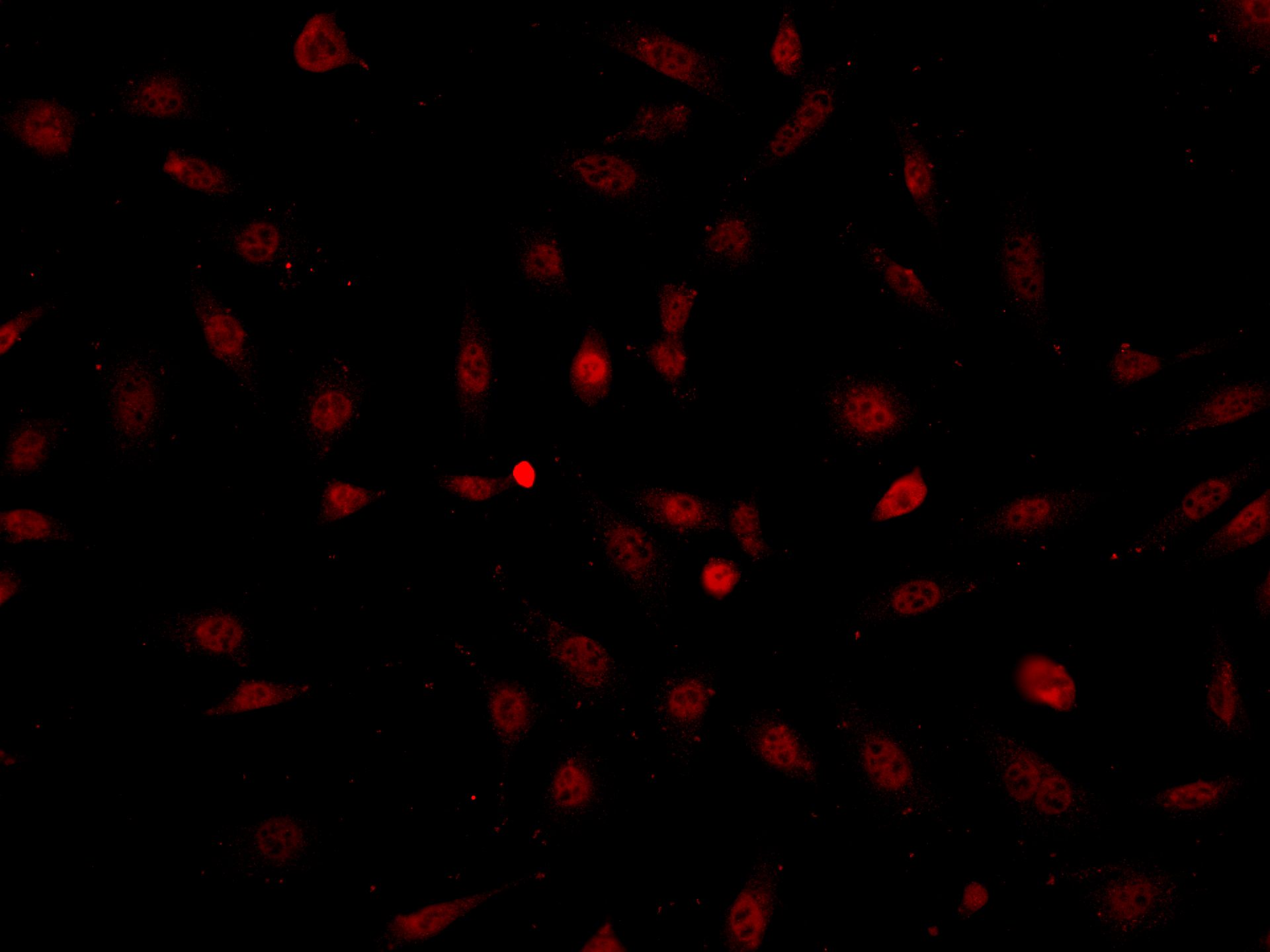

Supplement: S1 File — (ZIP) [file pone.0333897.s001.zip › Raw data/Figure 4/IF/Control/LCB RUNX2_C2_ch02_SV.tif]

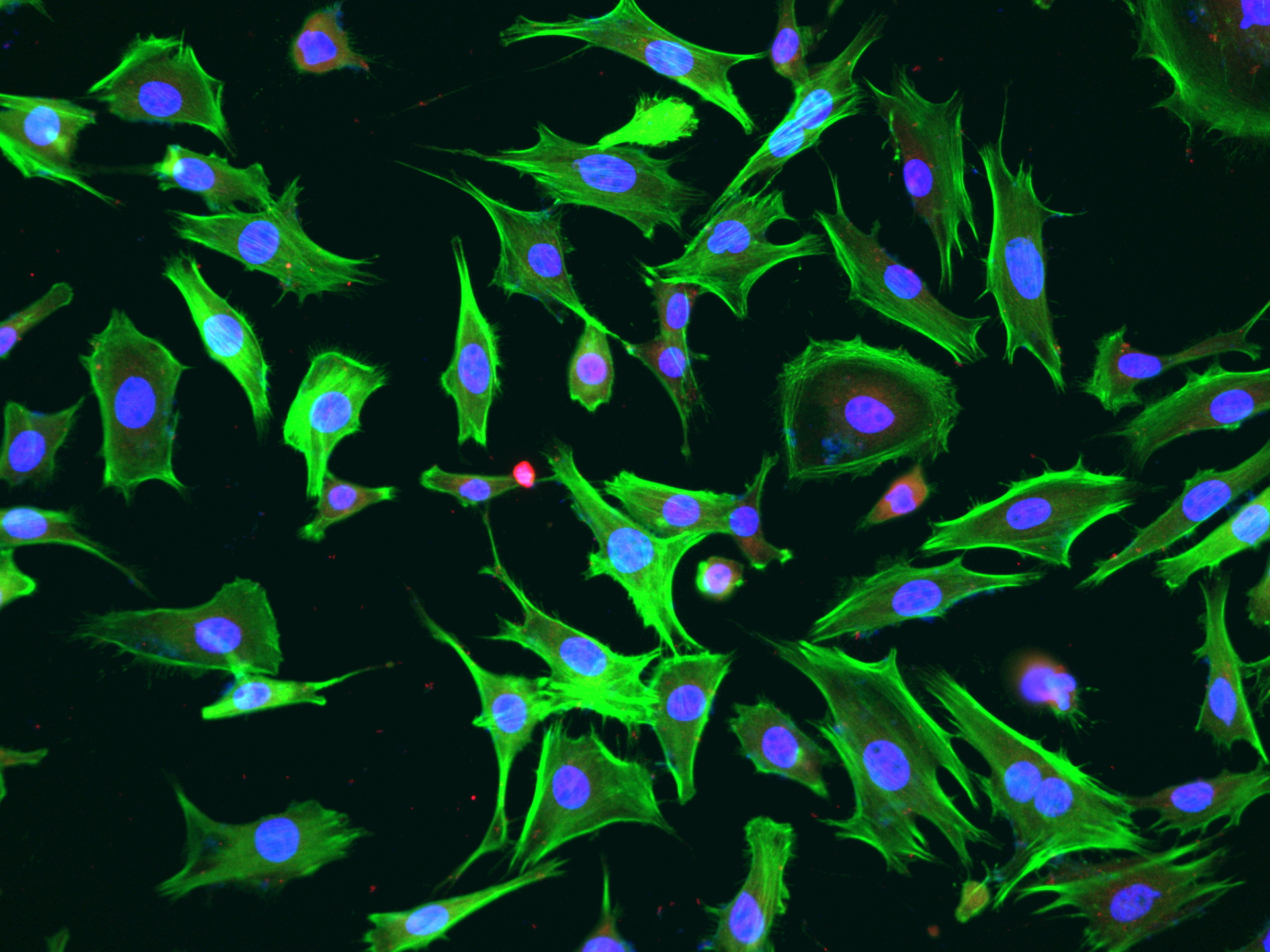

Supplement: S1 File — (ZIP) [file pone.0333897.s001.zip › Raw data/Figure 4/IF/Control/LCB RUNX2_C2_overlay.tif]

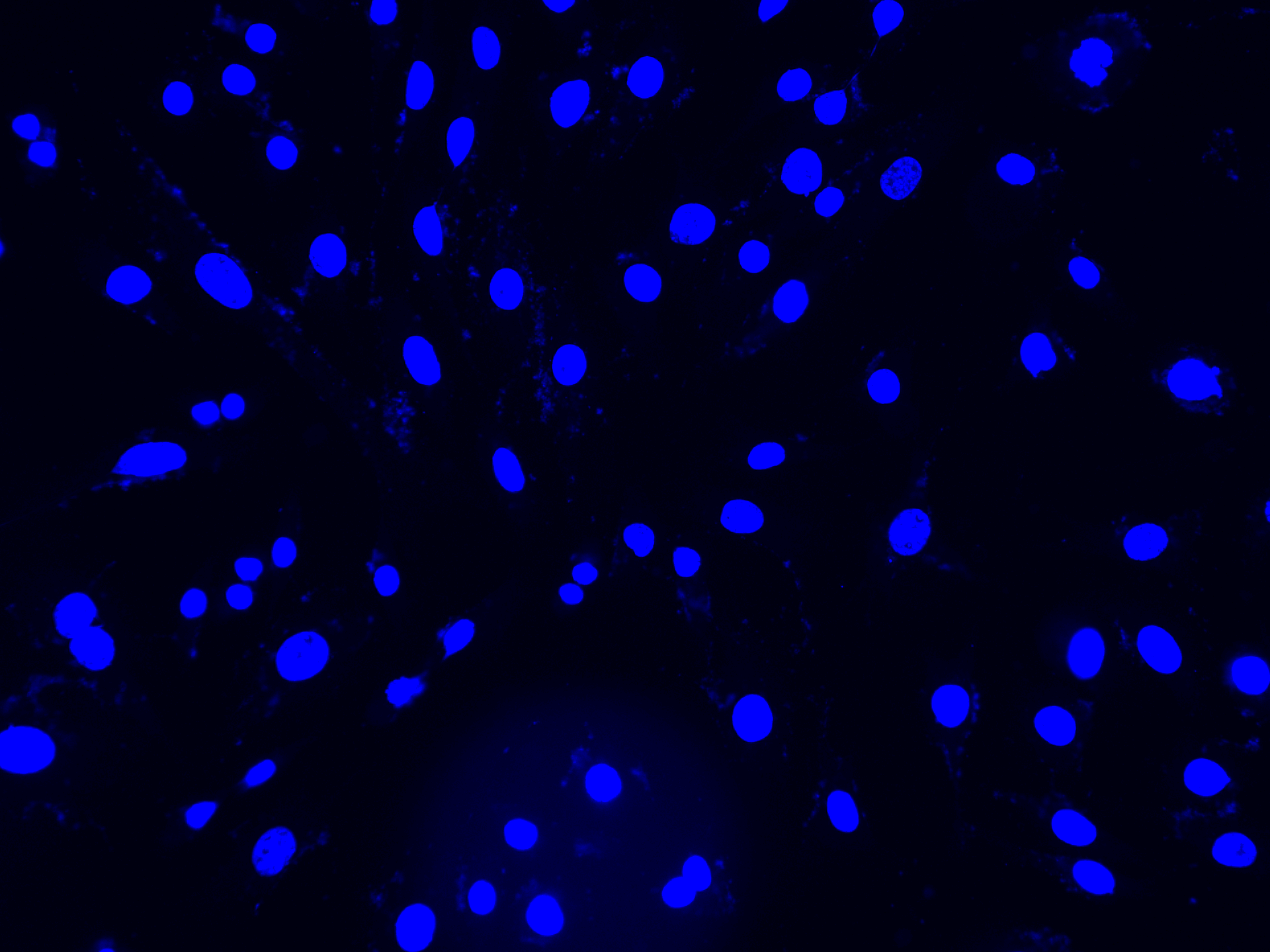

Supplement: S1 File — (ZIP) [file pone.0333897.s001.zip › Raw data/Figure 4/IF/EXOs/LCB RUNX2_B2_ch00_SV.tif]

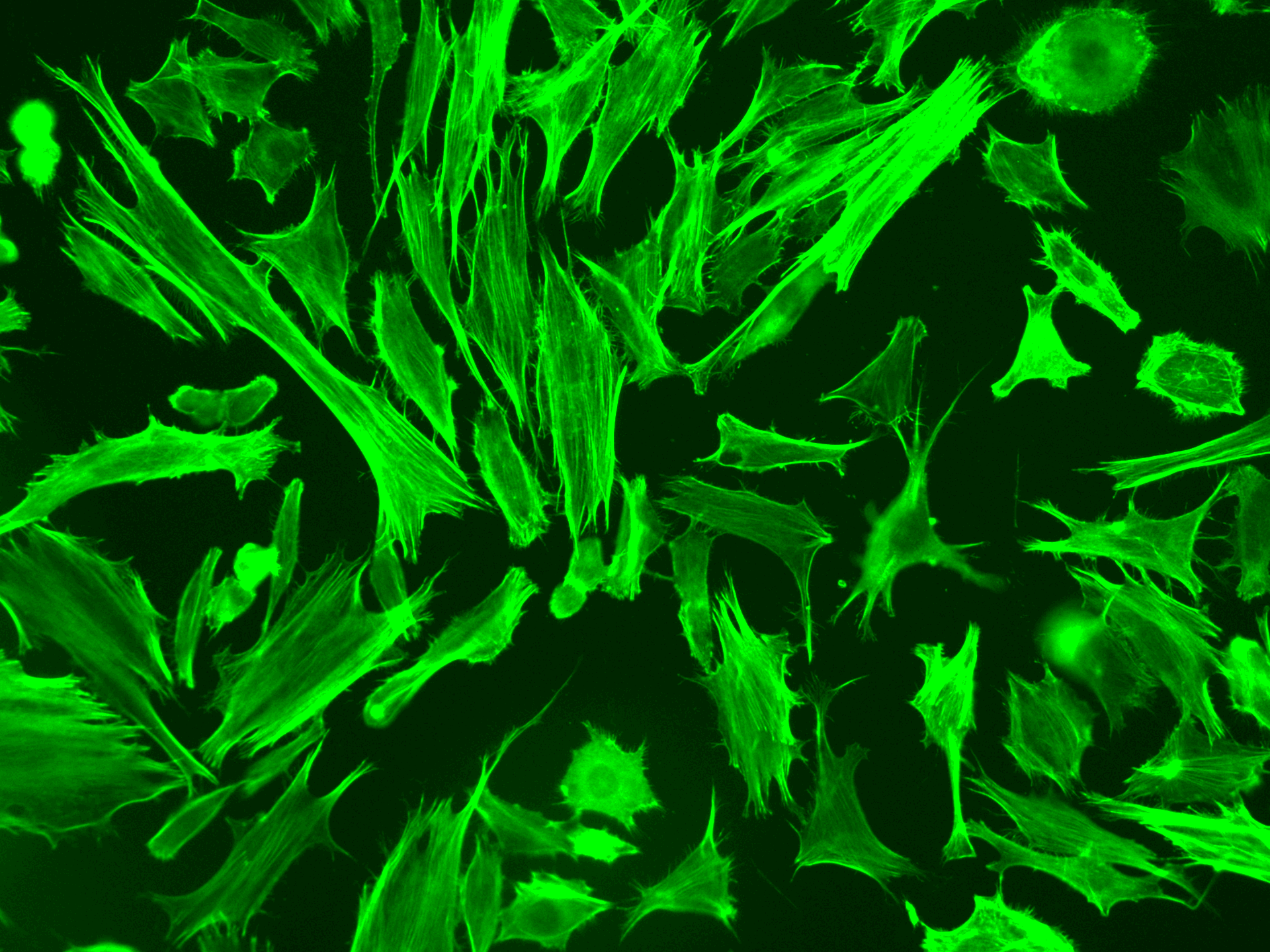

Supplement: S1 File — (ZIP) [file pone.0333897.s001.zip › Raw data/Figure 4/IF/EXOs/LCB RUNX2_B2_ch01_SV.tif]

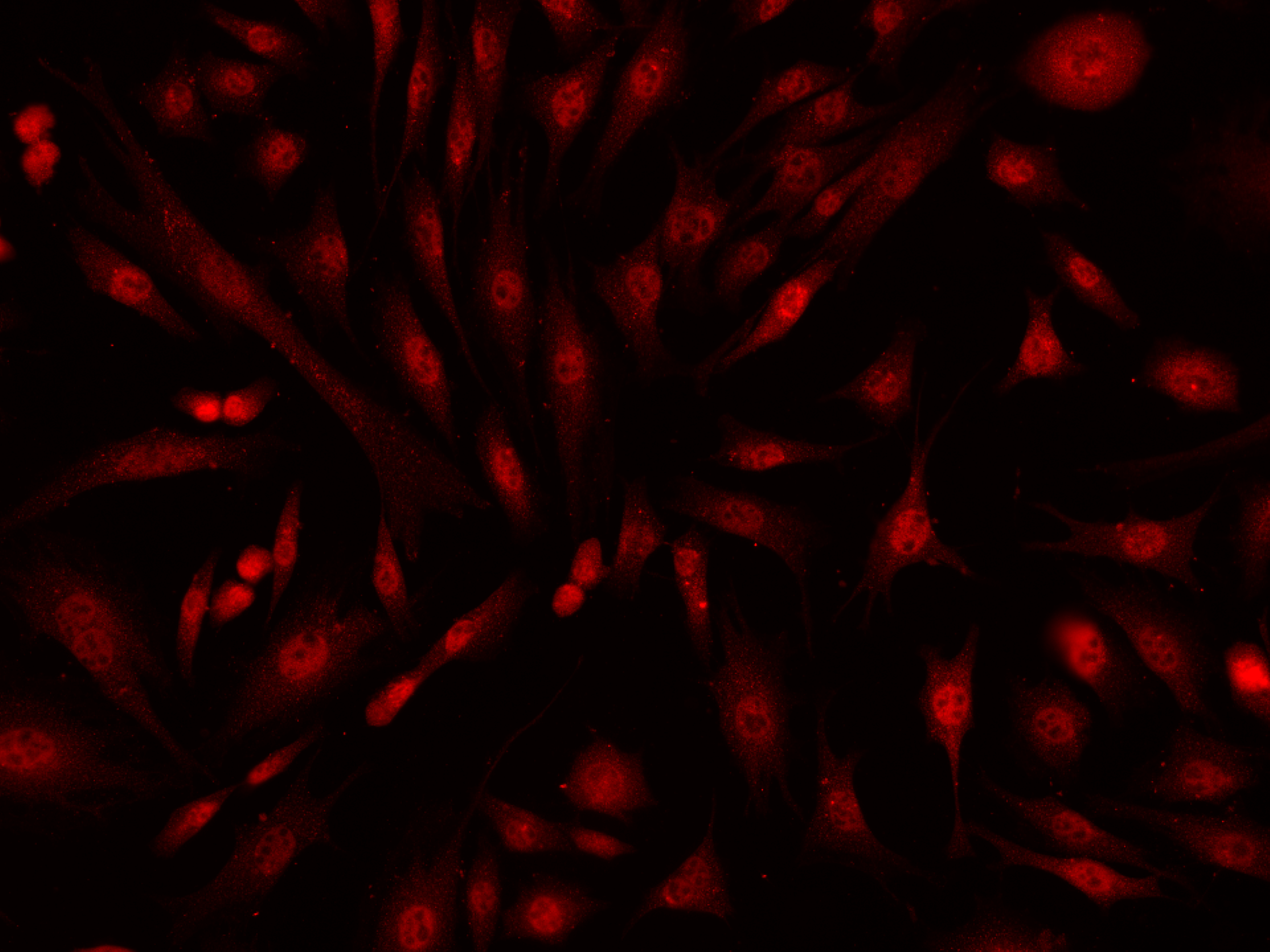

Supplement: S1 File — (ZIP) [file pone.0333897.s001.zip › Raw data/Figure 4/IF/EXOs/LCB RUNX2_B2_ch02_SV.tif]

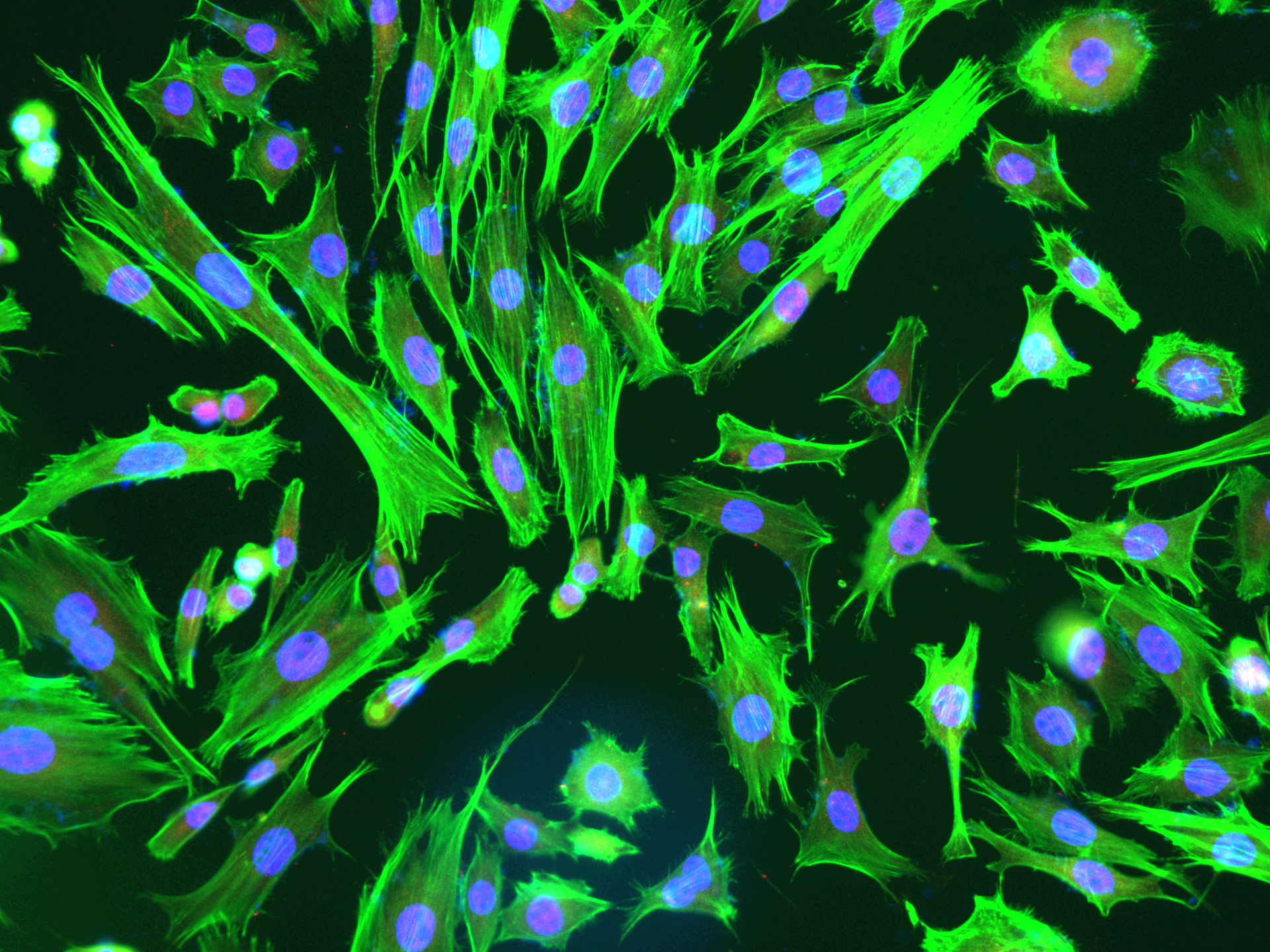

Supplement: S1 File — (ZIP) [file pone.0333897.s001.zip › Raw data/Figure 4/IF/EXOs/LCB RUNX2_B2_overlay.tif]

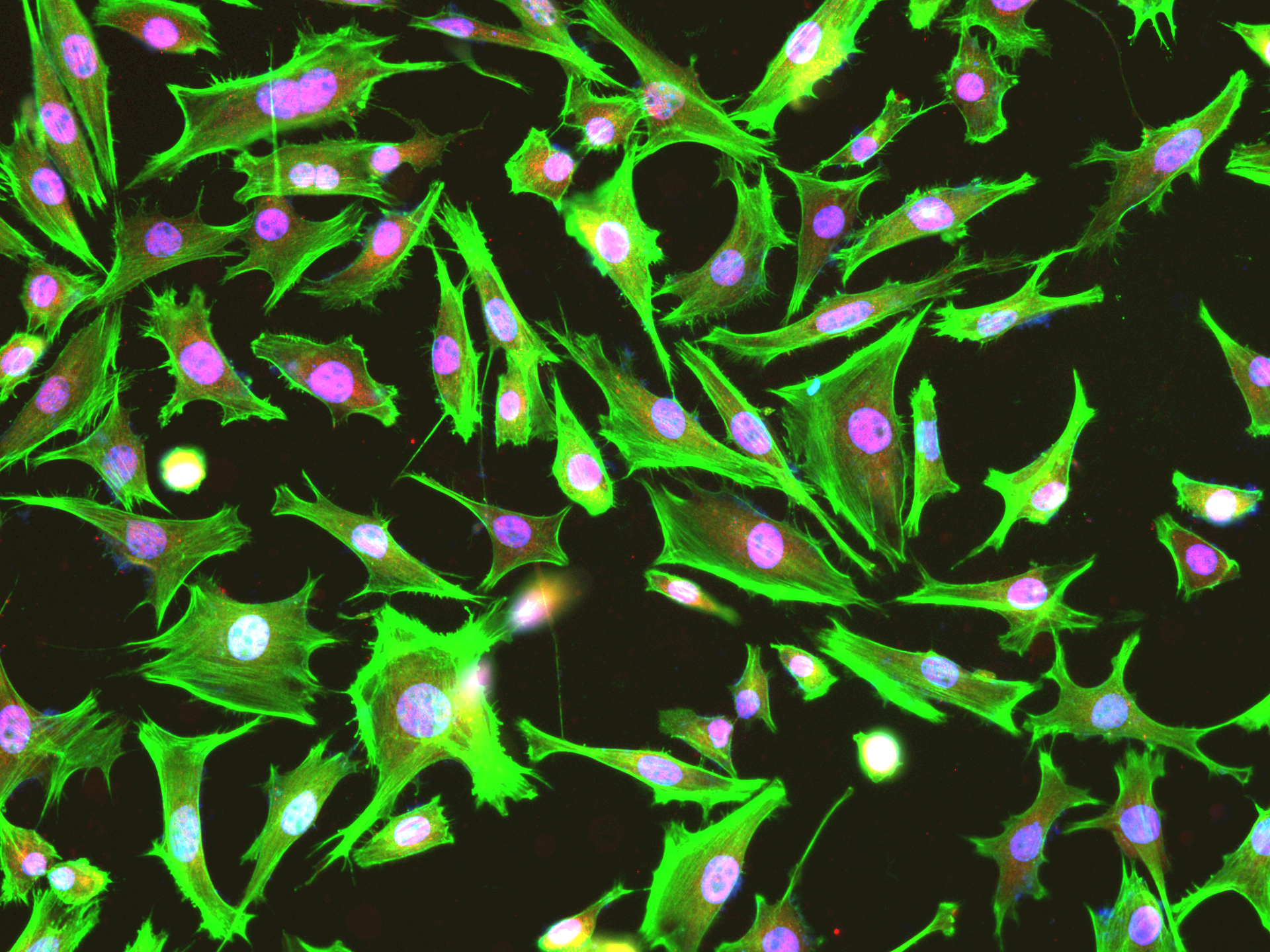

Supplement: S1 File — (ZIP) [file pone.0333897.s001.zip › Raw data/Figure 4/IF/S-EXOs/12RGB.tif]

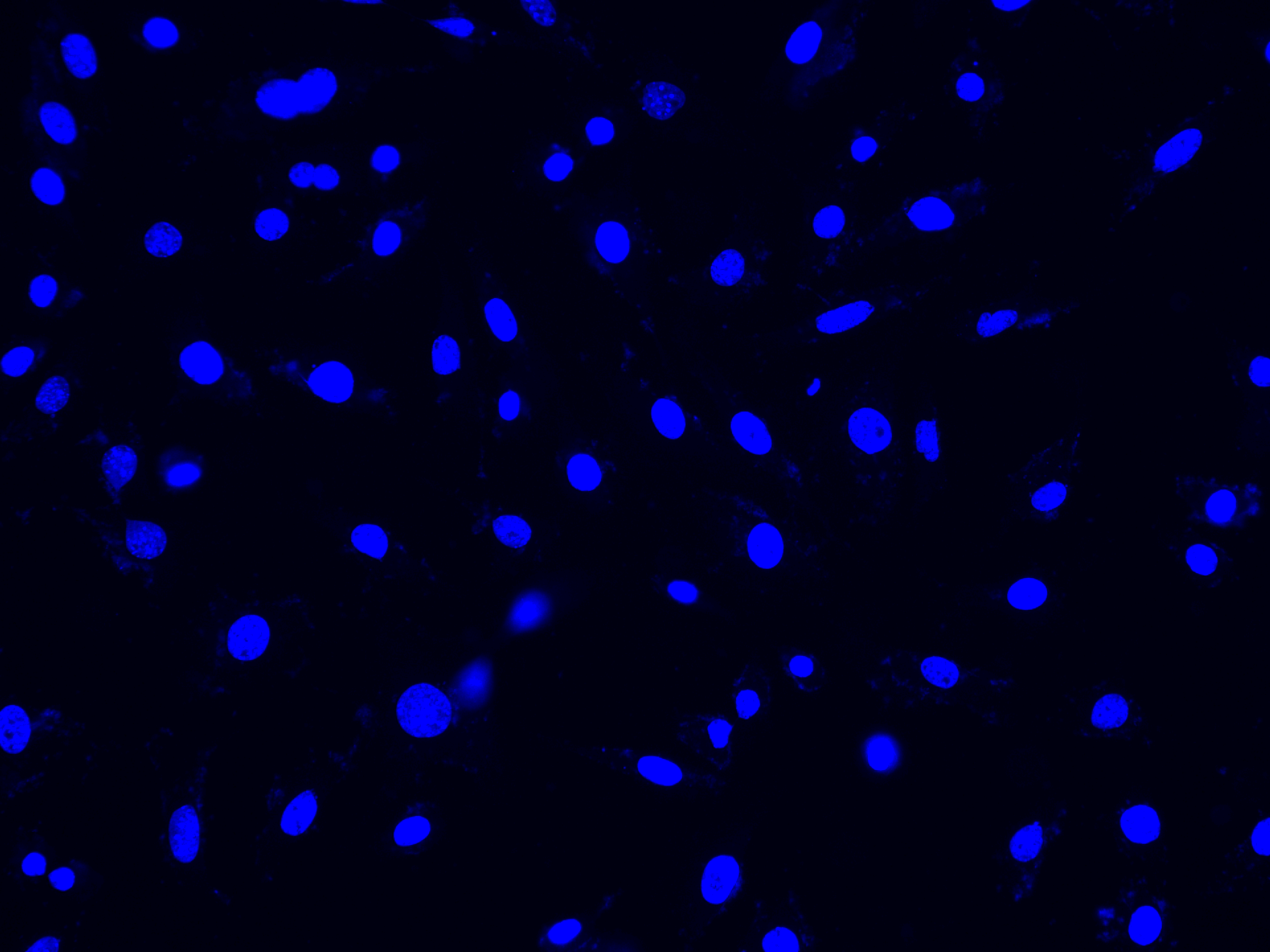

Supplement: S1 File — (ZIP) [file pone.0333897.s001.zip › Raw data/Figure 4/IF/S-EXOs/LCB RUNX2_Series031_ch00_SV.tif]

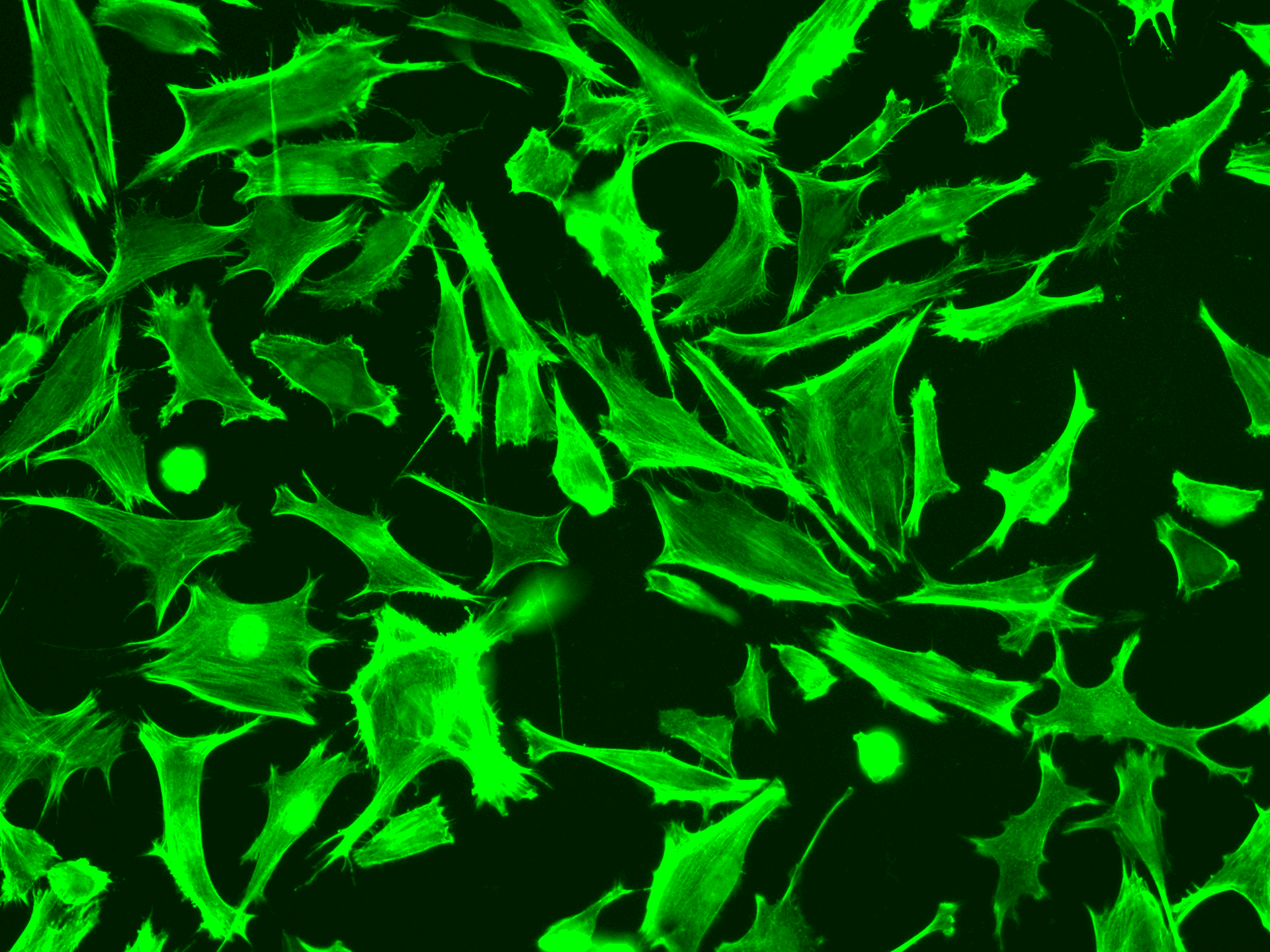

Supplement: S1 File — (ZIP) [file pone.0333897.s001.zip › Raw data/Figure 4/IF/S-EXOs/LCB RUNX2_Series031_ch01_SV.tif]

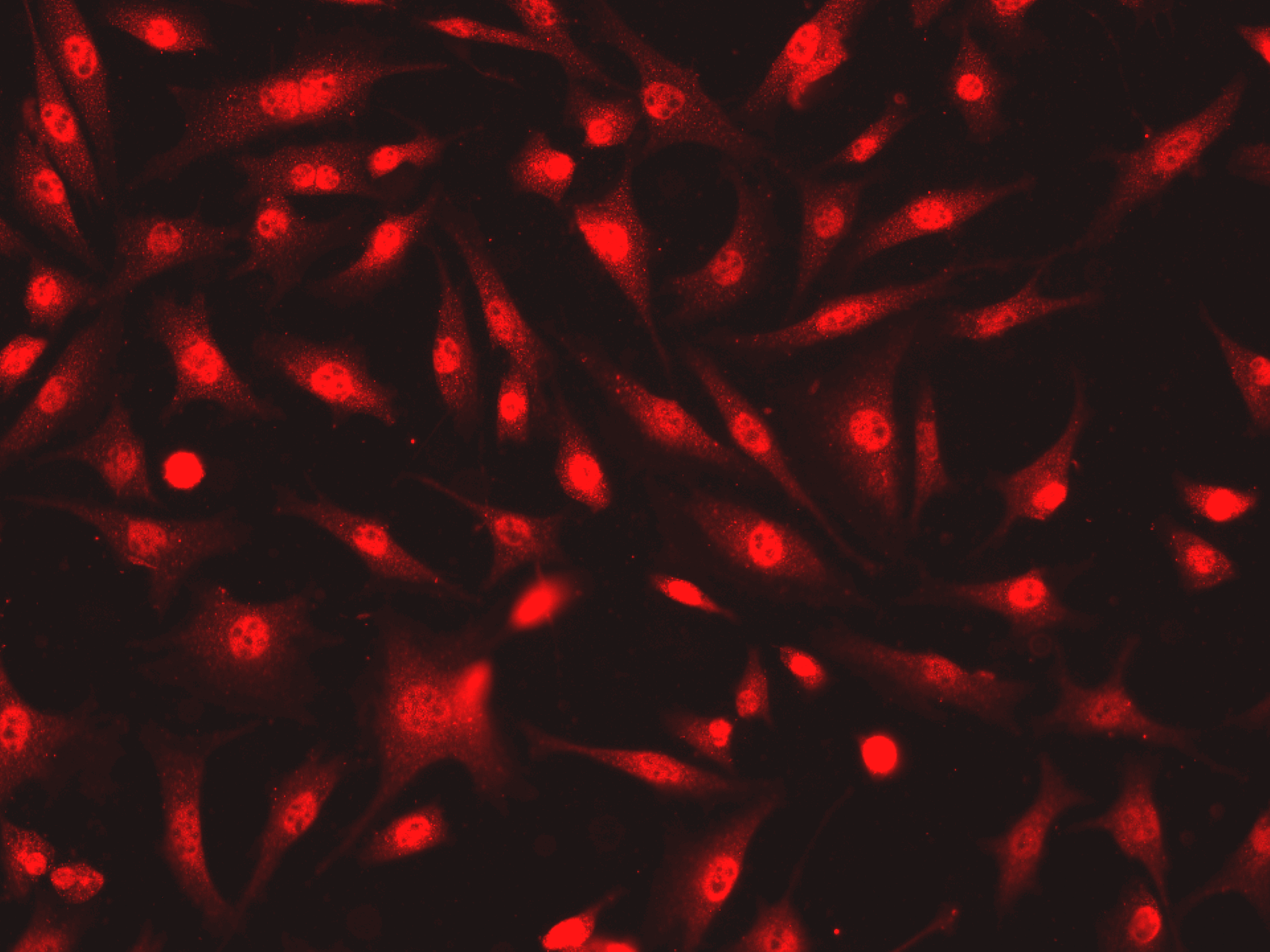

Supplement: S1 File — (ZIP) [file pone.0333897.s001.zip › Raw data/Figure 4/IF/S-EXOs/LCB RUNX2_Series031_ch02_SV.tif]

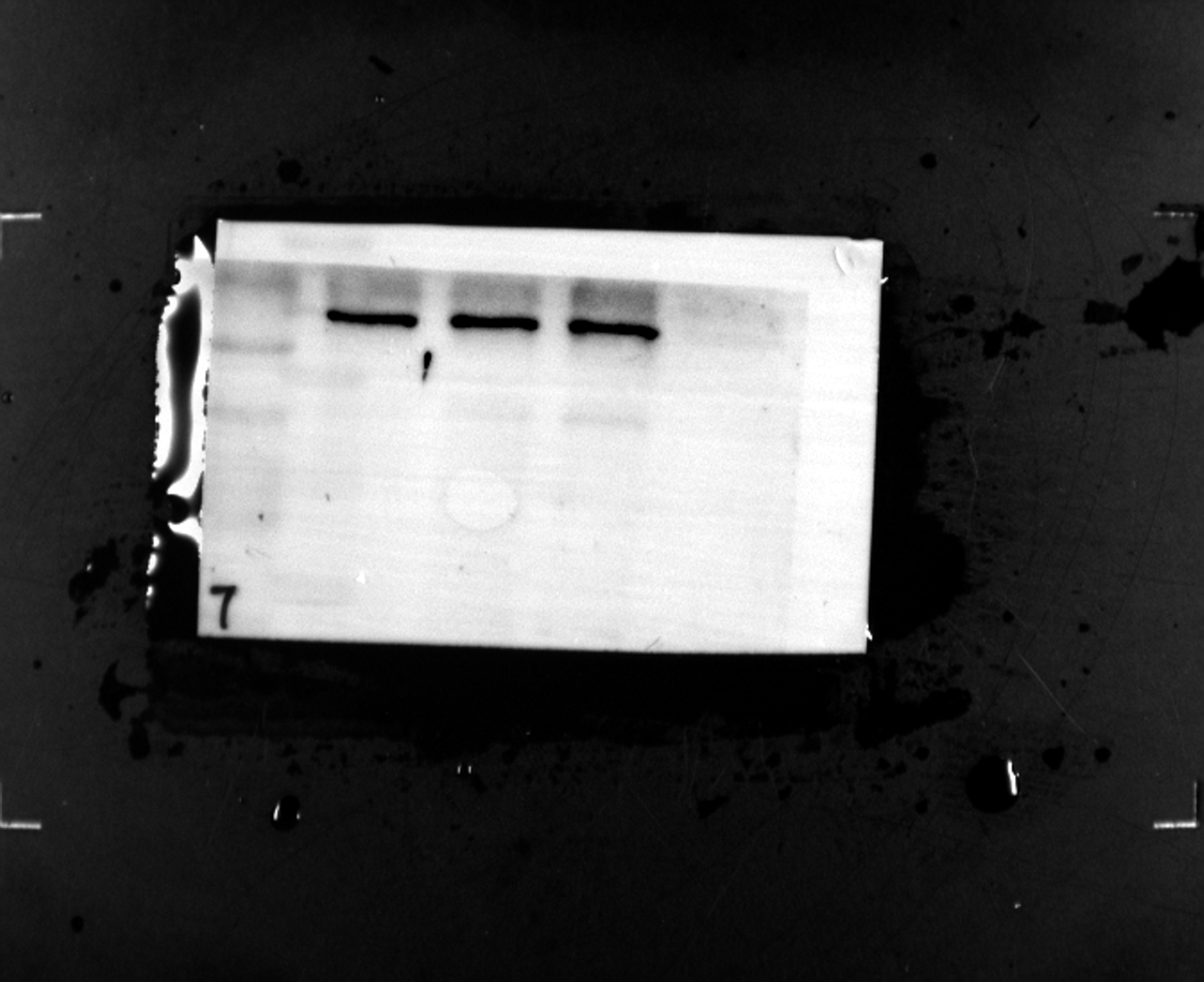

Supplement: S1 File — (ZIP) [file pone.0333897.s001.zip › Raw data/Figure 4/RUNX2/Actin-m.Tif]

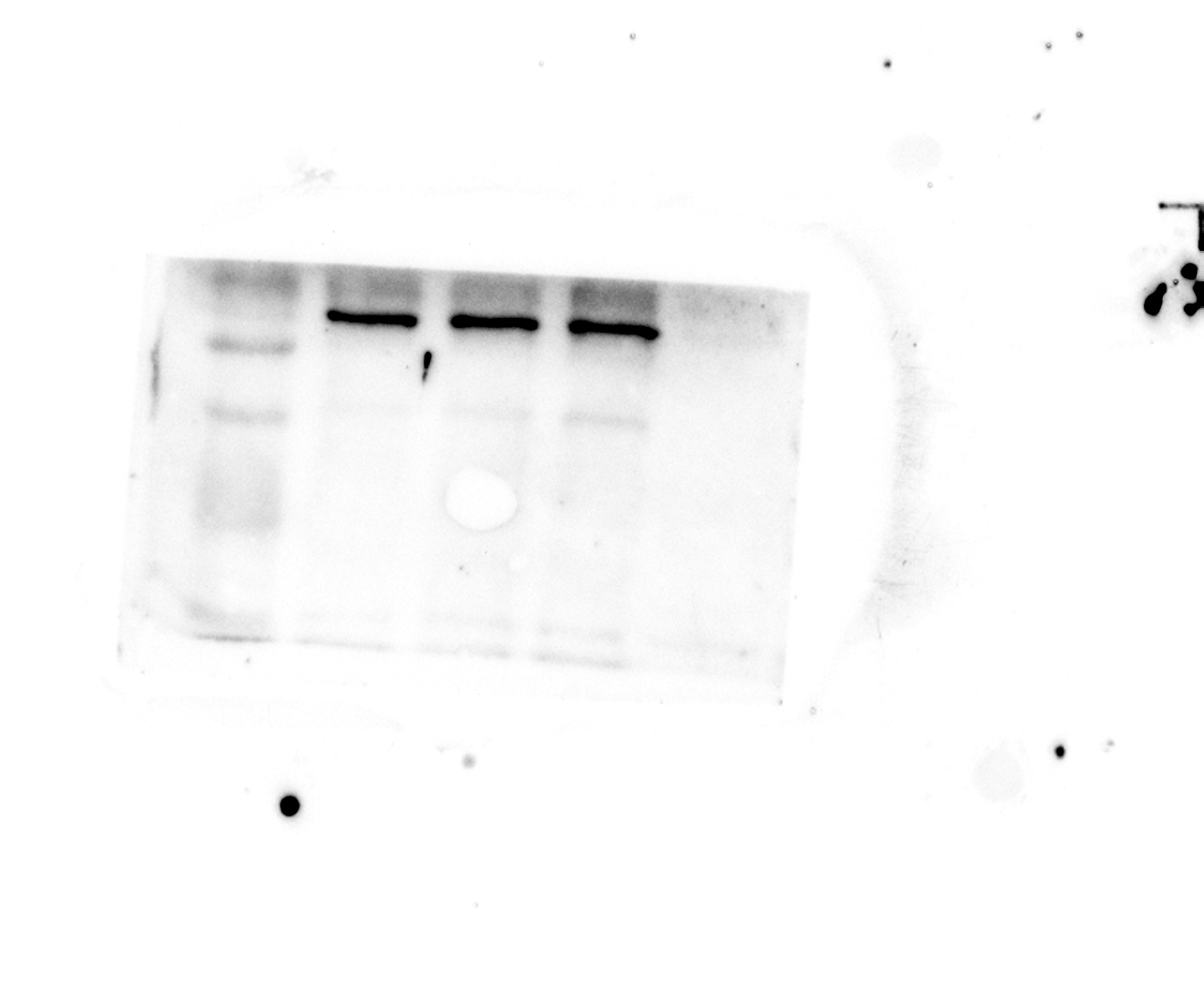

Supplement: S1 File — (ZIP) [file pone.0333897.s001.zip › Raw data/Figure 4/RUNX2/Actin.Tif]

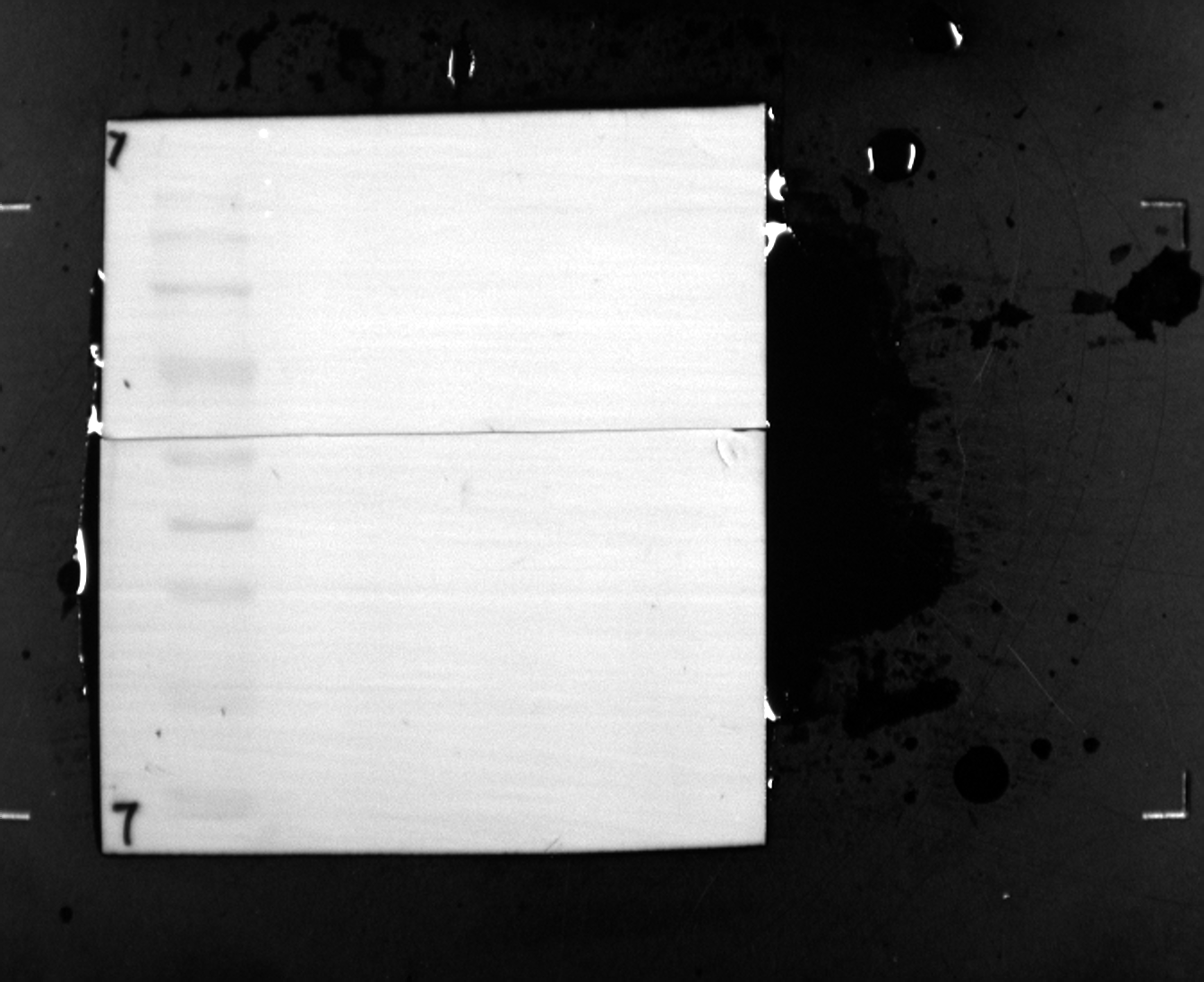

Supplement: S1 File — (ZIP) [file pone.0333897.s001.zip › Raw data/Figure 4/RUNX2/merge.Tif]

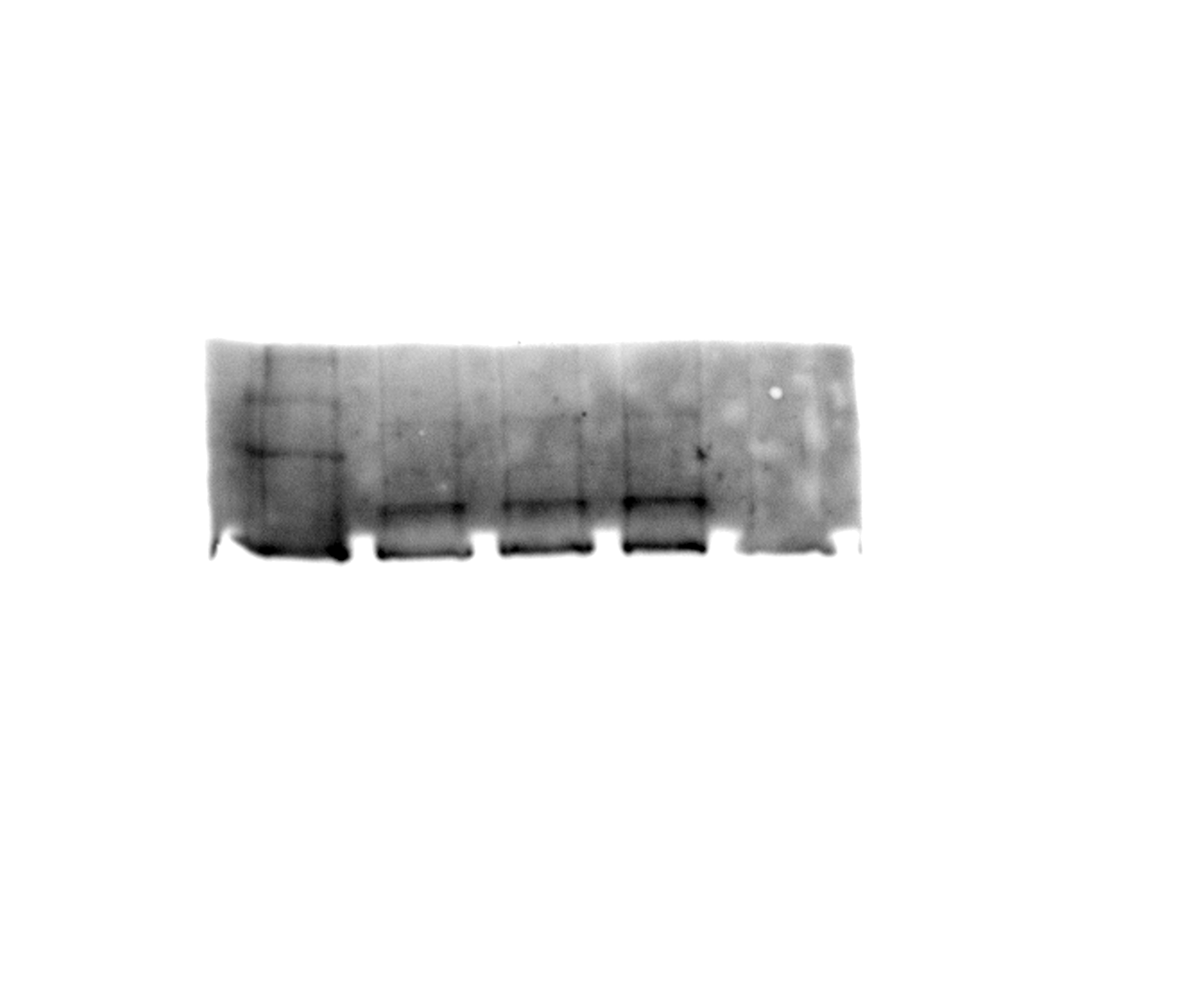

Supplement: S1 File — (ZIP) [file pone.0333897.s001.zip › Raw data/Figure 4/RUNX2/runx2.Tif]

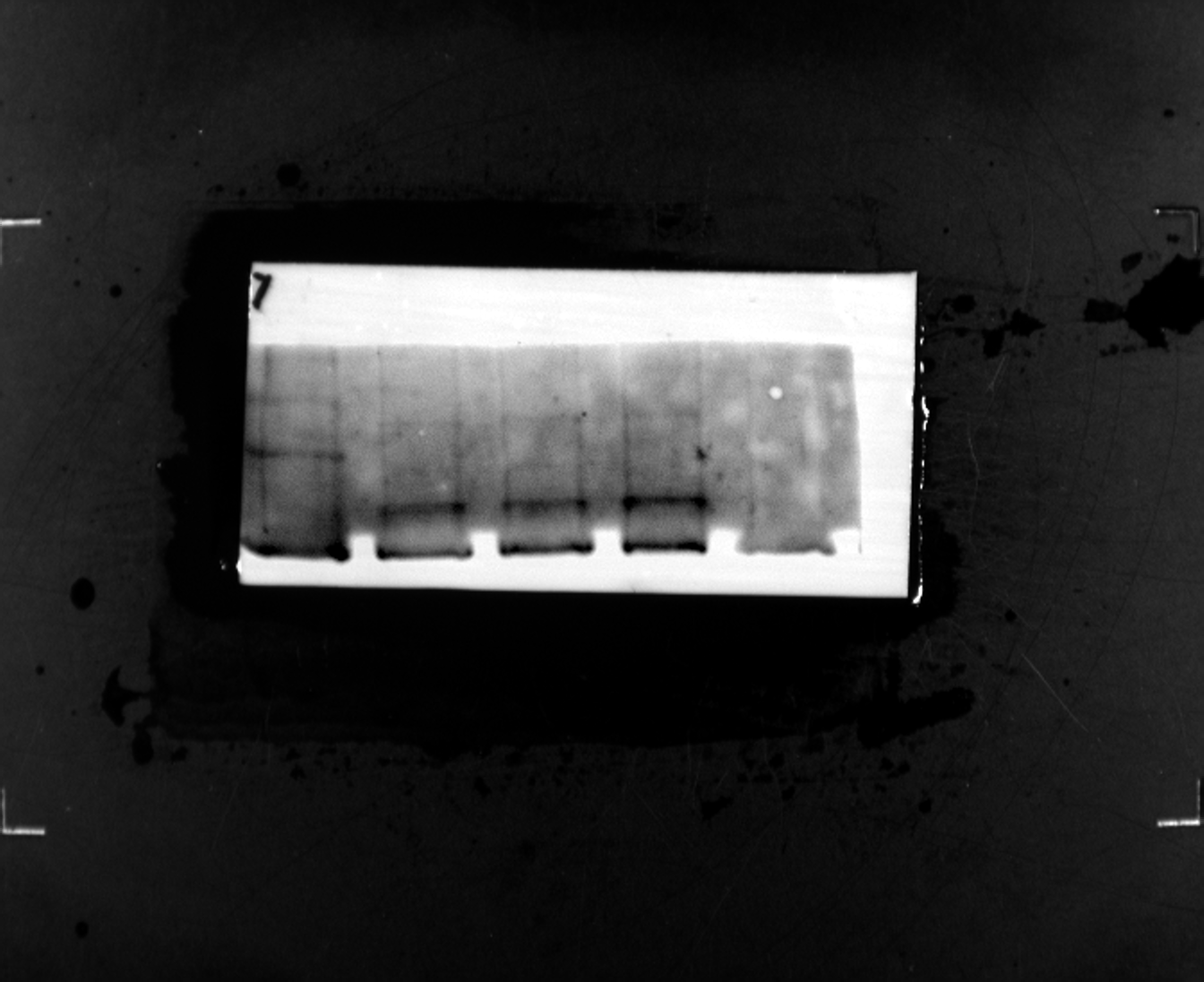

Supplement: S1 File — (ZIP) [file pone.0333897.s001.zip › Raw data/Figure 4/RUNX2/runx2M.Tif]

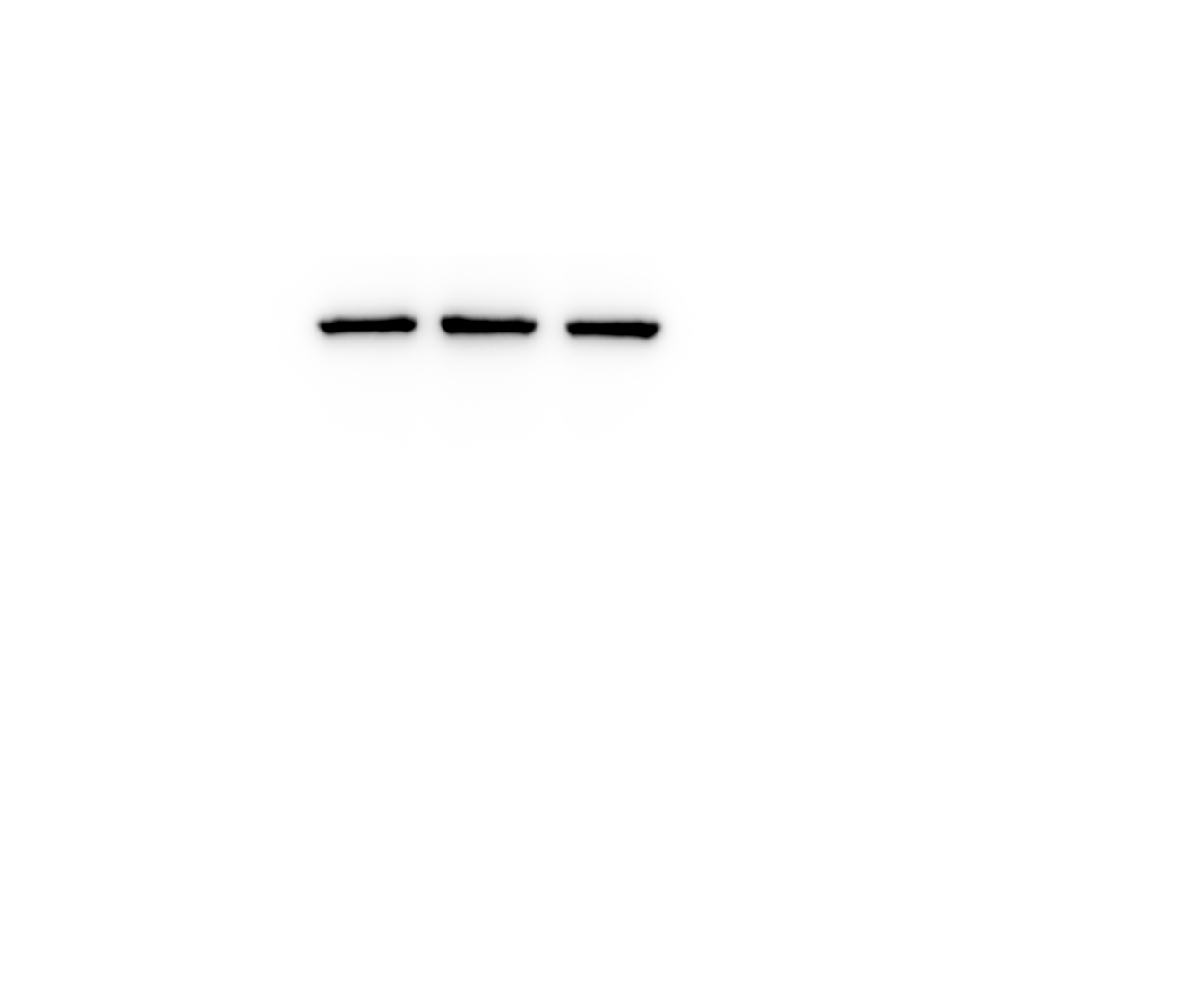

Supplement: S1 File — (ZIP) [file pone.0333897.s001.zip › Raw data/Figure 4/Sema3a/actin.Tif]

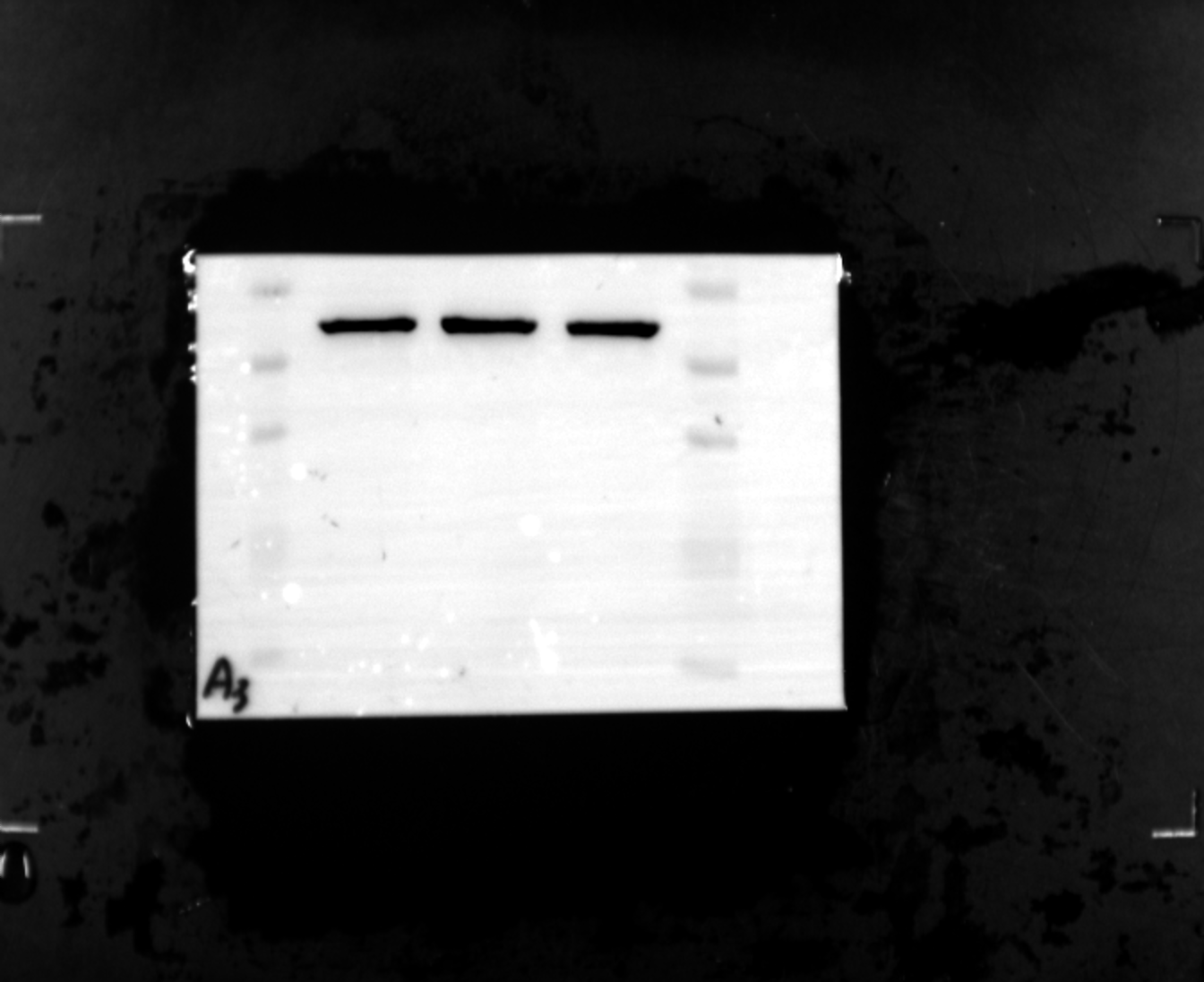

Supplement: S1 File — (ZIP) [file pone.0333897.s001.zip › Raw data/Figure 4/Sema3a/actin1.Tif]

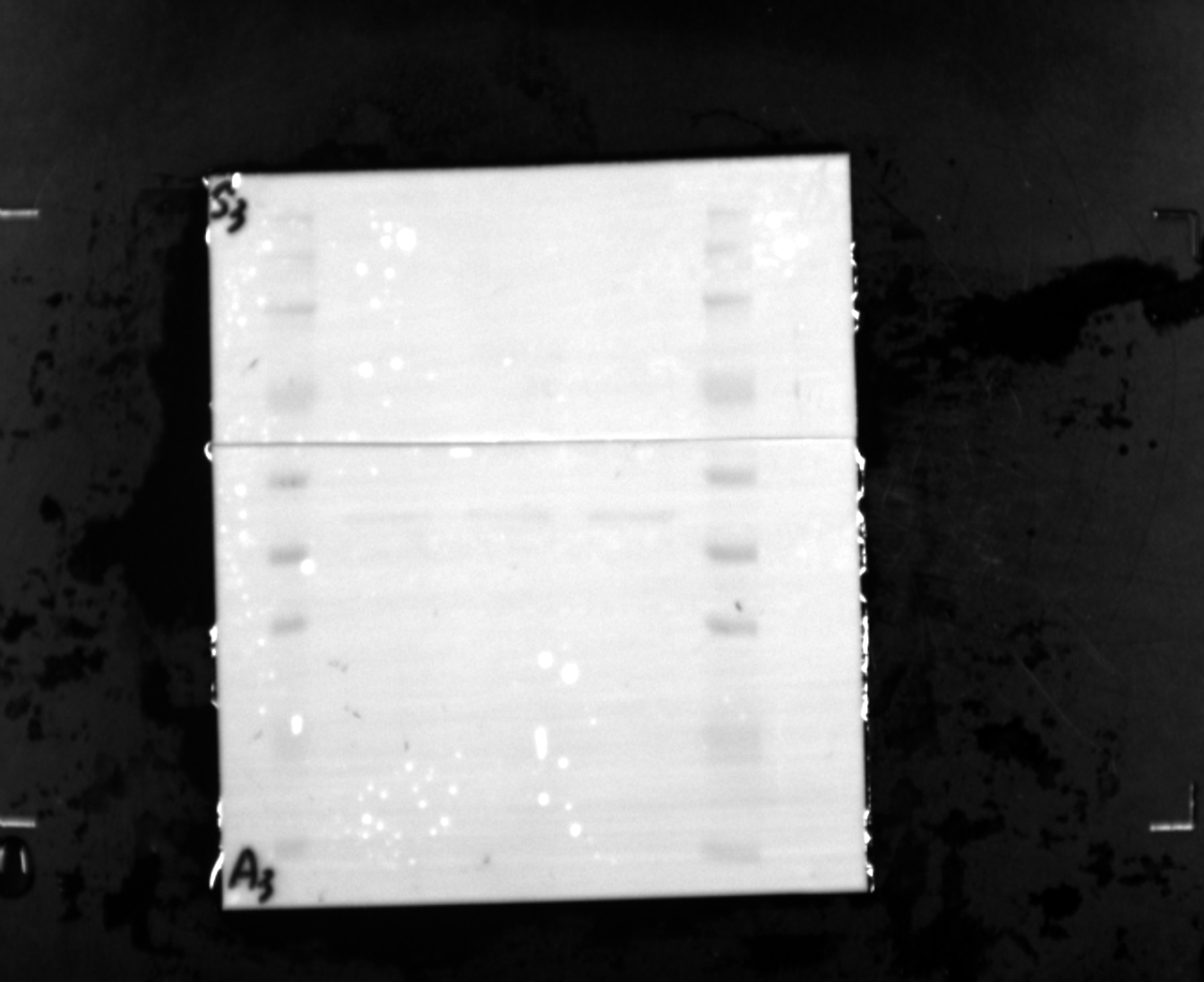

Supplement: S1 File — (ZIP) [file pone.0333897.s001.zip › Raw data/Figure 4/Sema3a/merge.Tif]

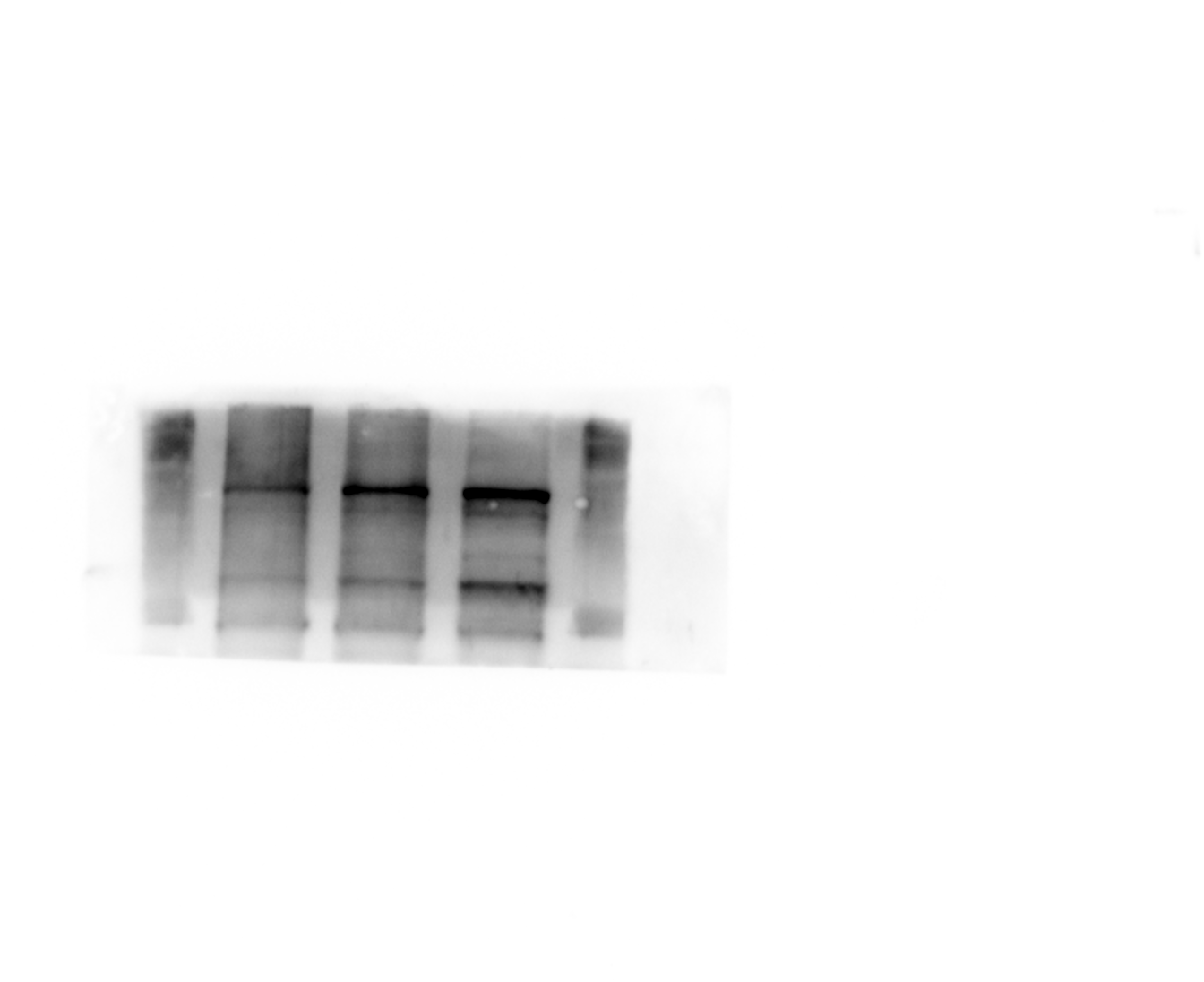

Supplement: S1 File — (ZIP) [file pone.0333897.s001.zip › Raw data/Figure 4/Sema3a/sema3a.Tif]

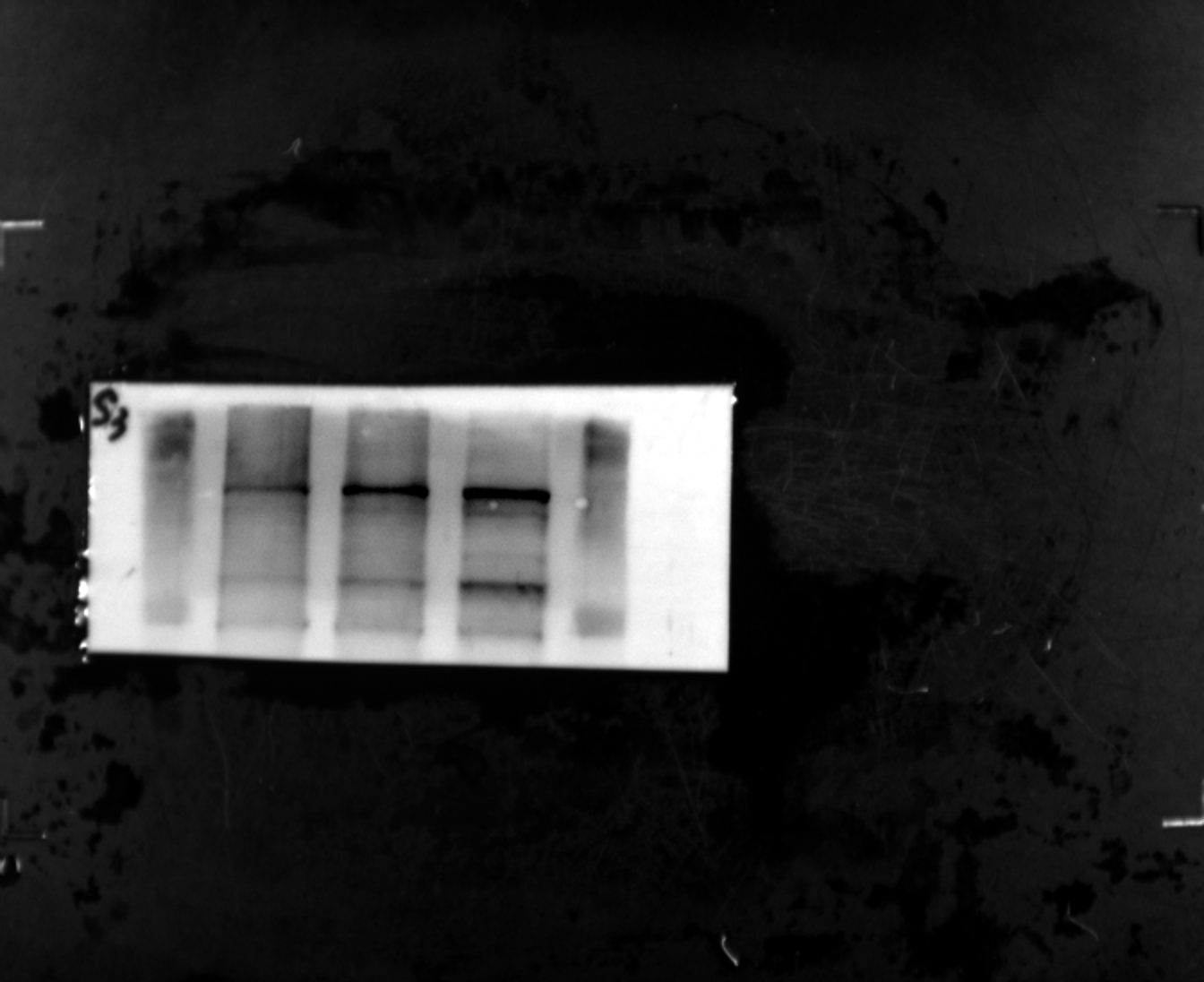

Supplement: S1 File — (ZIP) [file pone.0333897.s001.zip › Raw data/Figure 4/Sema3a/sema3a1.Tif]

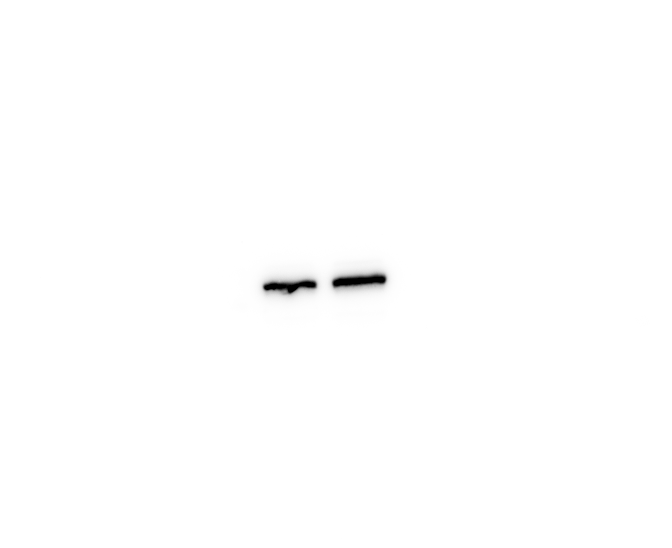

Supplement: S1 File — (ZIP) [file pone.0333897.s001.zip › Raw data/Figure 5/OPN/ACTIN.Tif]

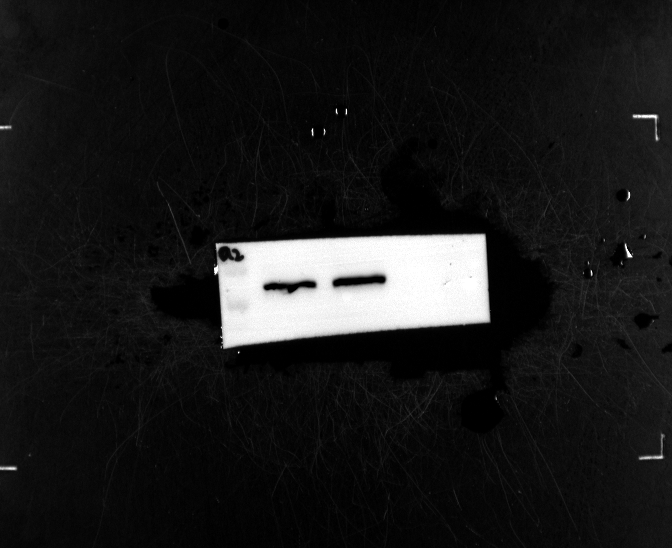

Supplement: S1 File — (ZIP) [file pone.0333897.s001.zip › Raw data/Figure 5/OPN/ACTIN1.Tif]

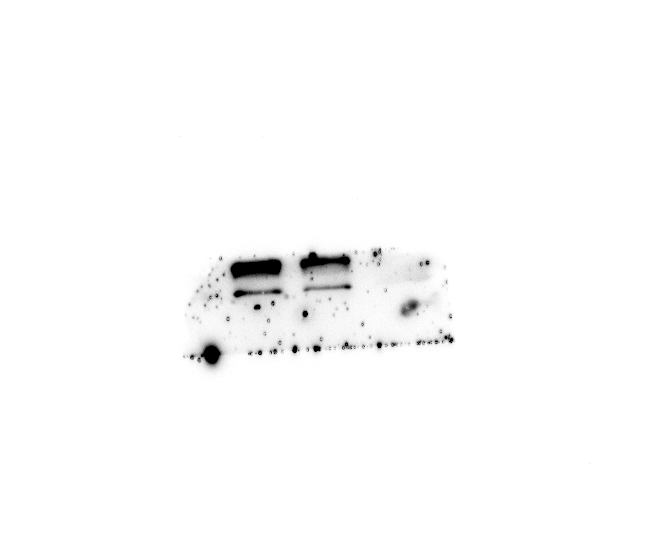

Supplement: S1 File — (ZIP) [file pone.0333897.s001.zip › Raw data/Figure 5/OPN/OPN.Tif]

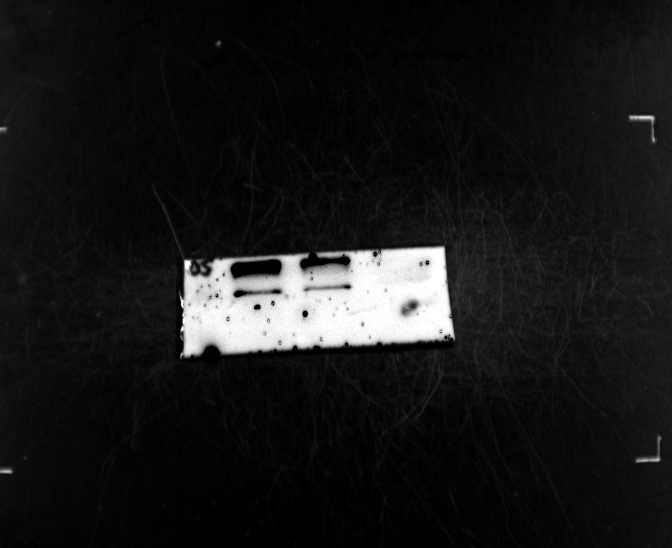

Supplement: S1 File — (ZIP) [file pone.0333897.s001.zip › Raw data/Figure 5/OPN/OPN1.Tif]

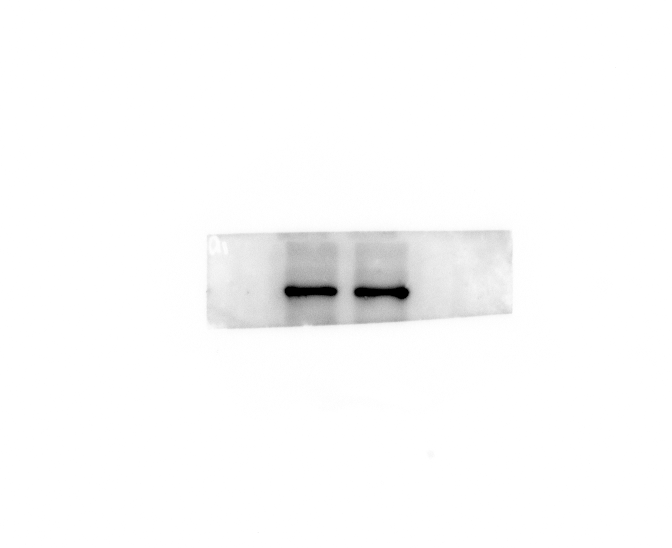

Supplement: S1 File — (ZIP) [file pone.0333897.s001.zip › Raw data/Figure 5/runx2/ACTIN.Tif]

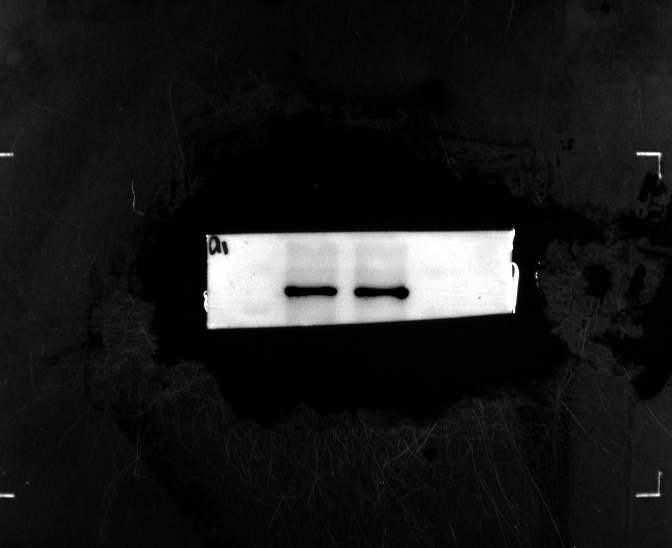

Supplement: S1 File — (ZIP) [file pone.0333897.s001.zip › Raw data/Figure 5/runx2/ACTIN1.Tif]

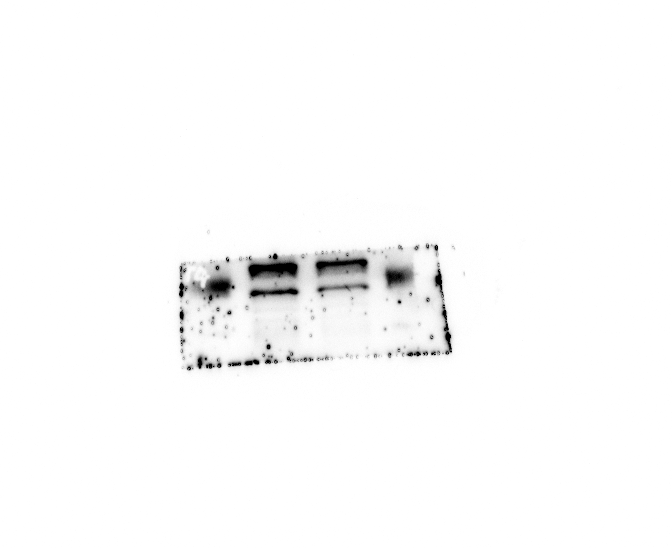

Supplement: S1 File — (ZIP) [file pone.0333897.s001.zip › Raw data/Figure 5/runx2/RUNX2.Tif]

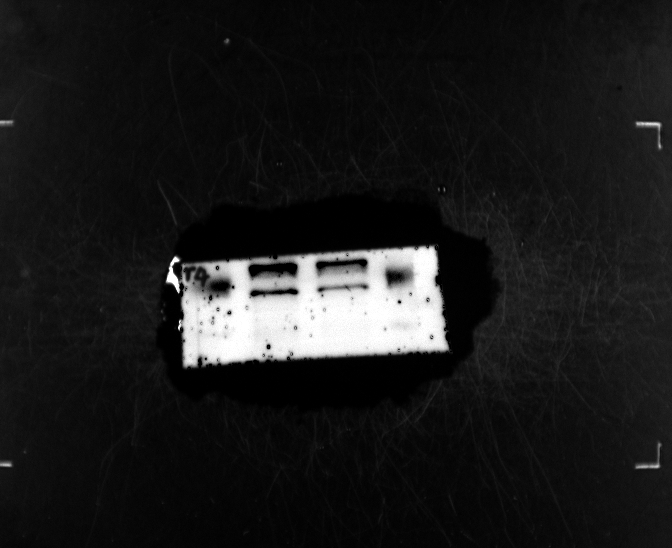

Supplement: S1 File — (ZIP) [file pone.0333897.s001.zip › Raw data/Figure 5/runx2/RUNX21.Tif]

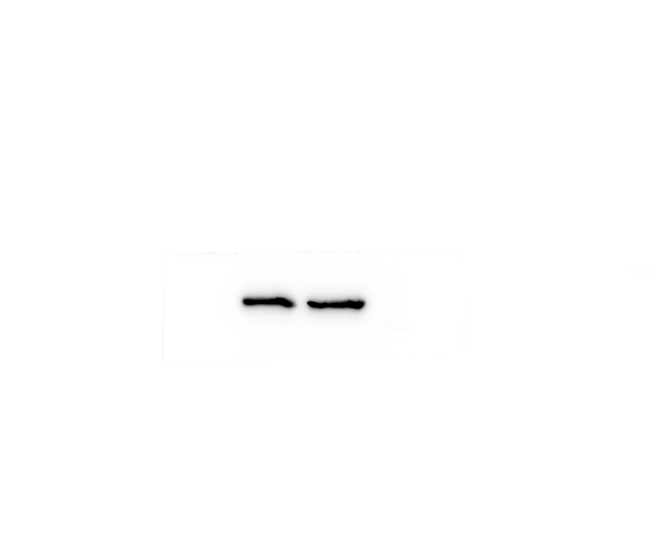

Supplement: S1 File — (ZIP) [file pone.0333897.s001.zip › Raw data/Figure 5/sema3A/actin.Tif]

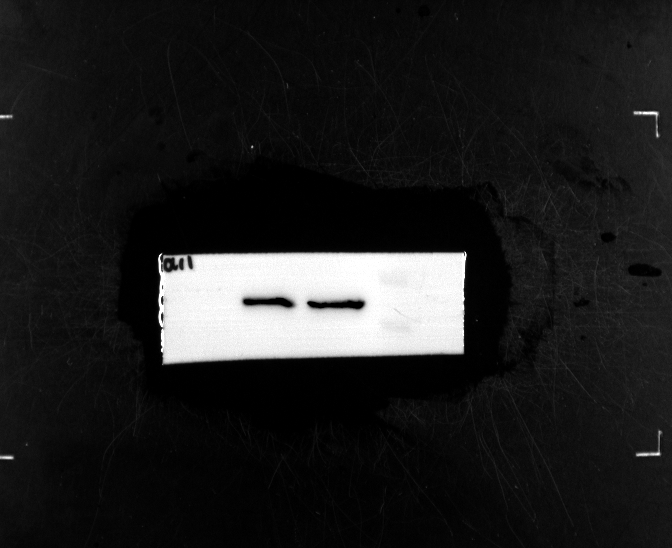

Supplement: S1 File — (ZIP) [file pone.0333897.s001.zip › Raw data/Figure 5/sema3A/actin1.Tif]

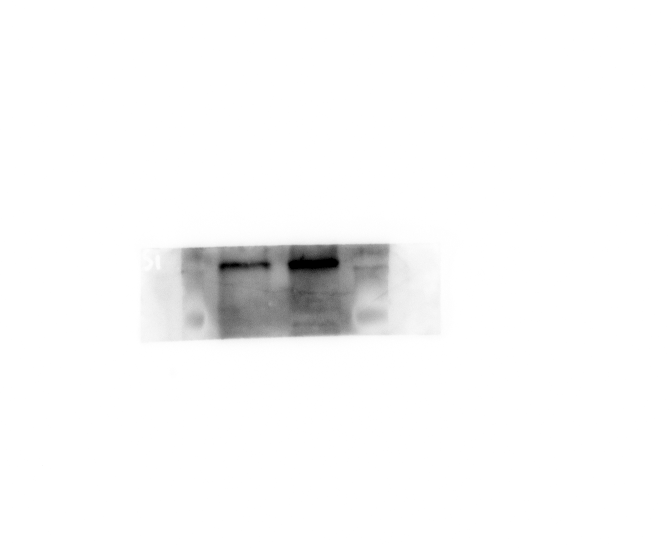

Supplement: S1 File — (ZIP) [file pone.0333897.s001.zip › Raw data/Figure 5/sema3A/SEMA3A.Tif]

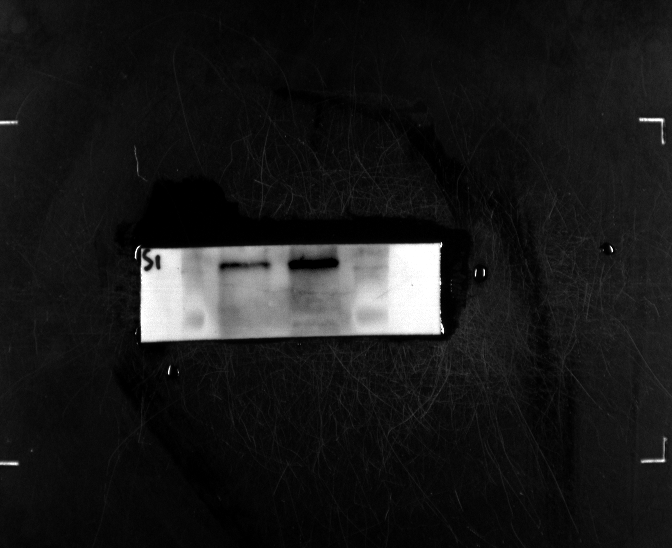

Supplement: S1 File — (ZIP) [file pone.0333897.s001.zip › Raw data/Figure 5/sema3A/SEMA3A1.Tif]

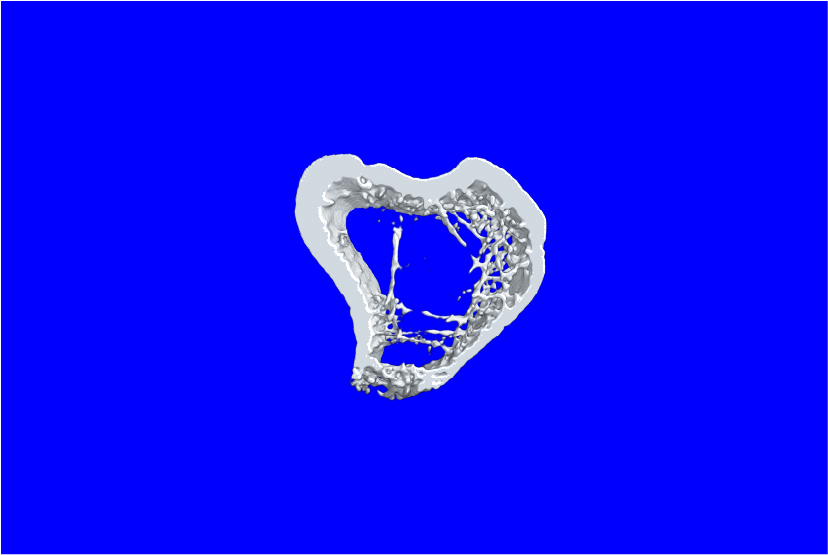

Supplement: S1 File — (ZIP) [file pone.0333897.s001.zip › Raw data/Figure6/Image/EXOs@BMSC.jpg]

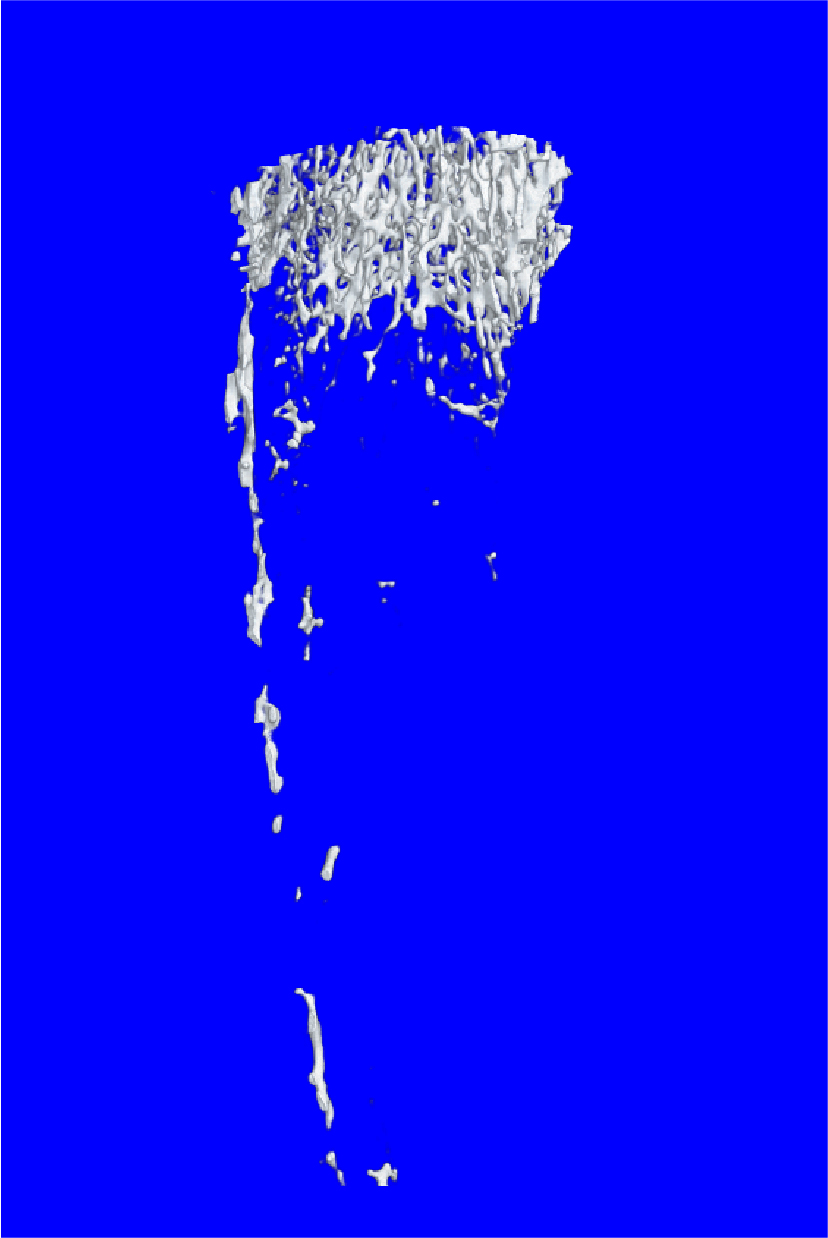

Supplement: S1 File — (ZIP) [file pone.0333897.s001.zip › Raw data/Figure6/Image/EXOs@BMSC1.jpg]

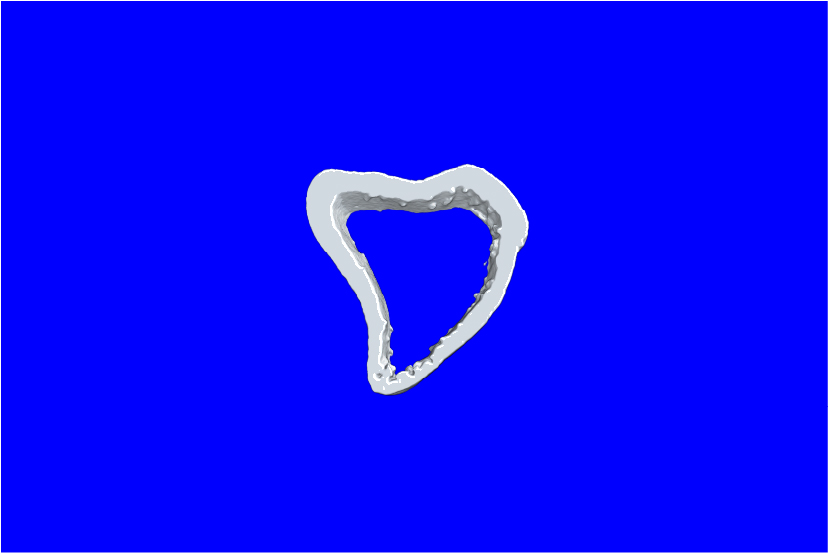

Supplement: S1 File — (ZIP) [file pone.0333897.s001.zip › Raw data/Figure6/Image/PBS.jpg]

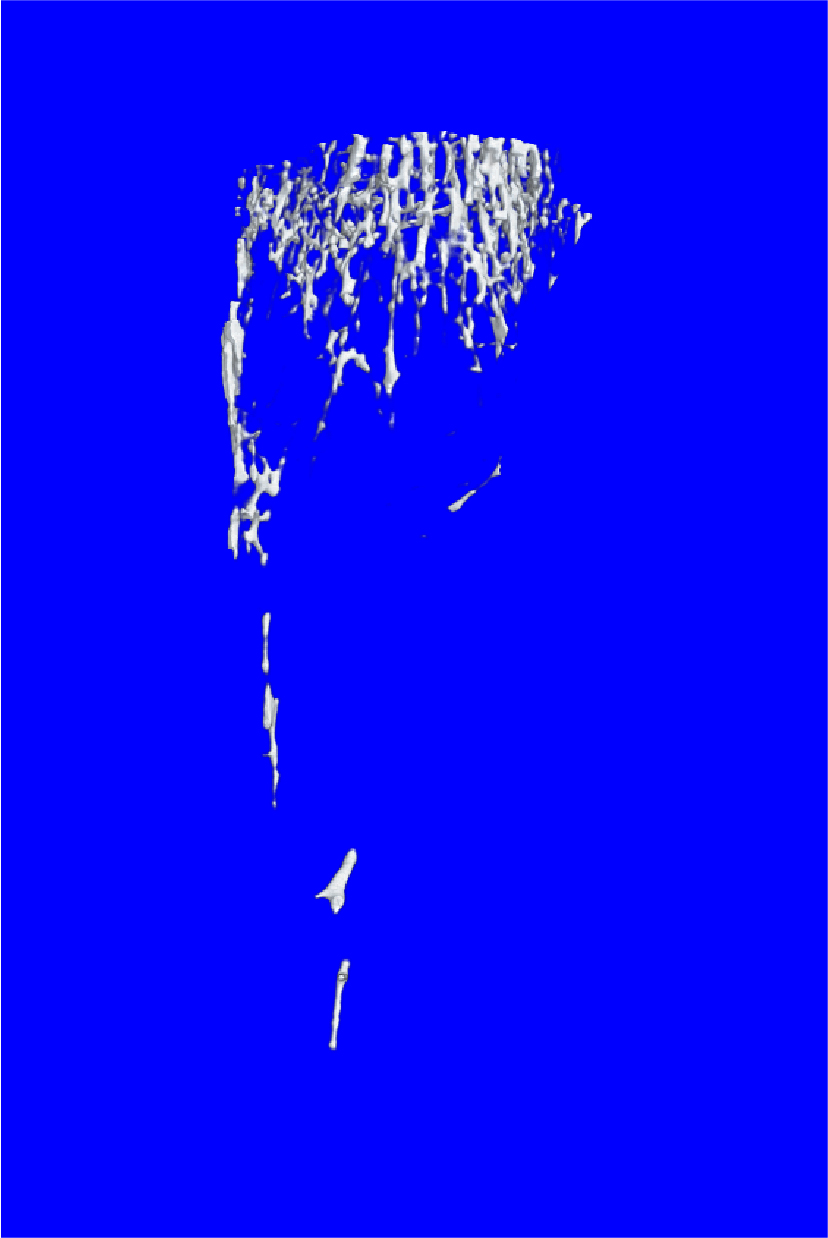

Supplement: S1 File — (ZIP) [file pone.0333897.s001.zip › Raw data/Figure6/Image/PBS1.jpg]

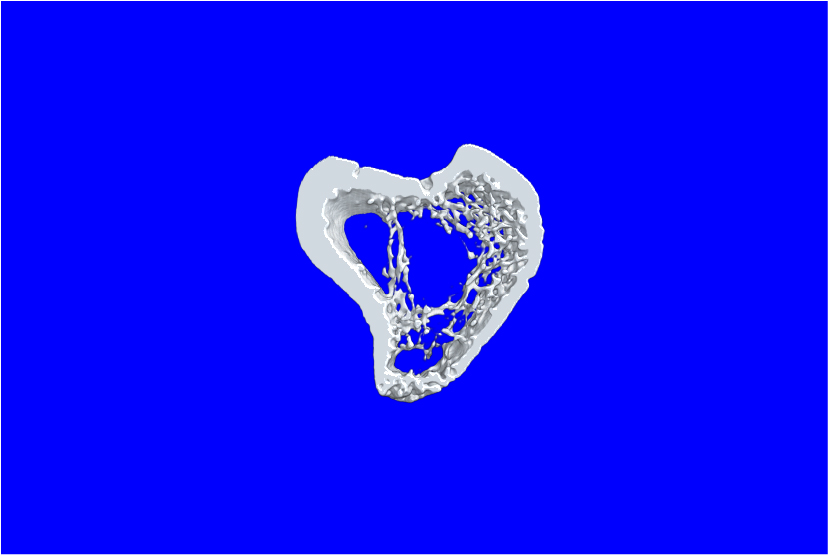

Supplement: S1 File — (ZIP) [file pone.0333897.s001.zip › Raw data/Figure6/Image/S-EXOs@BMSC.jpg]

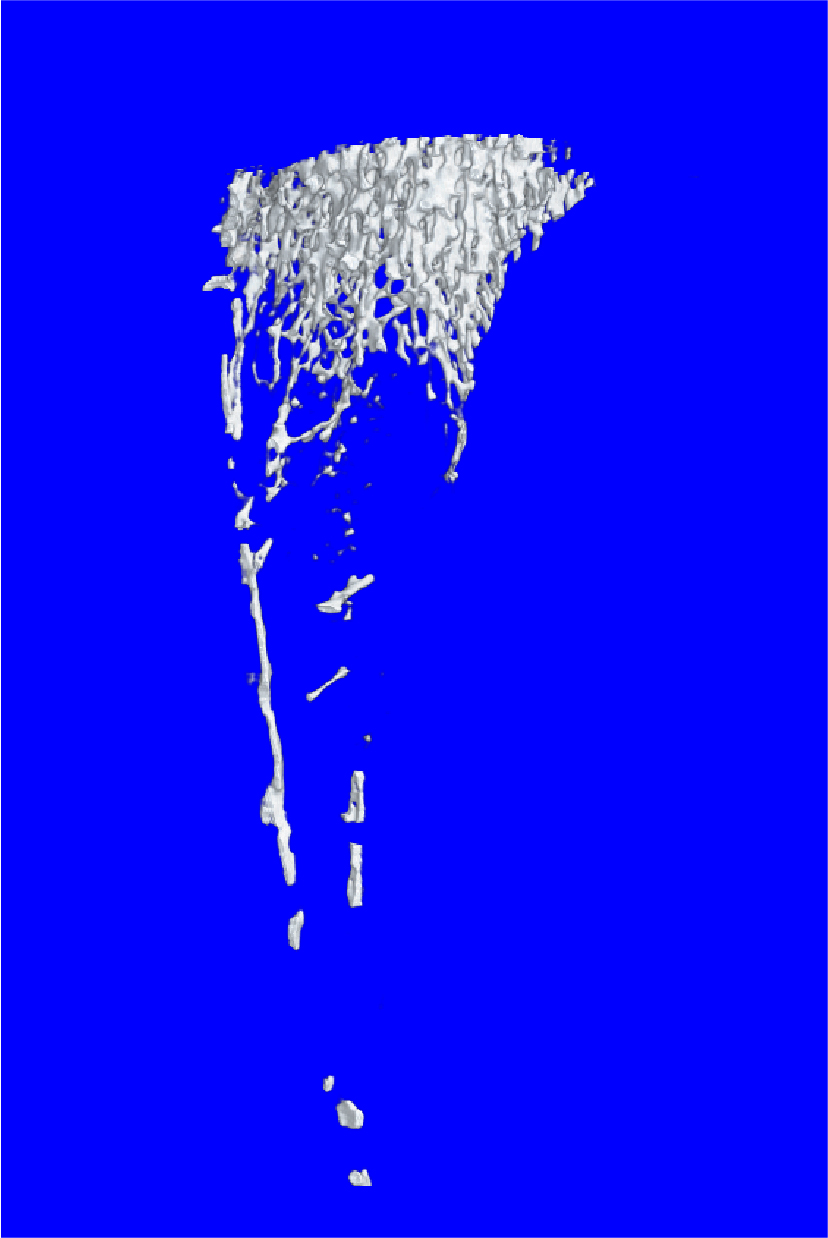

Supplement: S1 File — (ZIP) [file pone.0333897.s001.zip › Raw data/Figure6/Image/S-EXOs@BMSC1.jpg]

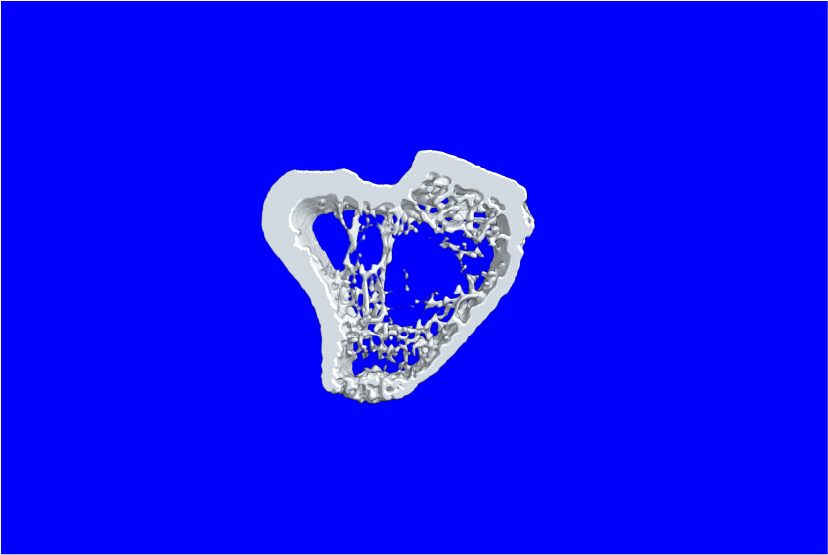

Supplement: S1 File — (ZIP) [file pone.0333897.s001.zip › Raw data/Figure6/Image/sham.jpg]

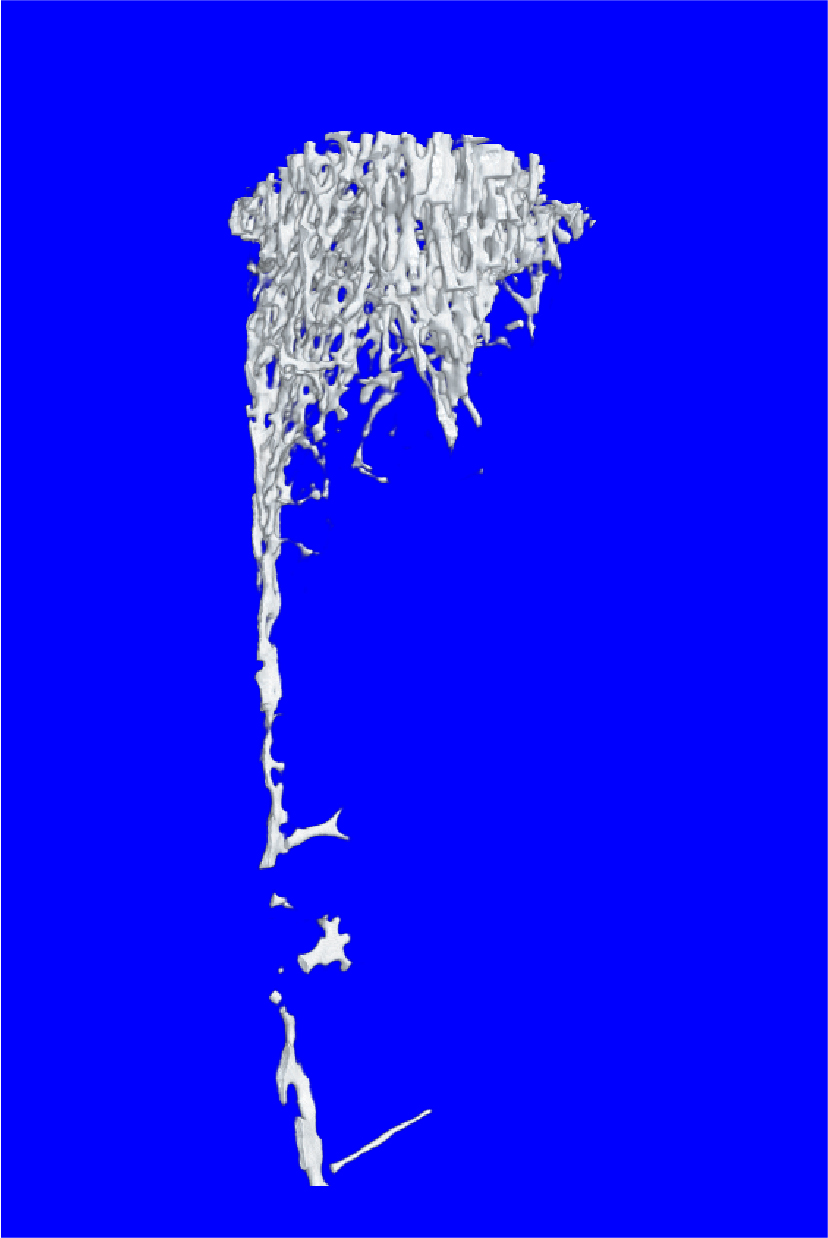

Supplement: S1 File — (ZIP) [file pone.0333897.s001.zip › Raw data/Figure6/Image/Sham1.jpg]

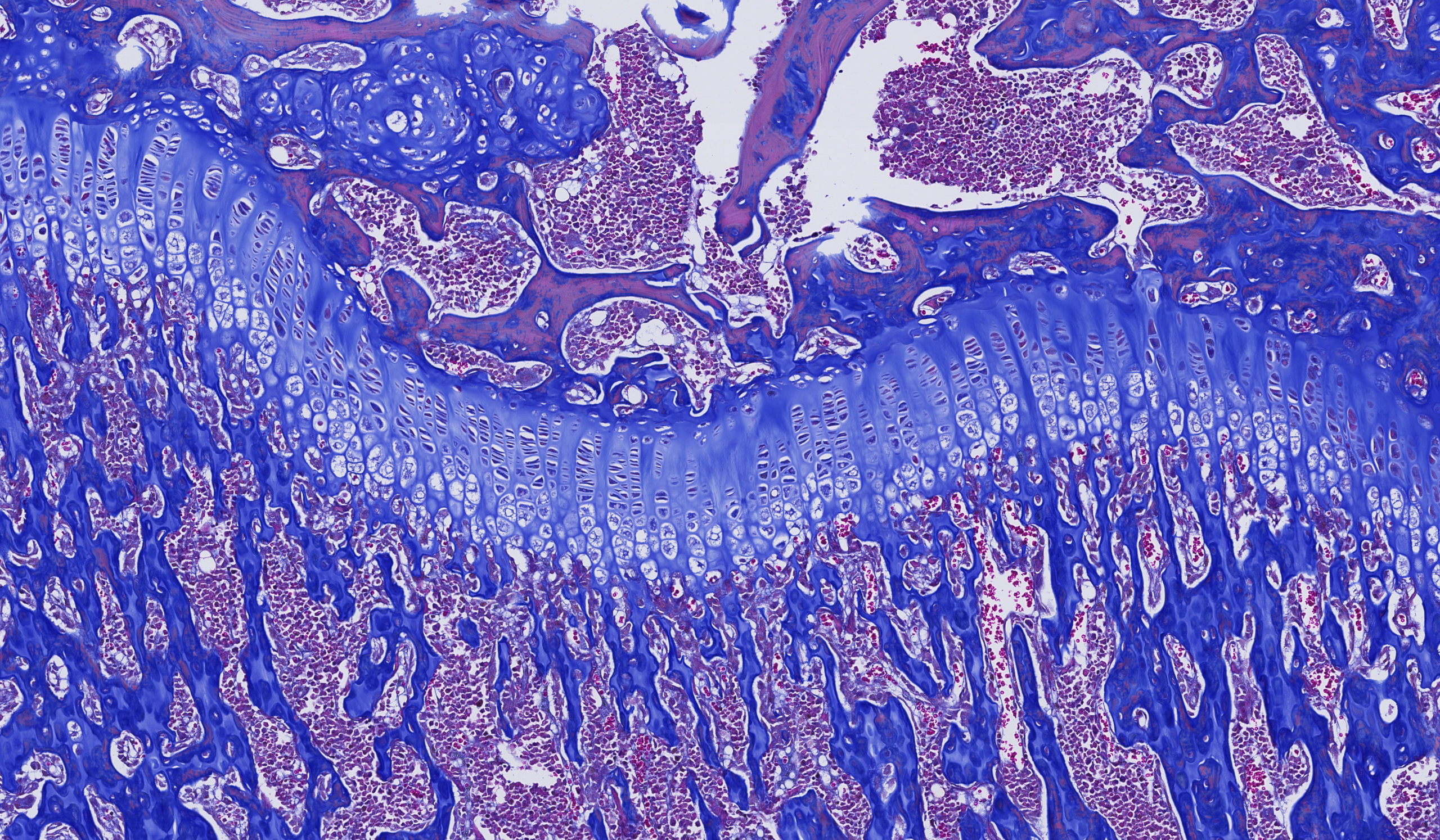

Supplement: S1 File — (ZIP) [file pone.0333897.s001.zip › Raw data/Figure7/HE/1.jpg]

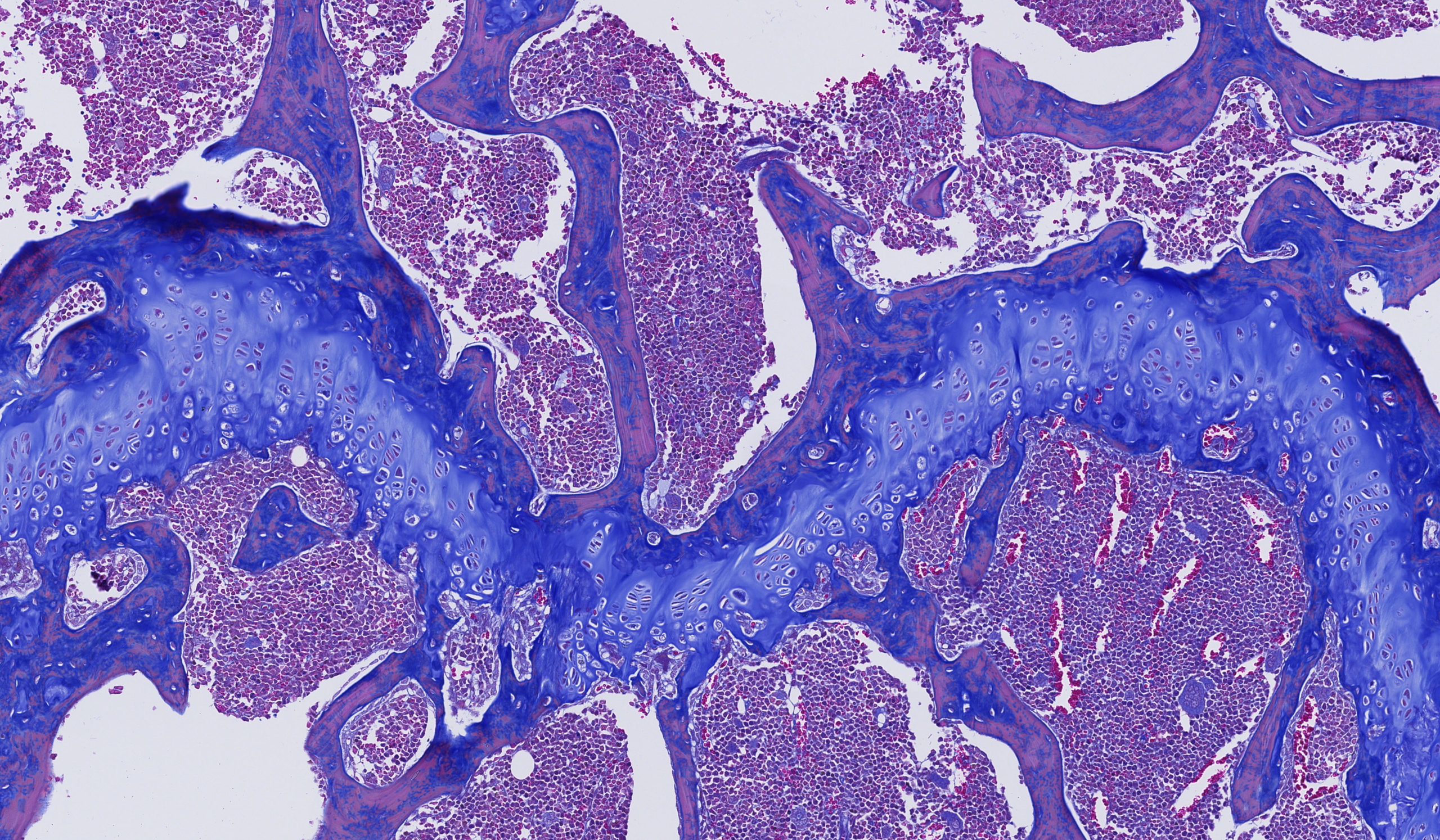

Supplement: S1 File — (ZIP) [file pone.0333897.s001.zip › Raw data/Figure7/HE/2.jpg]

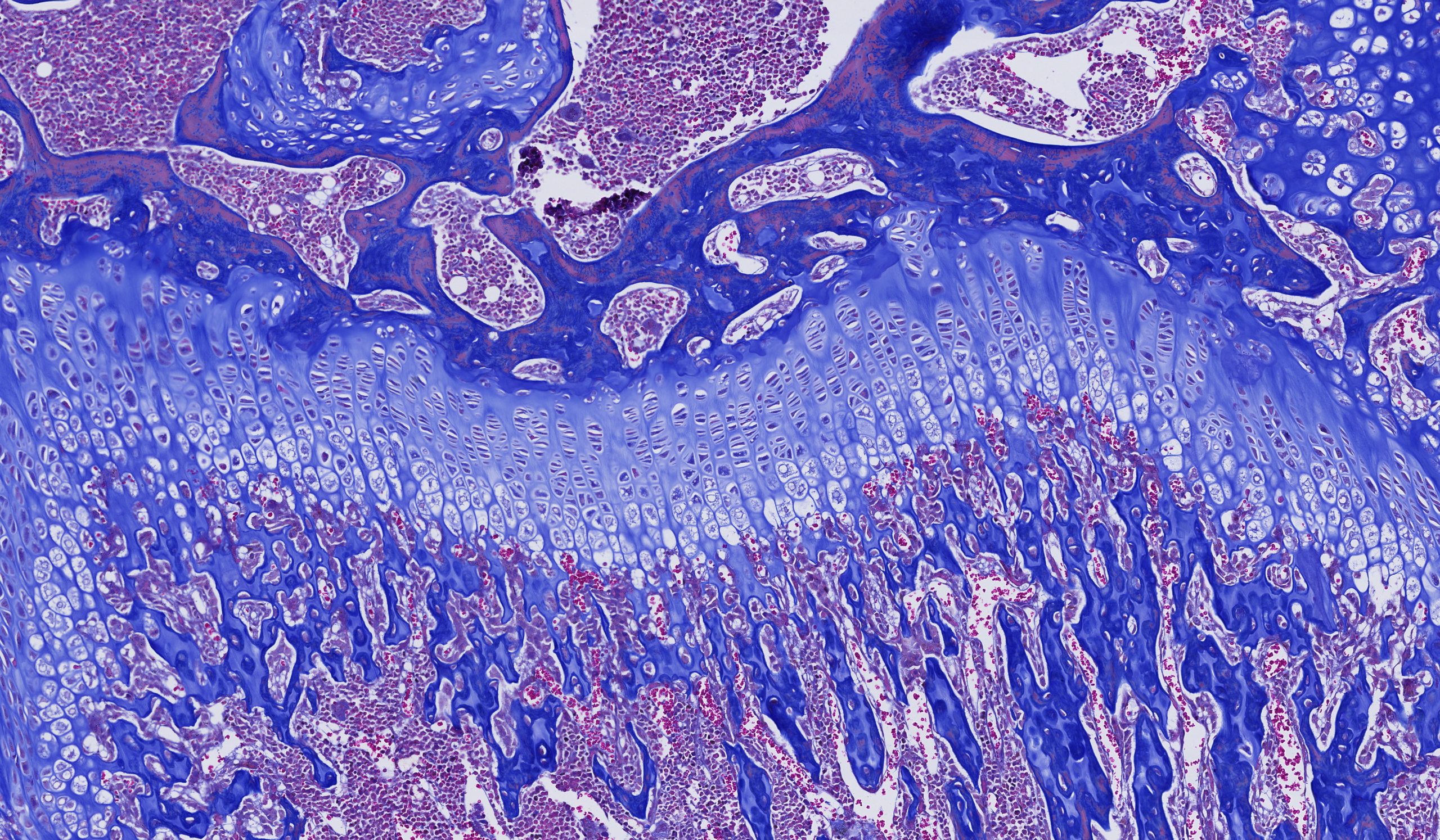

Supplement: S1 File — (ZIP) [file pone.0333897.s001.zip › Raw data/Figure7/HE/3.jpg]

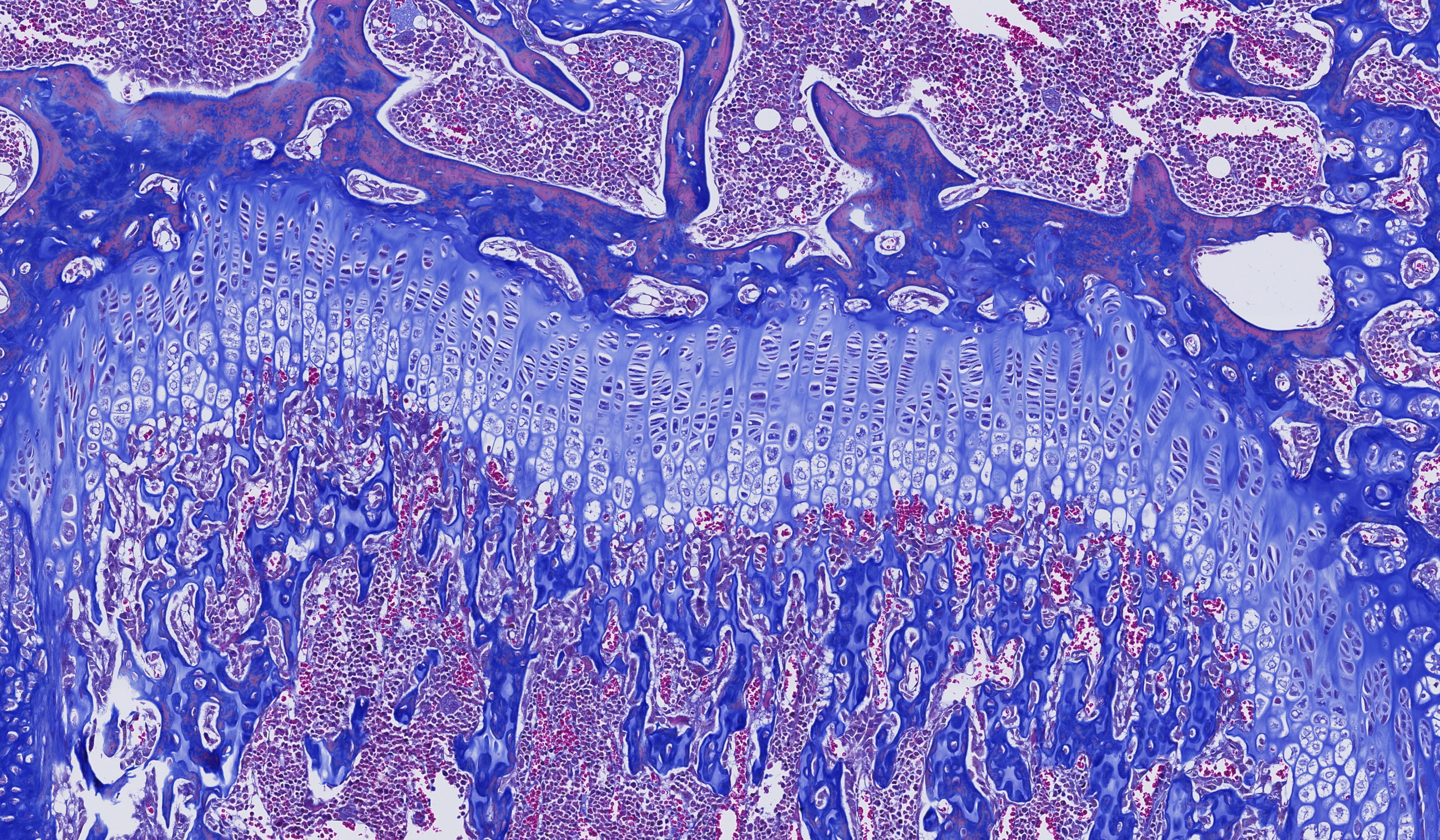

Supplement: S1 File — (ZIP) [file pone.0333897.s001.zip › Raw data/Figure7/HE/4.jpg]

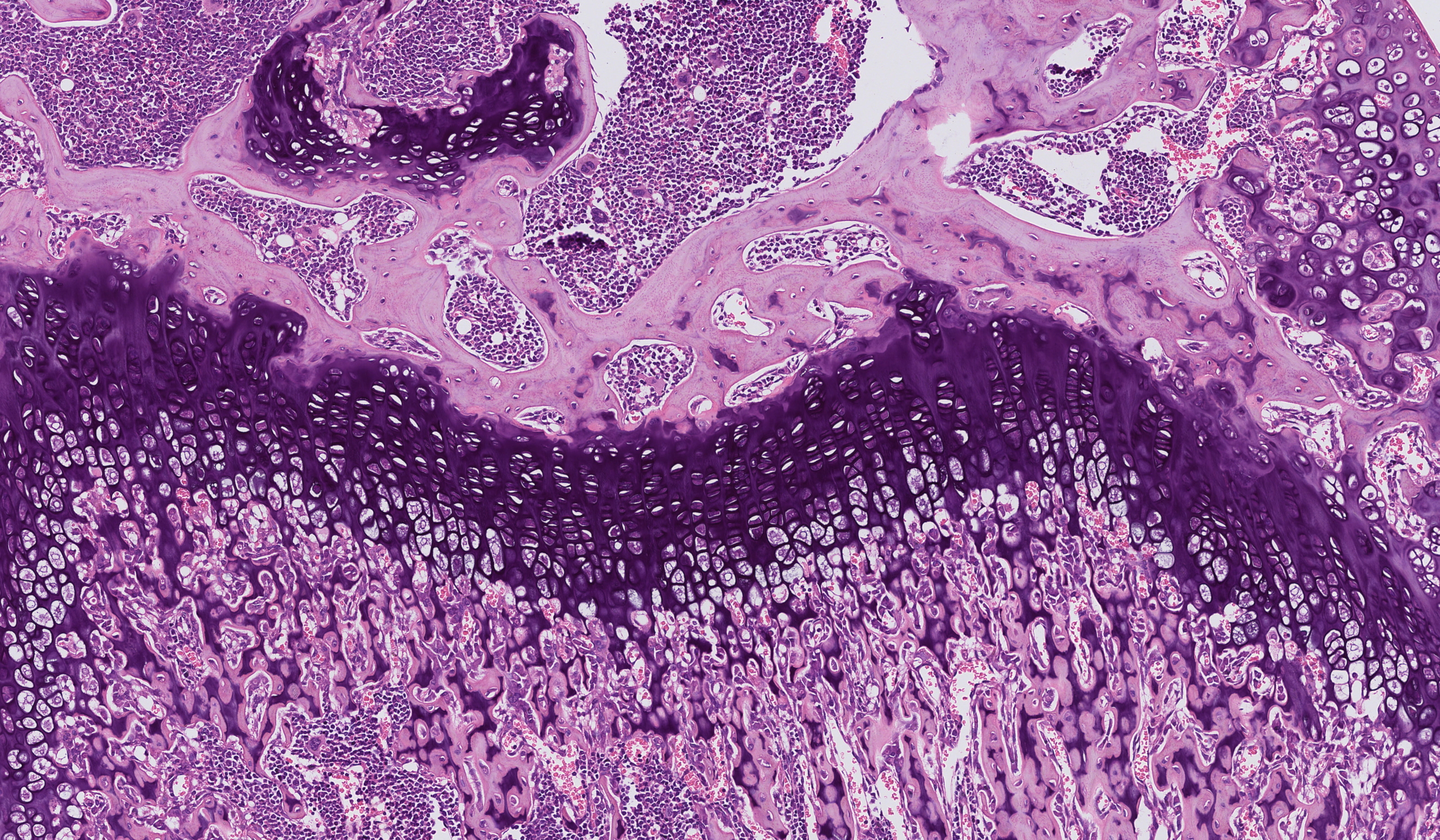

Supplement: S1 File — (ZIP) [file pone.0333897.s001.zip › Raw data/Figure7/HE/5.jpg]

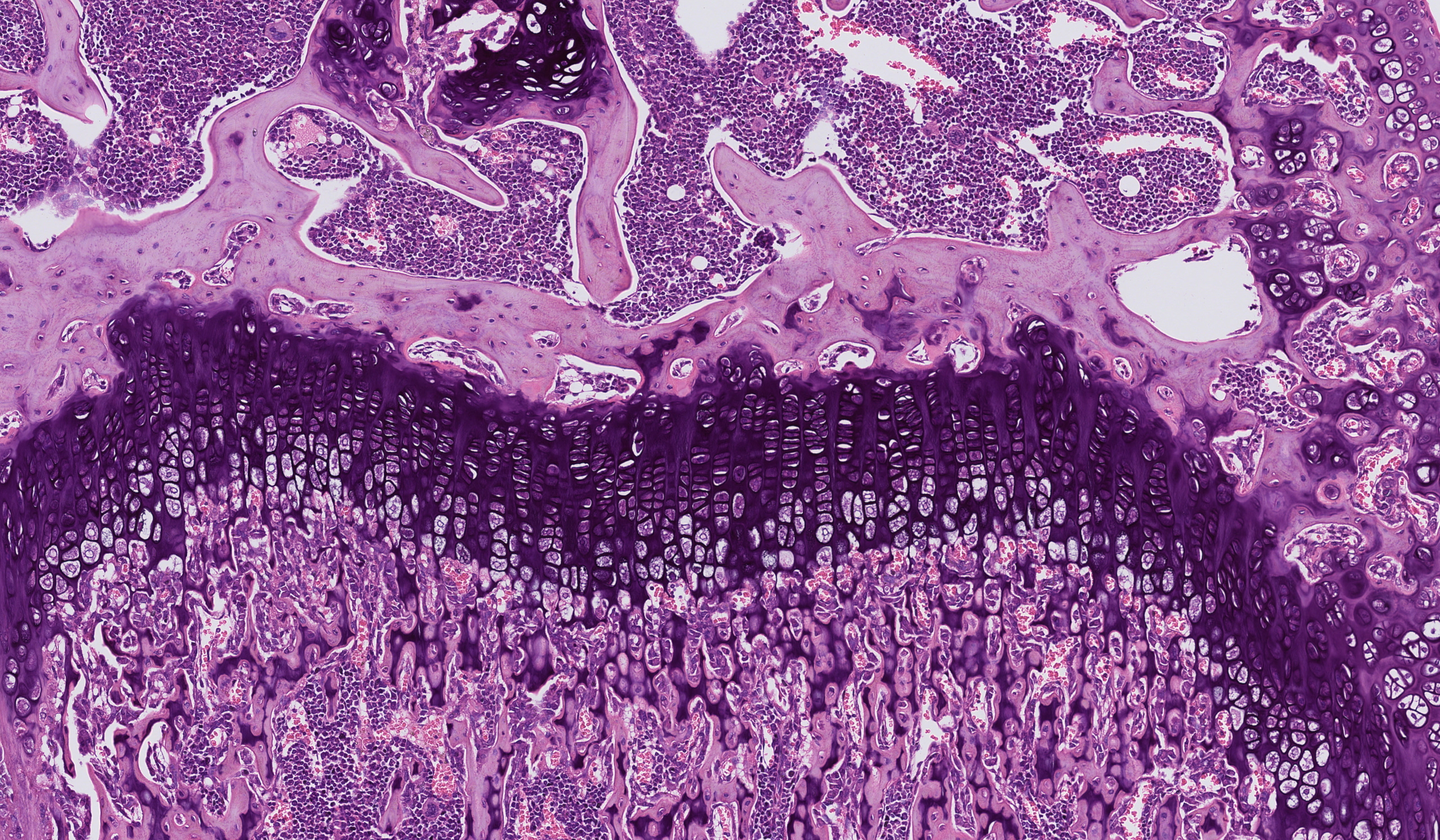

Supplement: S1 File — (ZIP) [file pone.0333897.s001.zip › Raw data/Figure7/HE/6.jpg]

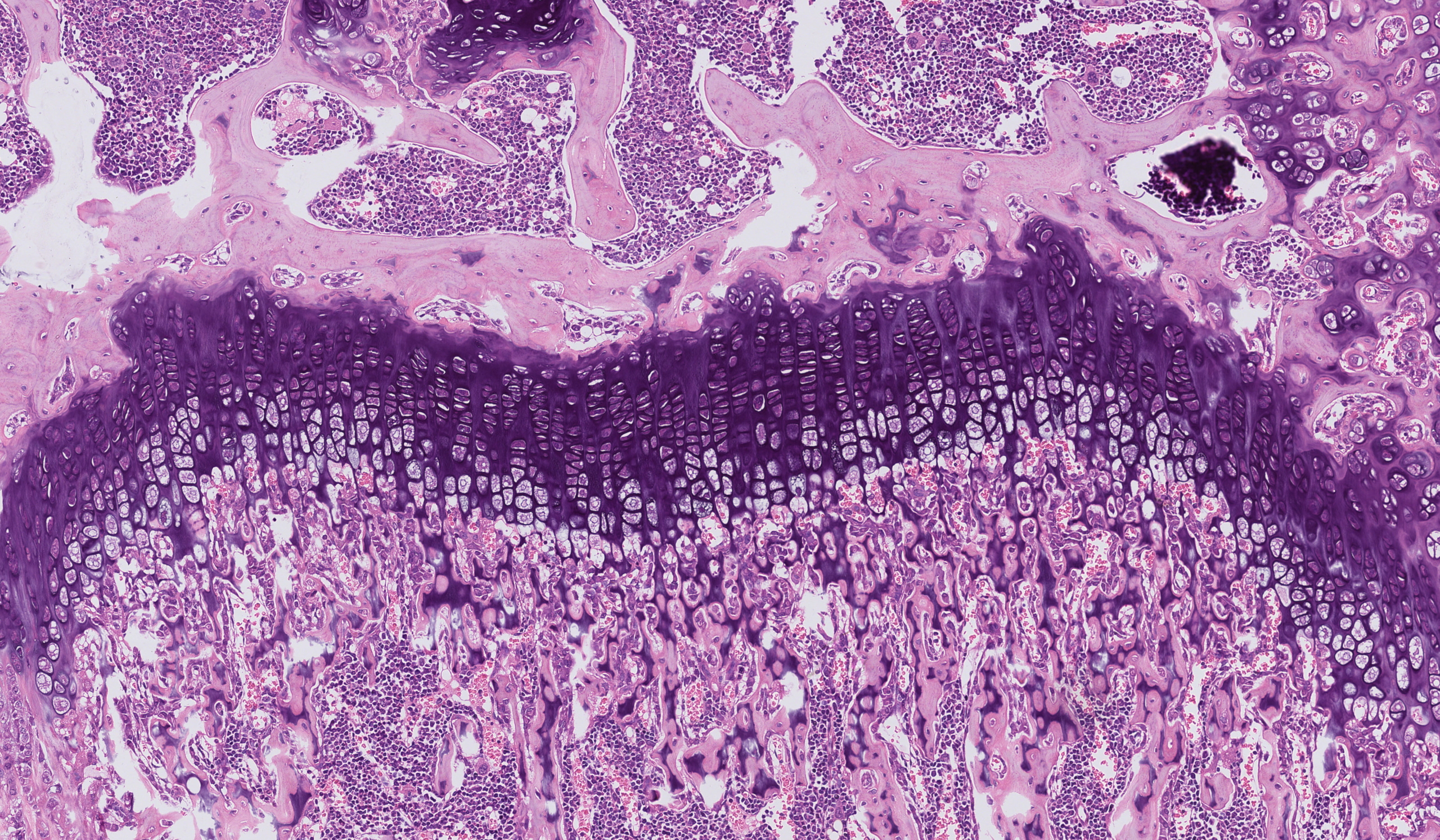

Supplement: S1 File — (ZIP) [file pone.0333897.s001.zip › Raw data/Figure7/HE/7.jpg]

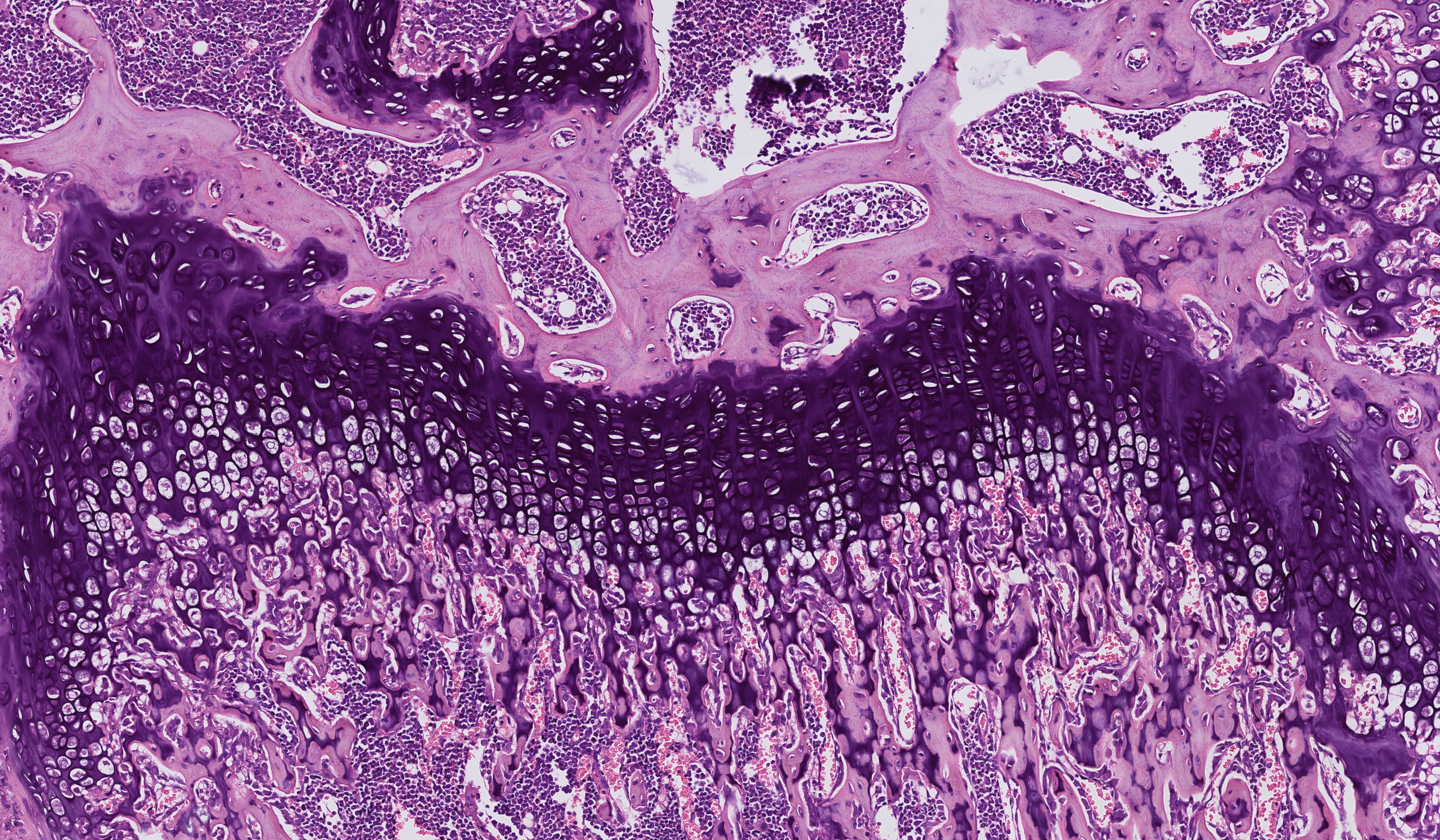

Supplement: S1 File — (ZIP) [file pone.0333897.s001.zip › Raw data/Figure7/HE/8.jpg]

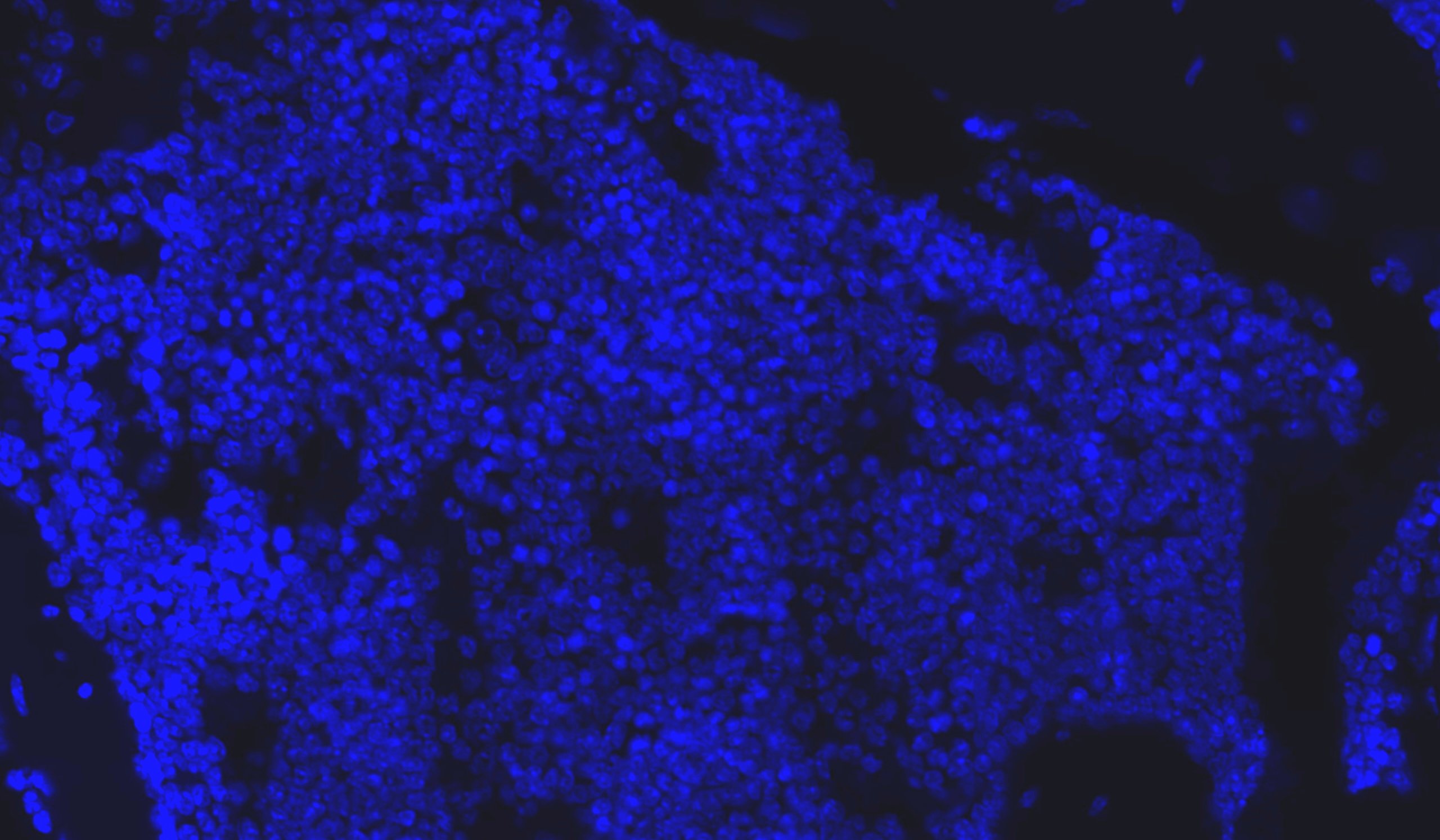

Supplement: S1 File — (ZIP) [file pone.0333897.s001.zip › Raw data/Figure7/IF-mice/1.jpg]

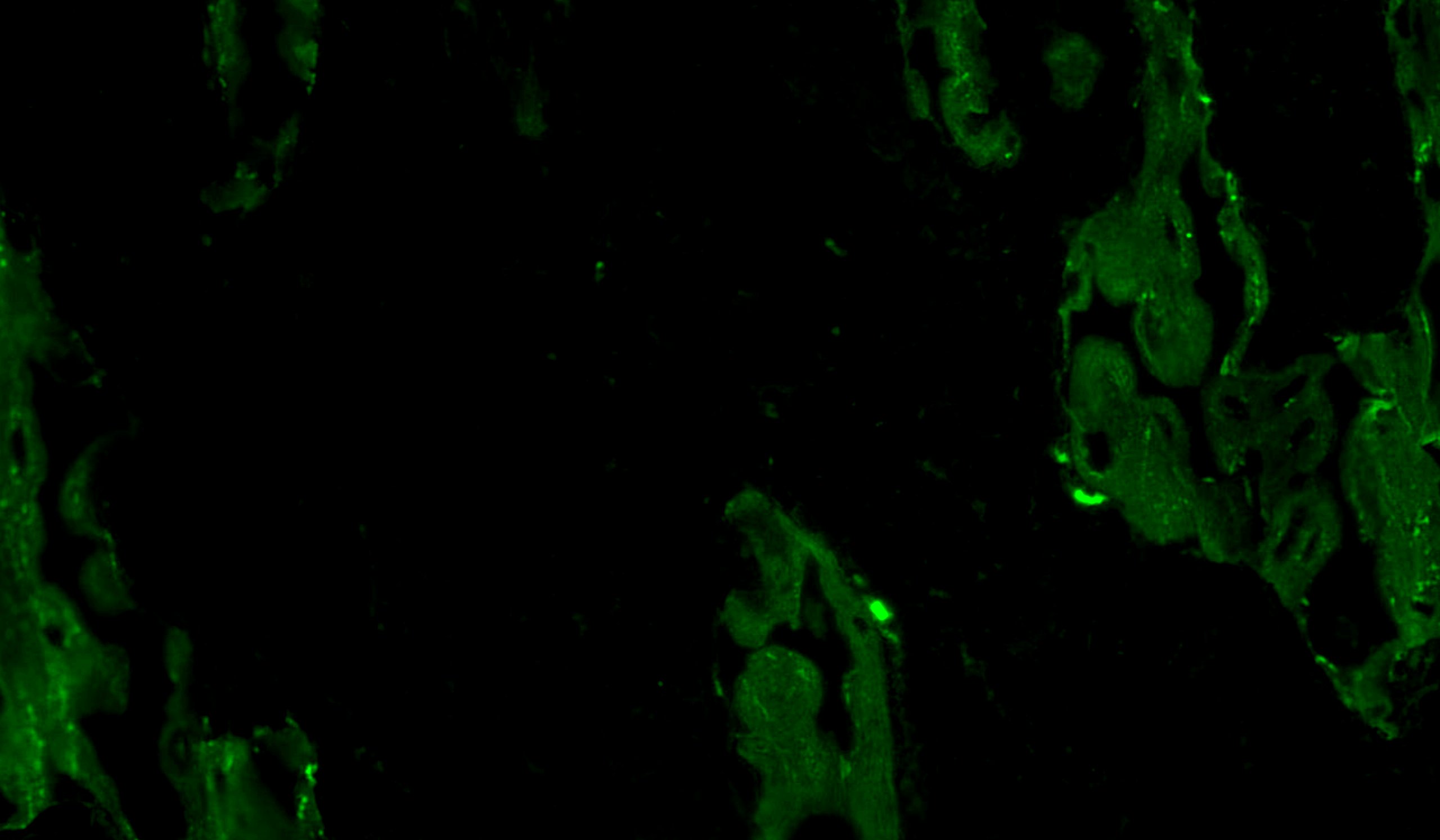

Supplement: S1 File — (ZIP) [file pone.0333897.s001.zip › Raw data/Figure7/IF-mice/10.jpg]

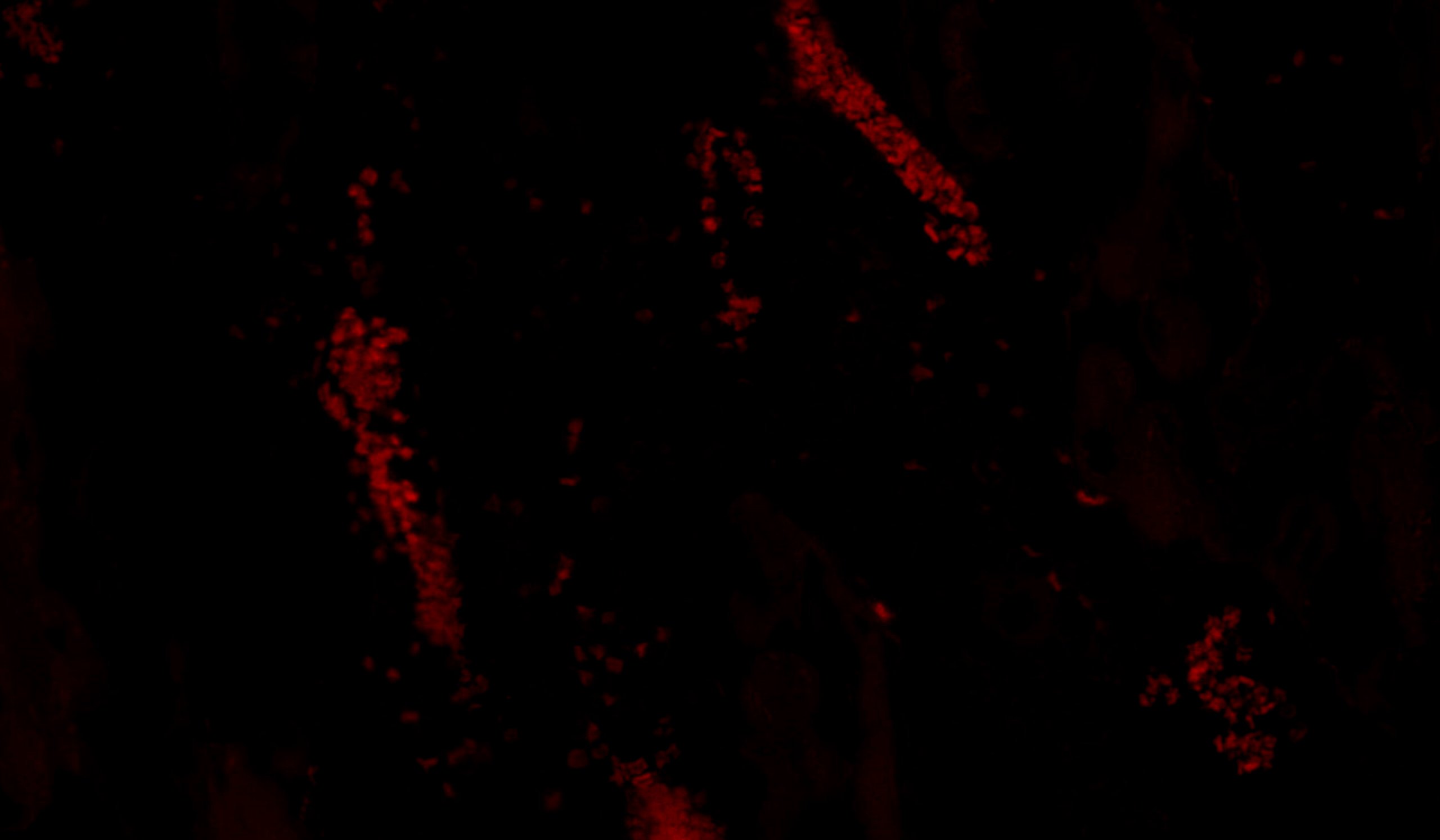

Supplement: S1 File — (ZIP) [file pone.0333897.s001.zip › Raw data/Figure7/IF-mice/11.jpg]

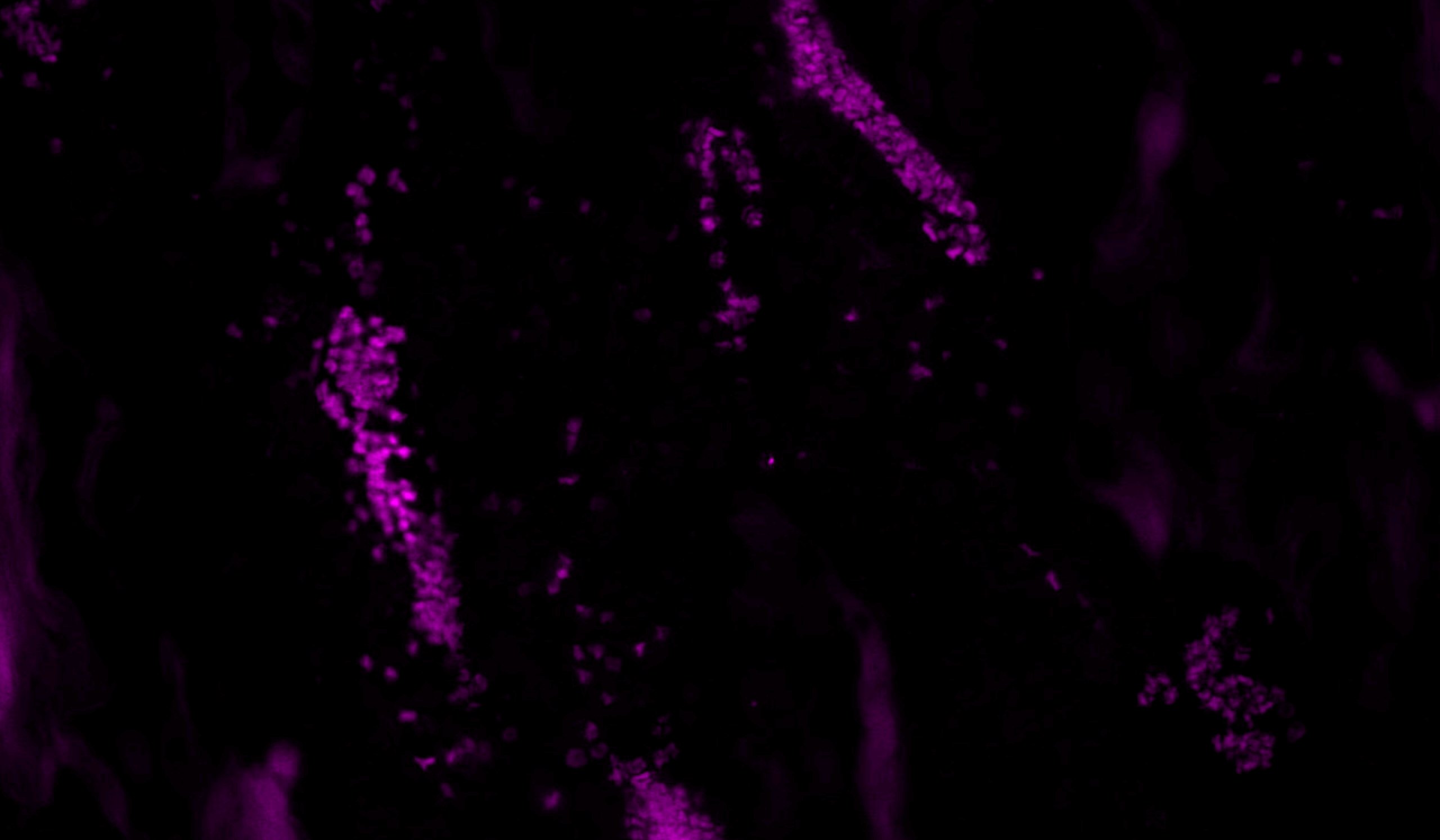

Supplement: S1 File — (ZIP) [file pone.0333897.s001.zip › Raw data/Figure7/IF-mice/12.jpg]

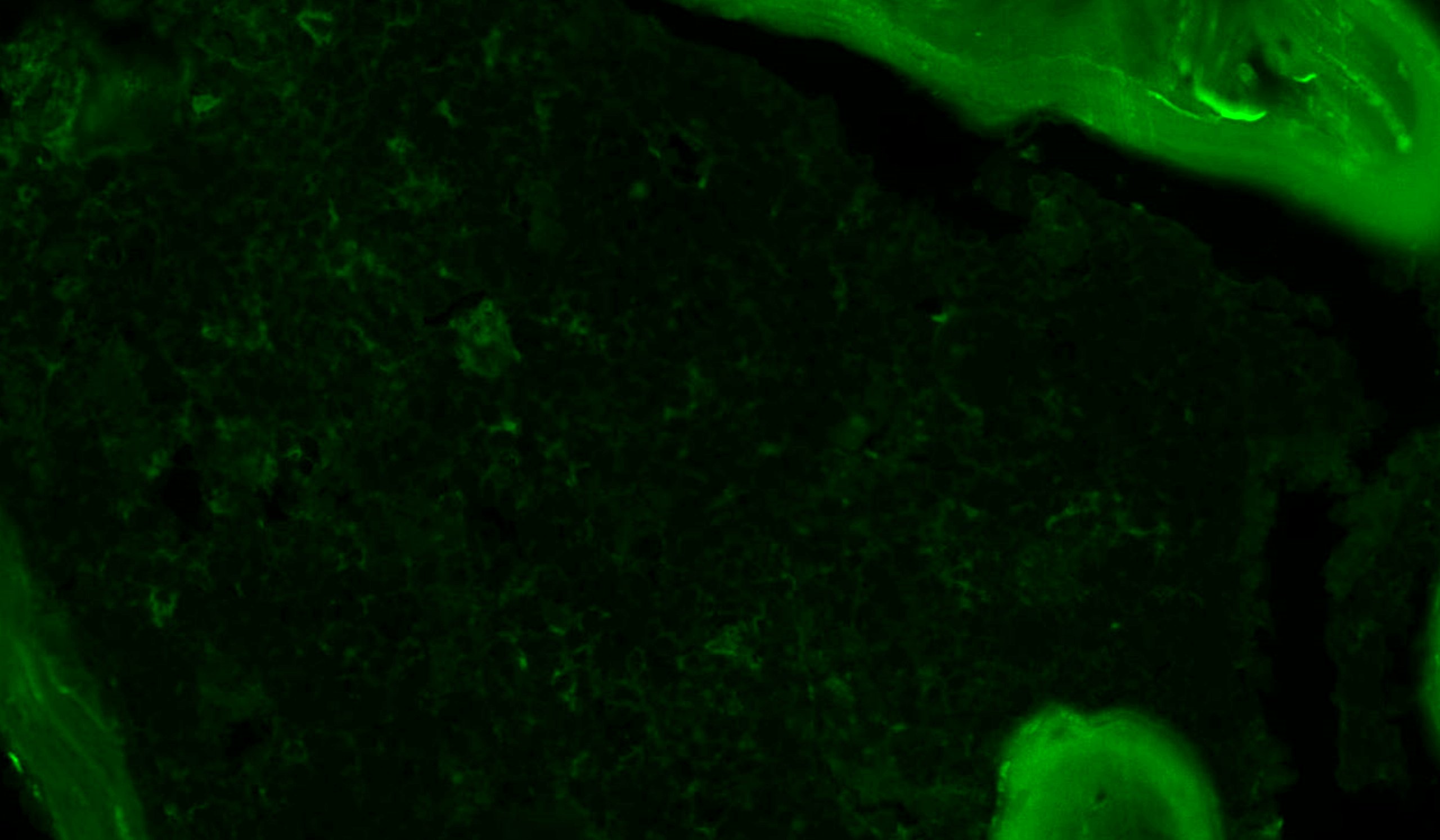

Supplement: S1 File — (ZIP) [file pone.0333897.s001.zip › Raw data/Figure7/IF-mice/2.jpg]

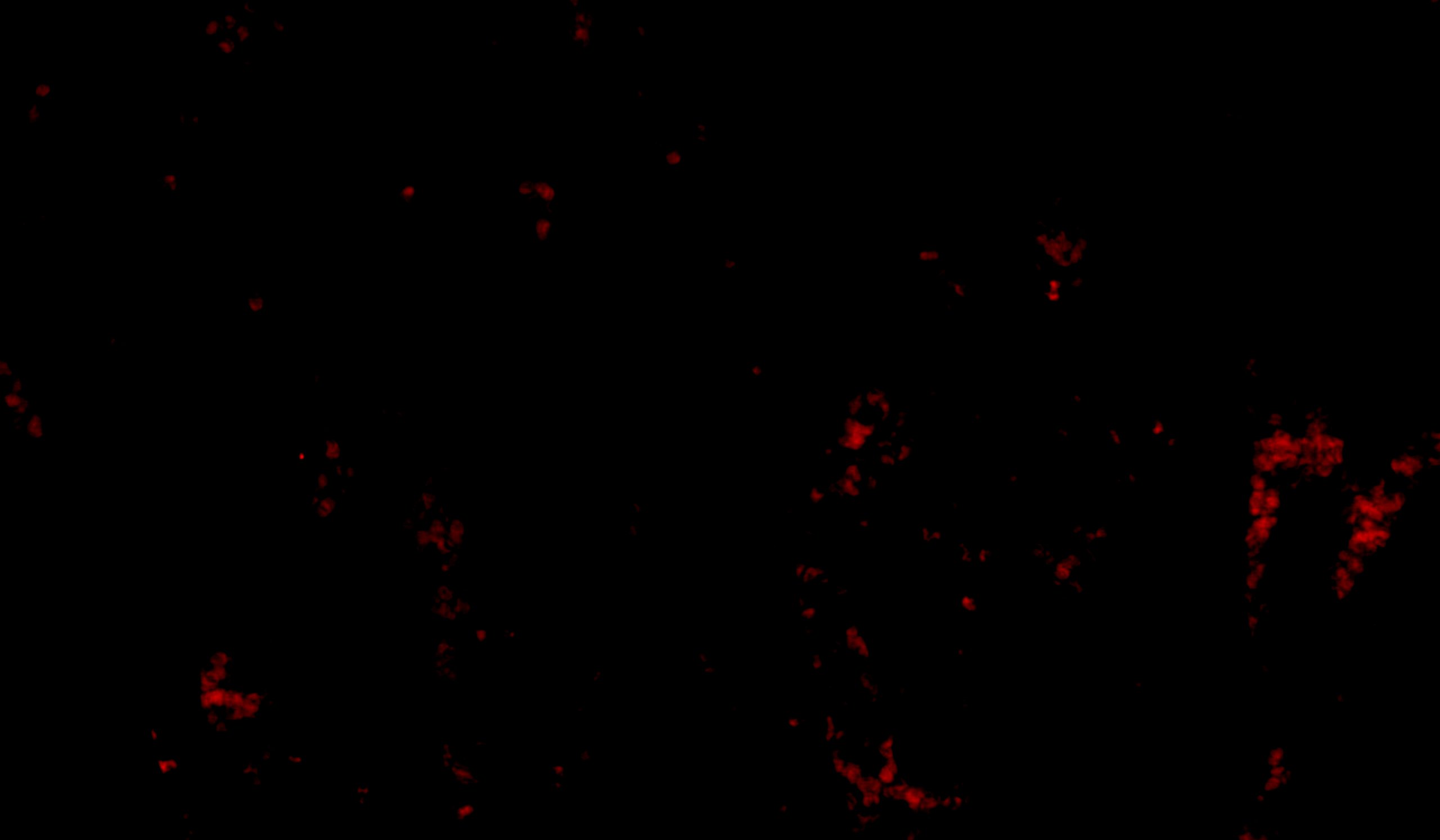

Supplement: S1 File — (ZIP) [file pone.0333897.s001.zip › Raw data/Figure7/IF-mice/3.jpg]

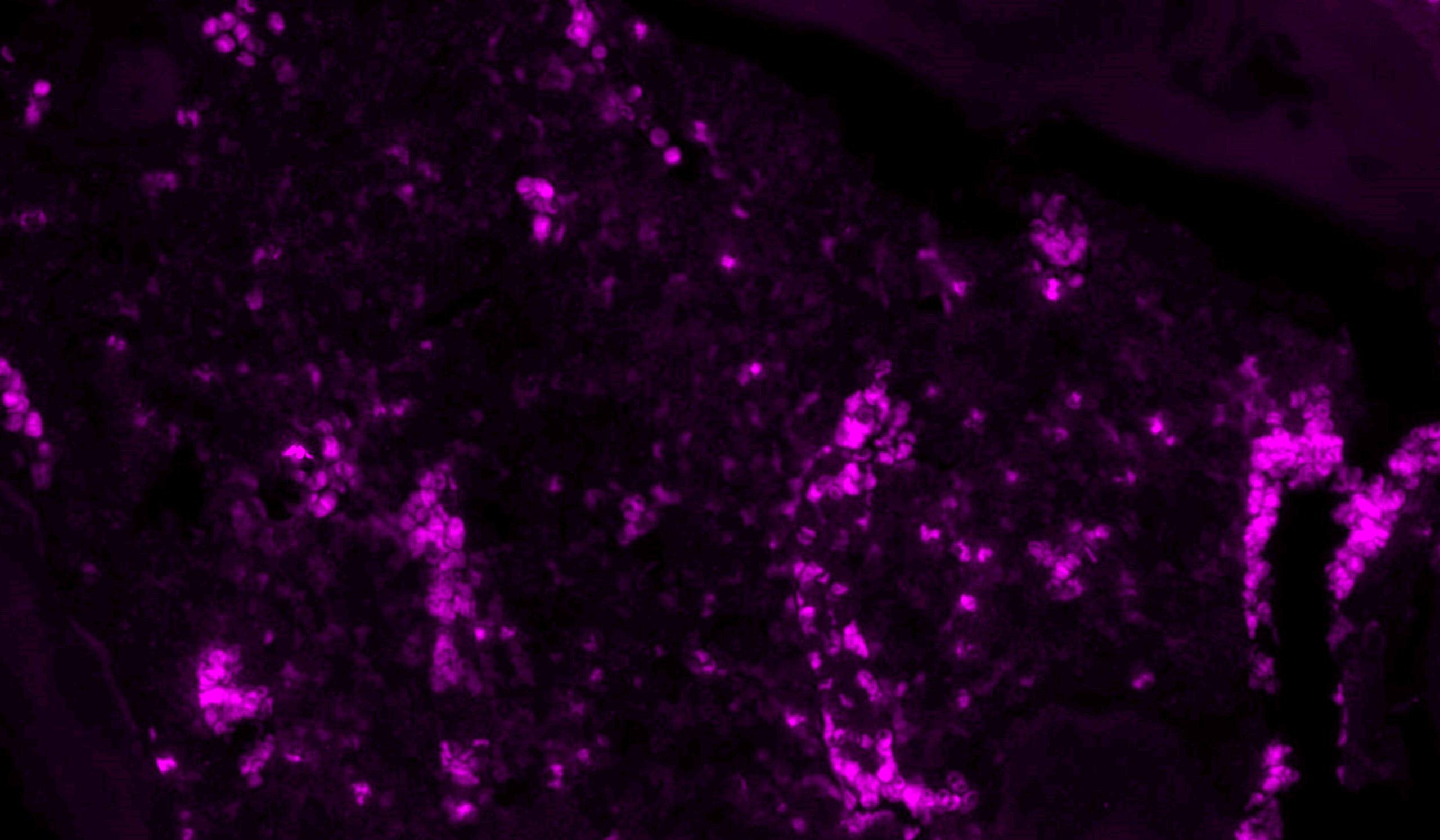

Supplement: S1 File — (ZIP) [file pone.0333897.s001.zip › Raw data/Figure7/IF-mice/4.jpg]

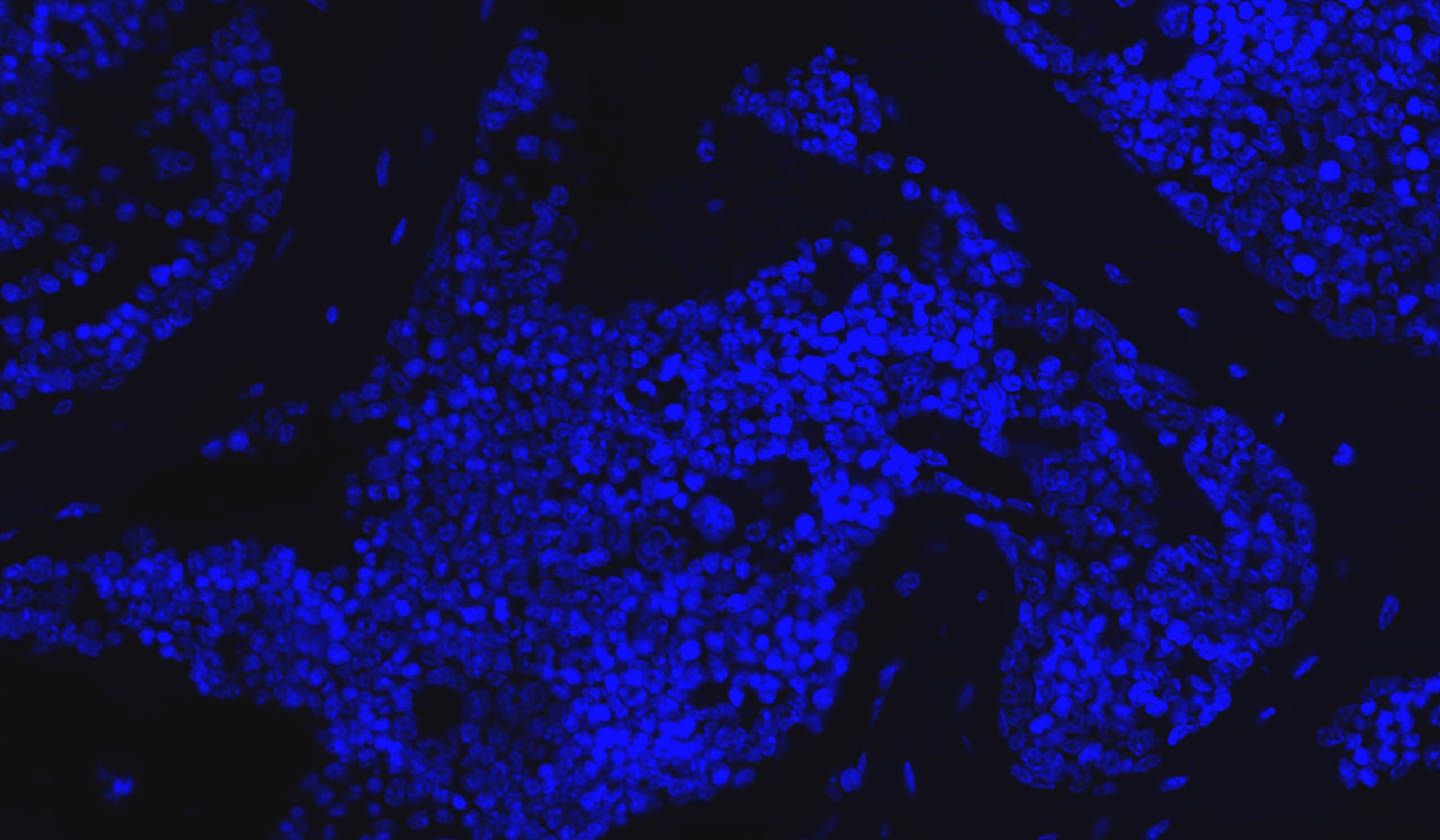

Supplement: S1 File — (ZIP) [file pone.0333897.s001.zip › Raw data/Figure7/IF-mice/5.jpg]

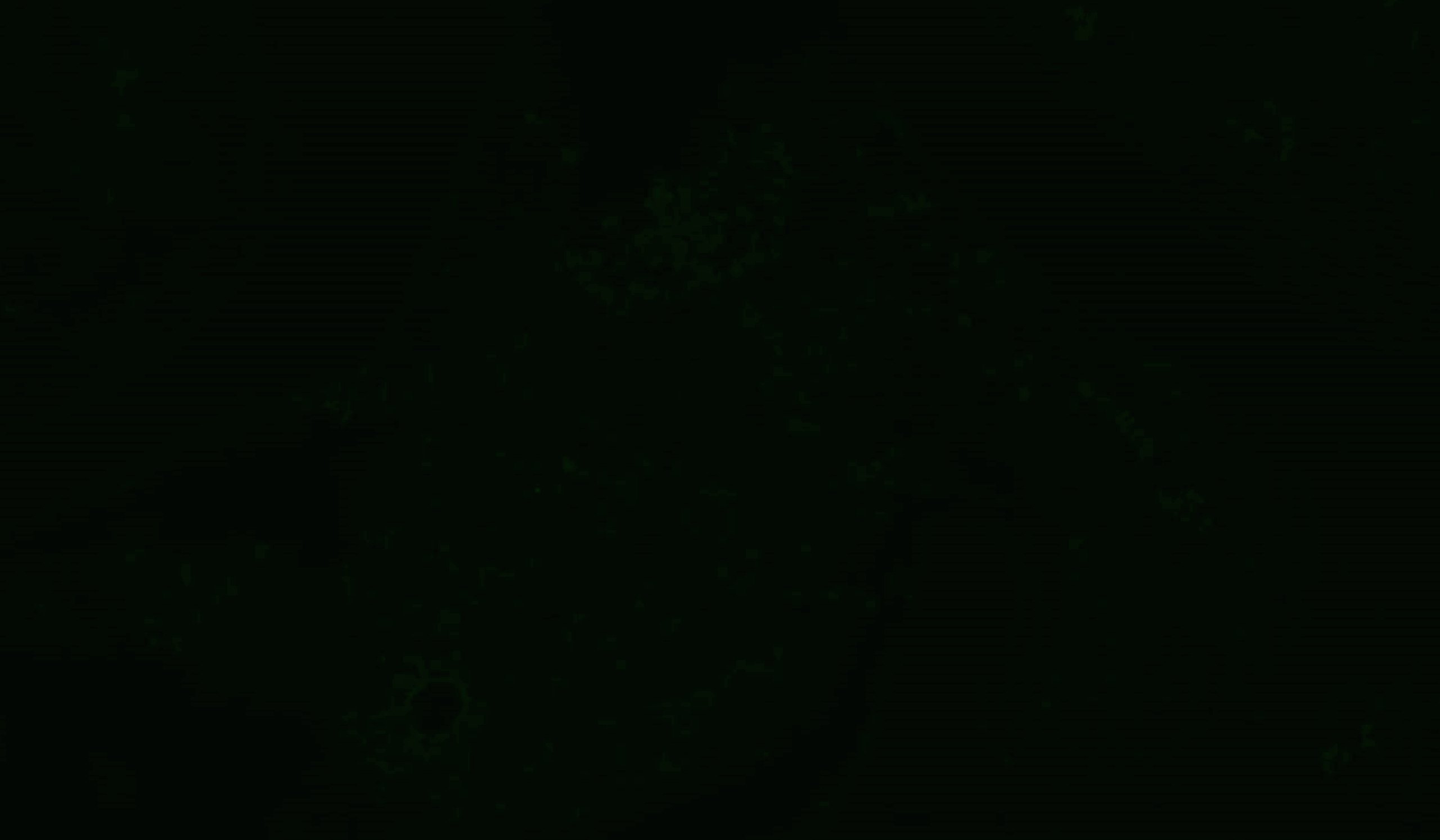

Supplement: S1 File — (ZIP) [file pone.0333897.s001.zip › Raw data/Figure7/IF-mice/6.jpg]

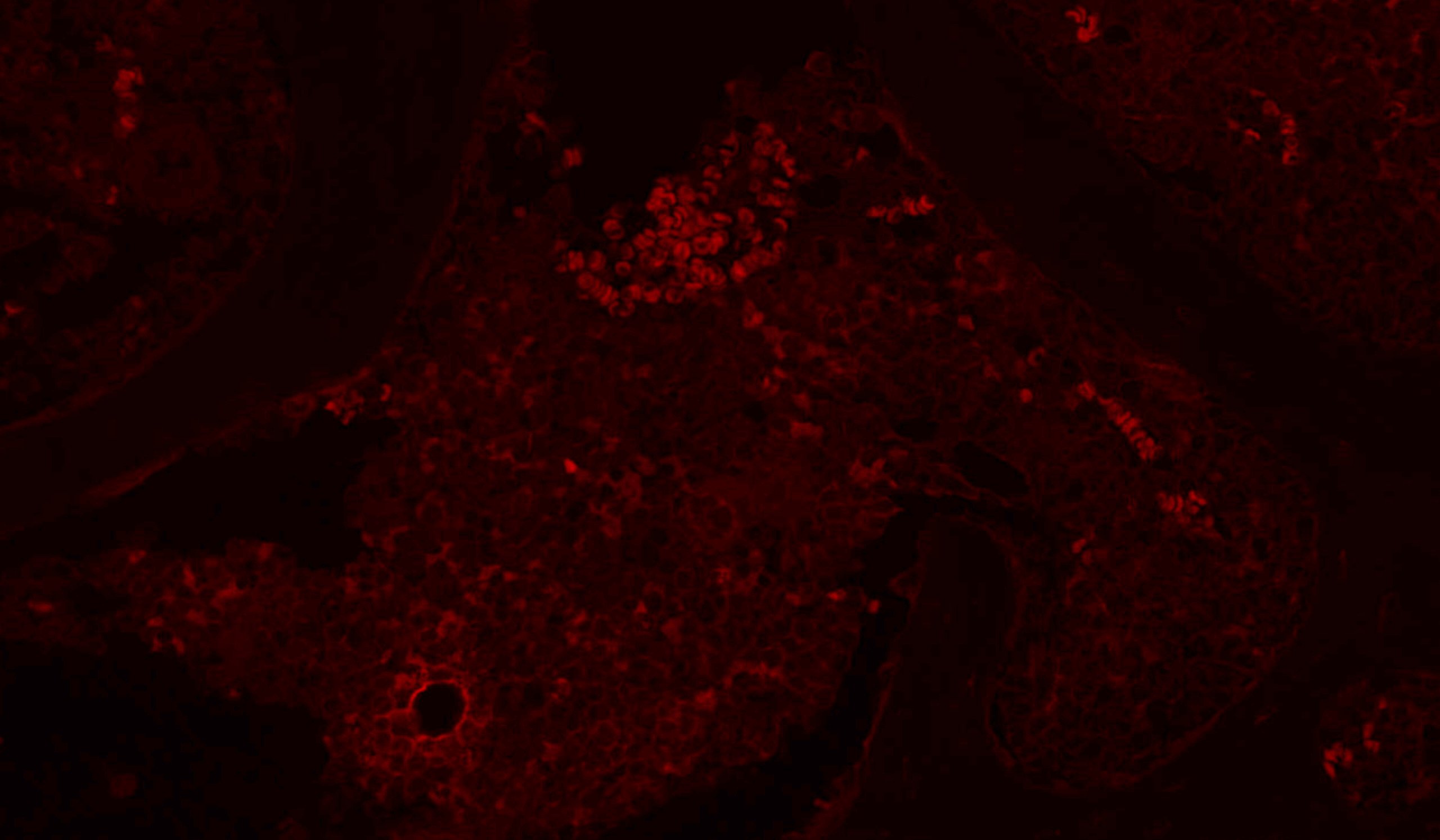

Supplement: S1 File — (ZIP) [file pone.0333897.s001.zip › Raw data/Figure7/IF-mice/7.jpg]

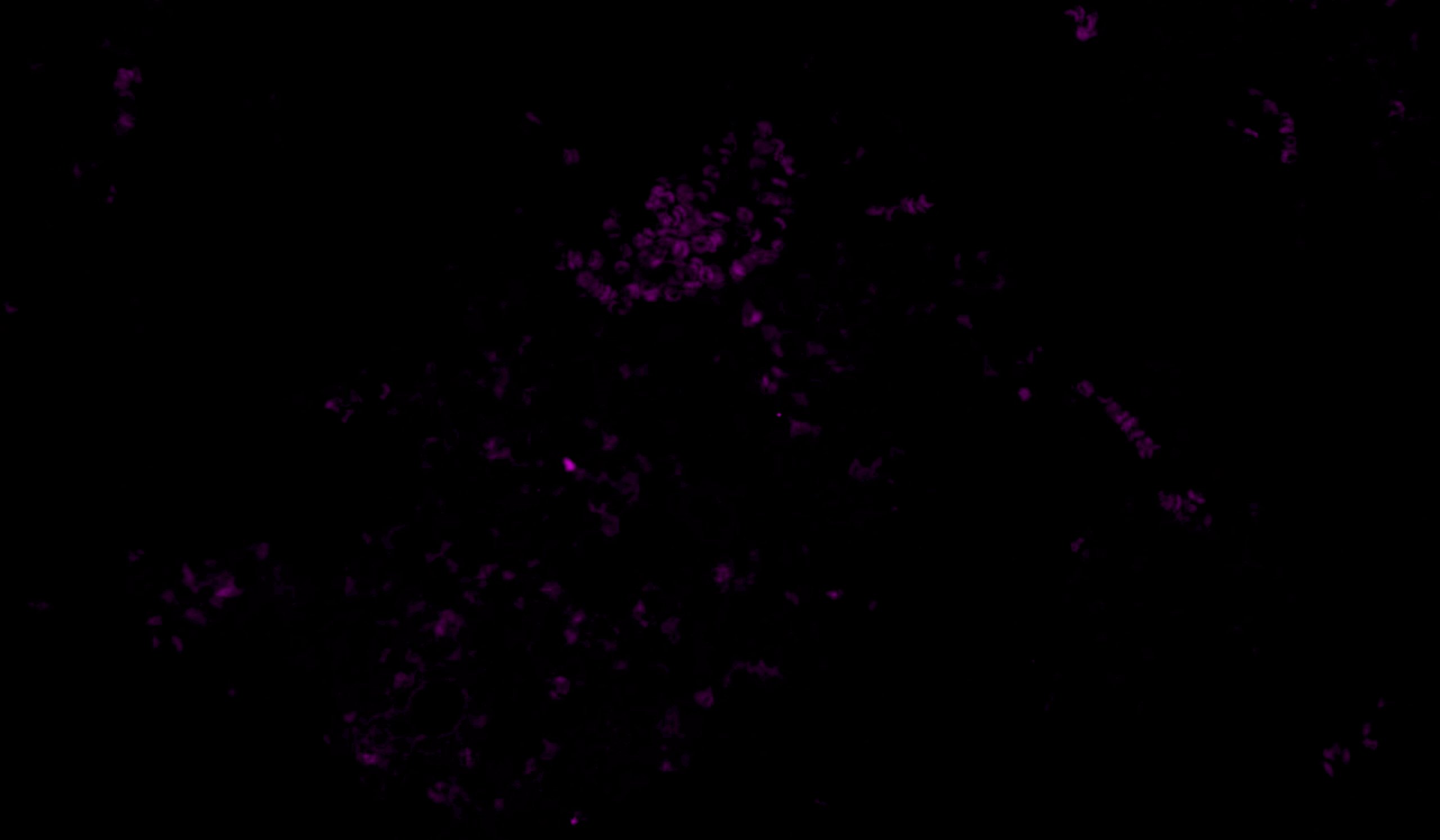

Supplement: S1 File — (ZIP) [file pone.0333897.s001.zip › Raw data/Figure7/IF-mice/8.jpg]

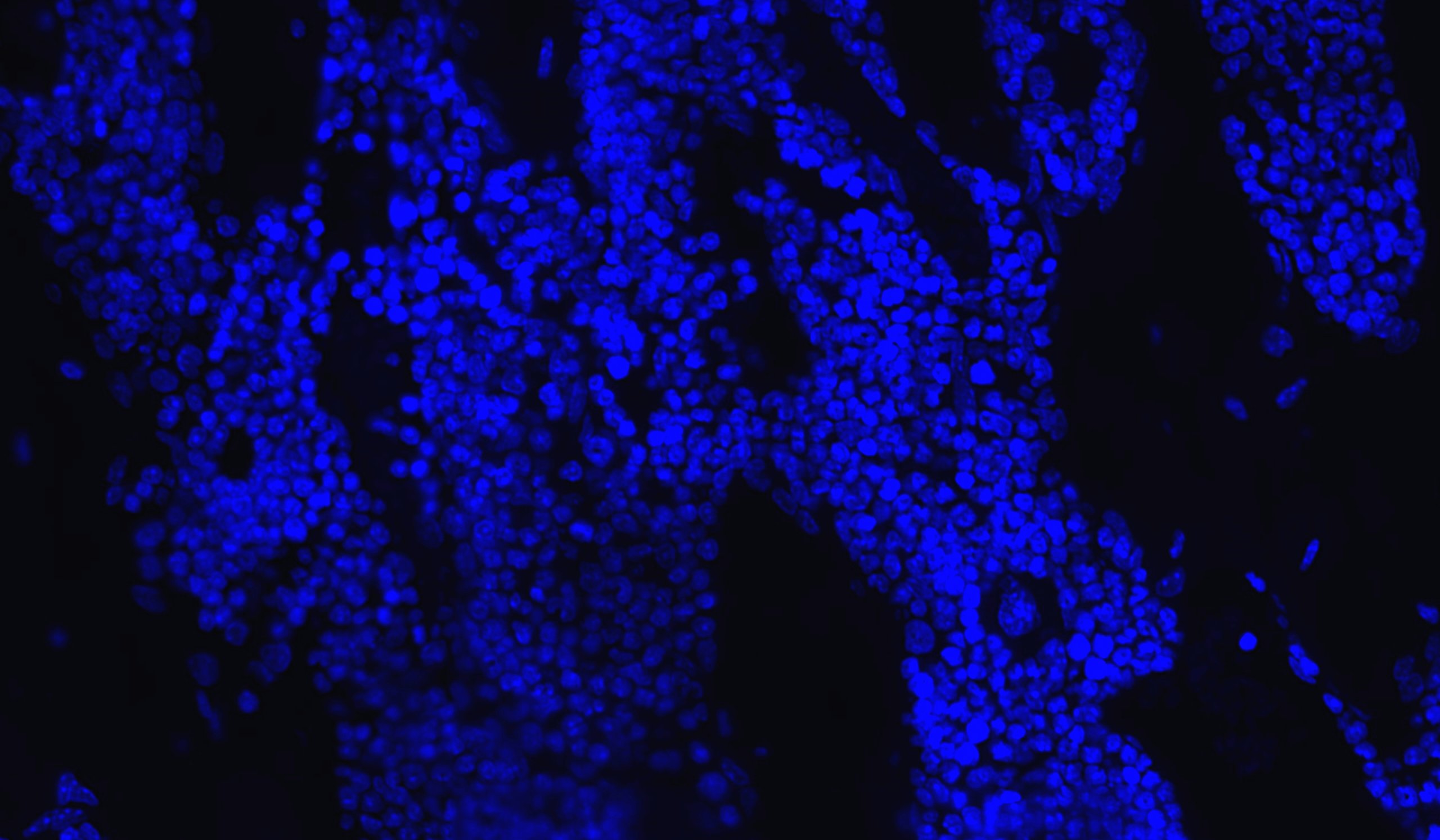

Supplement: S1 File — (ZIP) [file pone.0333897.s001.zip › Raw data/Figure7/IF-mice/9.jpg]

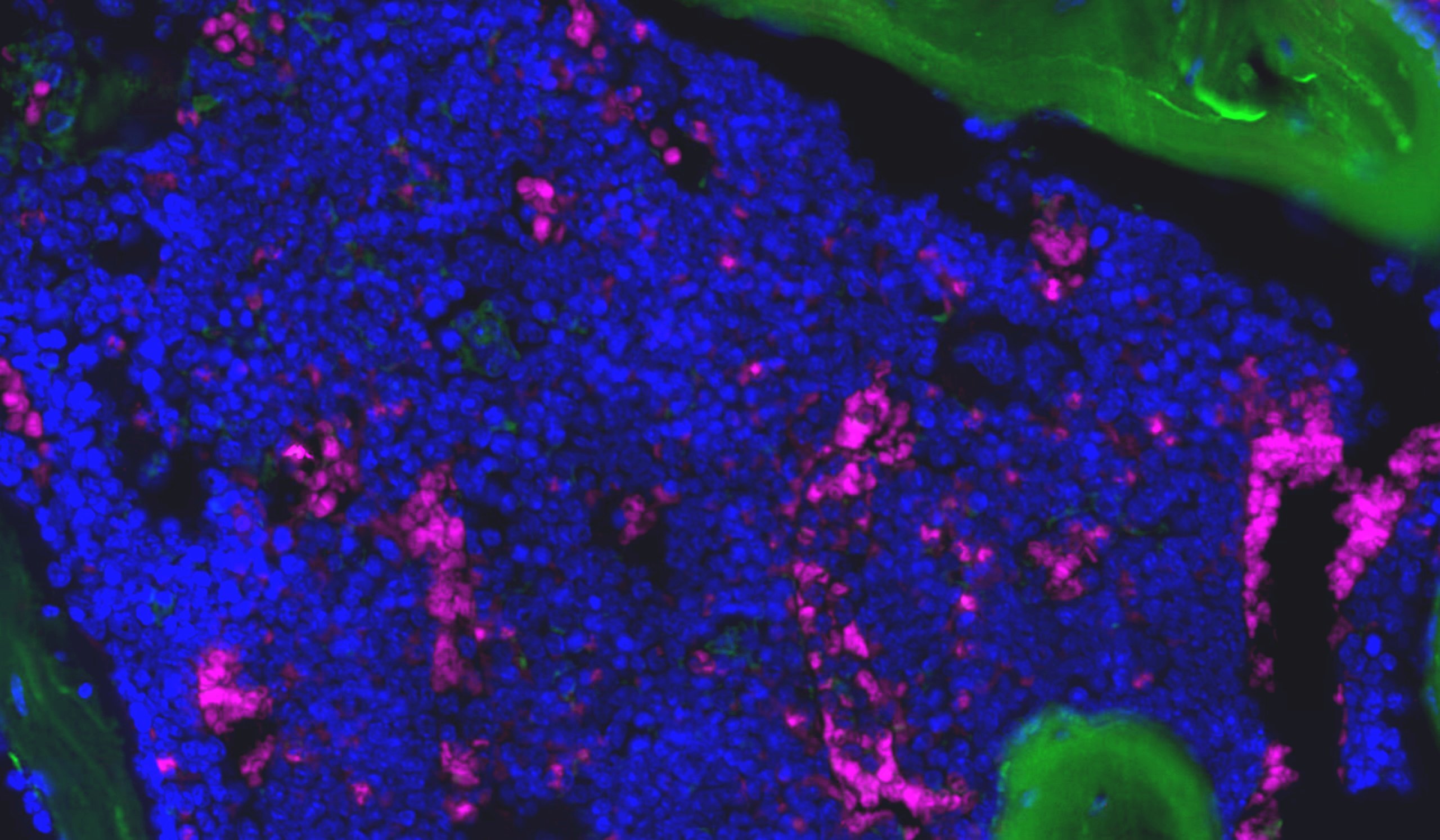

Supplement: S1 File — (ZIP) [file pone.0333897.s001.zip › Raw data/Figure7/IF-mice/Composite (RGB)1.jpg]

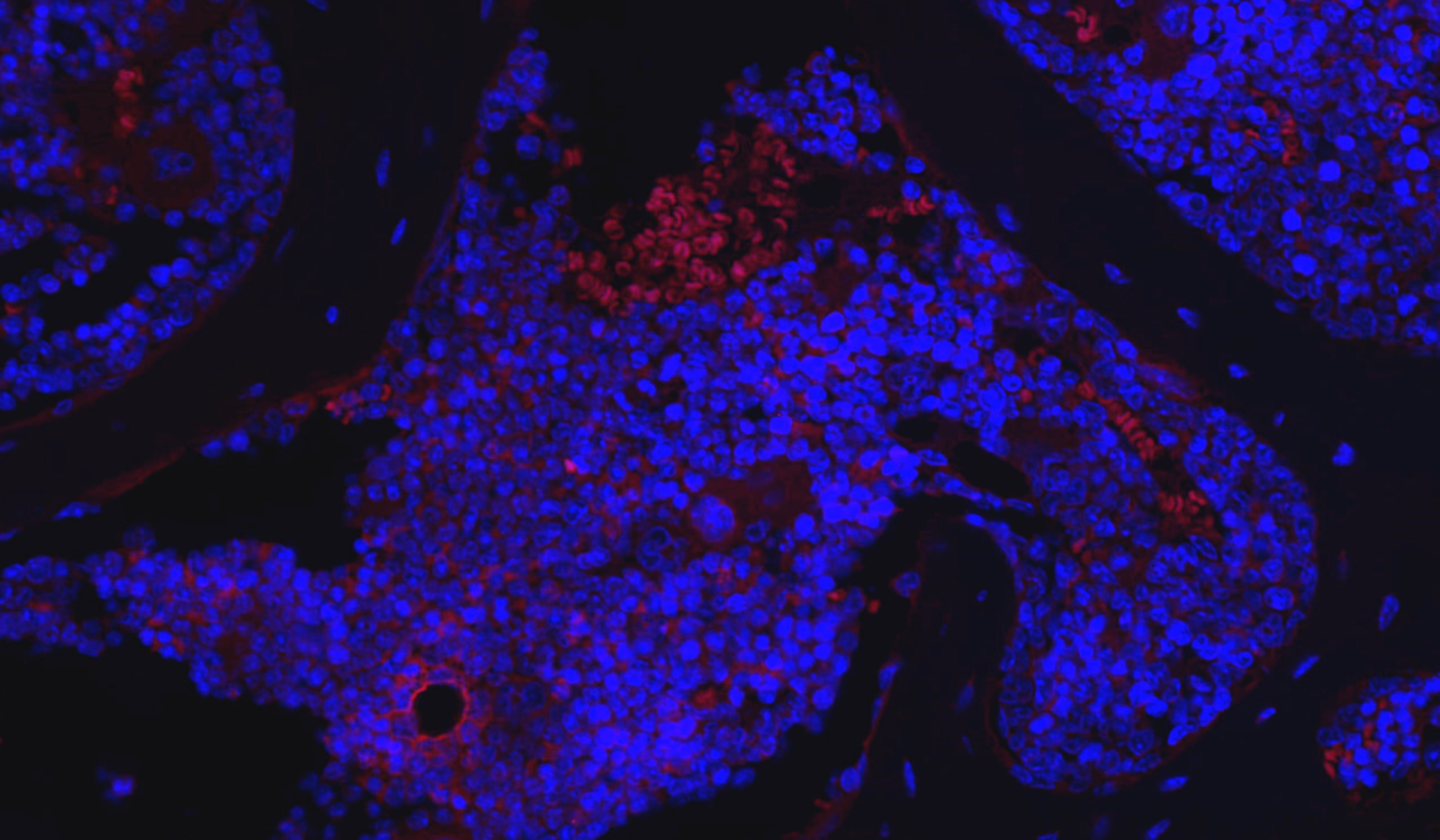

Supplement: S1 File — (ZIP) [file pone.0333897.s001.zip › Raw data/Figure7/IF-mice/Composite (RGB)2.jpg]

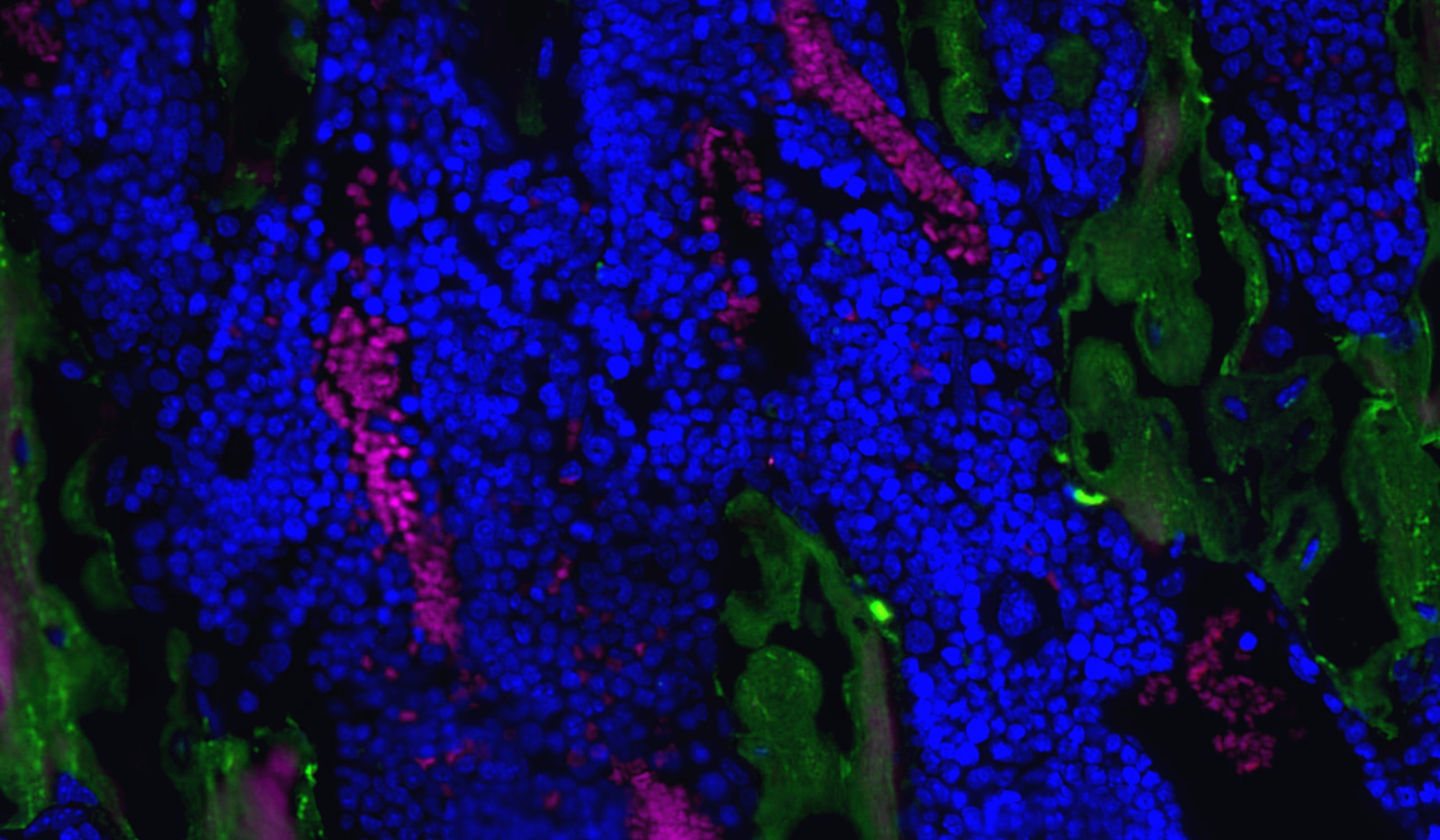

Supplement: S1 File — (ZIP) [file pone.0333897.s001.zip › Raw data/Figure7/IF-mice/Composite (RGB)3.jpg]
